# Root-microbe systems: the effect and mode of interaction of Stress Protecting Agent (SPA) *Stenotrophomonas rhizophila* DSM14405^T^

**DOI:** 10.3389/fpls.2013.00141

**Published:** 2013-05-14

**Authors:** Peyman Alavi, Margaret R. Starcher, Christin Zachow, Henry Müller, Gabriele Berg

**Affiliations:** ^1^Institute of Environmental Biotechnology, Graz University of TechnologyGraz, Austria; ^2^Austrian Centre of Industrial BiotechnologyGraz, Austria

**Keywords:** plant-microbe interaction, oilseed rape, PGPR, SPA, transcriptomics, root exudates, FISH–CLSM

## Abstract

*Stenotrophomonas rhizophila* has great potential for applications in biotechnology and biological control due to its ability to both promote plant growth and protect roots against biotic and a-biotic stresses, yet little is known about the mode of interactions in the root-environment system. We studied mechanisms associated with osmotic stress using transcriptomic and microscopic approaches. In response to salt or root extracts, the transcriptome of *S. rhizophila* DSM14405^T^ changed drastically. We found a notably similar response for several functional gene groups responsible for general stress protection, energy production, and cell motility. However, unique changes in the transcriptome were also observed: the negative regulation of flagella-coding genes together with the up-regulation of the genes responsible for biofilm formation and alginate biosynthesis were identified as a single mechanism of *S. rhizophila* DSM14405^T^ against salt shock. However, production and excretion of glucosylglycerol (GG) were found as a remarkable mechanism for the stress protection of this *Stenotrophomonas* strain. For *S. rhizophila* treated with root exudates, the shift from the planktonic lifestyle to a sessile one was measured as expressed in the down-regulation of flagellar-driven motility. These findings fit well with the observed positive regulation of host colonization genes and microscopic images that show different colonization patterns of oilseed rape roots. Spermidine, described as a plant growth regulator, was also newly identified as a protector against stress. Overall, we identified mechanisms of *Stenotrophomonas* to protect roots against osmotic stress in the environment. In addition to both the changes in life style and energy metabolism, phytohormons, and osmoprotectants were also found to play a key role in stress protection.

## Introduction

Crop cultivation in salinated soils is one of the major challenges facing agriculture today. Salinated areas are increasing world-wide and plants growing under saline or water-imbalance stress are more vulnerable to diseases caused by soil-borne pathogens (FAO, 2005). Biocontrol using salt-tolerant, plant growth-promoting rhizobacteria (PGPR) to protect plant roots against high salinity and pathogens offers sustainable solutions for plant protection, and *Stenotrophomonas rhizophila* is a model bacterium for a rhizosphere- and phylloplane-competent, salt-tolerant PGPR (Ryan et al., 2009; Berg et al., 2010, 2013). While the species *S. maltophilia* has become important as a nosocomial human pathogen, no pathogenic potential for humans has ever been observed in the related species *S. rhizophila* (Wolf et al., 2002). Moreover, both species can be easily distinguished by the production of the osmoprotective substance glucosylglycerol (GG) (only present in *S. rhizophila*) and the occurrence of specific multidrug-efflux pumps (only present in *S. maltophilia*) (Ribbeck-Busch et al., 2005).

Plant growth promotion by *S. rhizophila* strain DSM14405^T^ (syn. strain e-p10) was observed under greenhouse conditions (Schmidt et al., 2012) and in the highly salinated soils of Uzbekistan at levels up to 180% (Egamberdieva et al., 2011). Use of classical physiological and biochemical methods unveiled the mechanisms of plant growth promotion and biocontrol against soil-borne pathogens (Berg and Ballin, 1994; Kobayashi et al., 1995; Jacobi et al., 1996; Dunne et al., 2000; Suckstorff and Berg, 2003) as well as the production of high amounts of osmolytes trehalose and GG in response to salt stress (Roder et al., 2005). Next generation sequencing techniques have allowed for new possibilities to study plant-microbe interaction. For example, genome sequencing has given new insight into the genetic sources that provide beneficial plant-associated bacteria with traits such as plant growth promotion, protection against phytopathogens, and osmoprotection. In general, the described mode of action could be confirmed due to the presence of genes possibly responsible in the genome of *S. rhizophila* DSM14405^T^ (Berg et al., 2013). For example, *S. rhizophila* possesses genes responsible for the synthesis and transport of osmoprotective molecules out of the cell. In addition, it contains a number of genes involved in the biocontrol of soil-borne pathogens and important genes that aid in the competition for nutrients and niches as well. Additionally, *S. rhizophila* is equipped with several genes which may play a role in root colonization, such as those that encode the O-antigen, capsule polysaccharide biosynthesis pathways, hemagglutinin, and outer membrane adhesion proteins. However, despite this knowledge, there is still no evidence that these genes are involved in successful root-microbe interactions under salinated conditions. In addition to the high salinity, the role of root exudates for this interaction was pointed out in other studies (González-Pasayo and Martínez-Romero, 2000; rev. in Bais et al., 2006). The ability of cells to respond appropriately to changing environmental conditions can be investigated using a transcriptomic approach. This technique offers a new and powerful tool to evaluate these hypothetical mechanisms *in situ*, as shown already by van de Mortel et al. (2012) for the *Pseudomonas-Arabidopsis* and by López-Guerrero et al. (2012) for the *Rhizobium-Phaseolus* interaction.

The objective of our study was to investigate the response to changing environmental conditions associated with osmotic stress (1) salt stress and (2) root exudates to understand stress protection against changing osmotic conditions of roots by the endophytic bacterium *S. rhizophila* DSM14405^T^ in more detail. We hypothesized that there is a general response to changing osmolarities, but also a specific answer to each other of the two parameters which are important for colonizing the root system of plants.

## Materials and methods

### Treatment with oilseed rape exudates

Root exudates were collected from oilseed rape cultivar Californium (Kwizda, Austria) and grown for 14 days in gnotobiotic systems of 50 ml of sterilized vermiculite packaged in pots and covered with lids (Metro, Austria). Prior to sowing the seeds, about 50 ml of tap water was amended with 1/10 [v/v] of minimal medium (Gamborgs B5 basal salt mixture; Duchefa), and the seeds were surface-sterilized in sodium hypochlorite (10% wt/wt) for 10 min and washed successively with sterile water under sterile conditions. No seeds were sown in the control system. Plant and control systems were arranged in a replicate randomized block design and maintained at 20°C under 16-h light and 8-h dark conditions. After 14 days, plants were removed and the root exudates and liquid from the control system were collected in sterile bags and squeezed. To corroborate sterility, both root material and exudates were plated on nutrient agar. Root exudates were centrifuged (10 min, 5000 × g), and the supernatant was collected, filter-sterilized (first 0.45 μm, second 0.22 μm filter, Millipore), and stored at −20°C in the dark until use. *S. rhizophila* DSM14405^T^ was cultivated under agitation in 40 ml CAA (per liter: 5.0 g casamino acids, 1.54 g K_2_HPO_4_·3H_2_O, 0.25 g MgSO_4_·7H_2_O) and supplemented with 10 ml of the root exudates and the control liquid, respectively, at 30°C for 48 h. Cells were harvested using centrifugation at 2500 × g for 1 min for RNA extraction.

### Salt shock

*S. rhizophila* DSM14405^T^ was cultivated in 50 ml CAA under agitation at 30°C for 13 h (per liter: 5.0 g casamino acids, 1.54 g K_2_HPO_4_·3H_2_O, 0.25 g MgSO_4_·7H_2_O) until an optical density of OD_600_ 0.8 was reached. A final salt (NaCl) concentration of 3% in the medium was reached by using a sterile concentrated sodium chloride stock solution (0.3 g l^−1^). After 2.7 h cultivation in the medium containing 3% salt, the *S. rhizophila* DSM14405^T^ culture (OD_600_ = 0.9) was used for RNA extraction. Two independent replicates were performed as described above.

### RNA extraction and transcriptomic analyses

RNA was extracted using the RNAprotect® Bacteria Reagent (Qiagen, Hilden, Germany). Total rRNA was removed and mRNA was enriched using the MICROBExpress kit, according to the manufacturer's protocol (Invitrogen, Carlsbad, USA). The mRNA was sequenced using LGC Genomics (Berlin, Germany), and data collection was performed using MicroDiscovery (Berlin, Germany). The data used for assessing the changes in gene transcription correspond to normalized values for the number of reads that uniquely mapped to each CDS. Transcription fold change for each CDS was assessed by dividing the corresponding value from the cells that were either treated with root exudates or exposed to salt shock by those from the control group. Of the total genes either up or down-regulated, only those showing fold changes greater than or equal to 1.5 and less than or equal to 0.6 were considered significantly impacted.

### Germination pouch colonization assay

A batch of 200 oilseed rape seeds were surface-sterilized with 40 ml of 3% NaOCl for 1 min and subsequently washed twice with 40 ml of water for 1 min each time. Surface-sterilized seeds were inoculated with *S. rhizophila* DSM14405^T^ by incubating in a 2 ml cell suspension containing 10^7^ CFU ml^−1^. The control included seeds treated with 0.85% NaCl. Twelve seeds per treatment were placed into 2 (6 seeds per pouch) sterile Cyg™ germination pouches (Mega International, West St. Paul, MN, USA) wetted with 10 ml of sterilized deionized water or 1.25% NaCl solution. Germination pouches were then placed in sterile, aseptically sealed containers and placed in a growing chamber for 9 days with controlled day and night settings (12 h of light at 25°C and 12 h of dark at 20°C). After 9 days of growth, roots of 3 seedlings were combined for determination of cell counts resulting in 4 replicates per individual treatment. All root material was cut and transferred to Whirlpak® bags (Carl Roth, Karlsruhe, Germany) containing 2 ml of 0.85% NaCl solution. The roots in the bags were then crushed using a pestle to form a homogenous suspension, which was subsequently serially diluted and drop-streaked onto LB Petri dishes. The plates were then incubated at 30°C for 24 h.

### Fluorescent *in situ* hybridization (FISH)

To study the oilseed rape colonization ability of *S. rhizophila* DSM14405^T^ using confocal microscopy, the oilseed rape roots grown in seed germination pouches were fixed with 4% paraformaldehyde/phosphate buffered saline (PBS) (3:1 vol/vol). The control group contained roots without bacterial treatment. The fixed samples were then stored in PBS/ 96% ethanol (1:1) at −20°C. The FISH probes were purchased from genXpress® (Wiener Neudorf, Austria), and the in-tube FISH was performed as described by Cardinale et al. (2008). The FISH probes used for the hybridization step were labeled with the fluorescent dye Cy3 and included EUB338 (Amman et al., 1990), EUB338 II, and EUB338 III (Daims et al., 1999), all directing eubacteria. An equimolar ratio of the FISH probes was used for the hybridization step to detect *S. rhizophila* DSM14405^T^. In this step, 30% formamide was added to the samples which were then subsequently incubated in a water bath (43°C) for 90 min. After hybridization, the samples were washed at 44°C for 15 min. Microscopy and image capturing were performed using a Leica TCS SPE confocal microscope (Leica Microsystems, Wetzlar, Germany) with the Leica ACS APO 63X OIL CS objective (NA: 1.30). A z-step of 0.4–0.8 μm was applied to acquire confocal stacks.

## Results

### Transcriptional response of *S. rhizophila* DSM14405^T^ to salt stress

**Figure 1:**
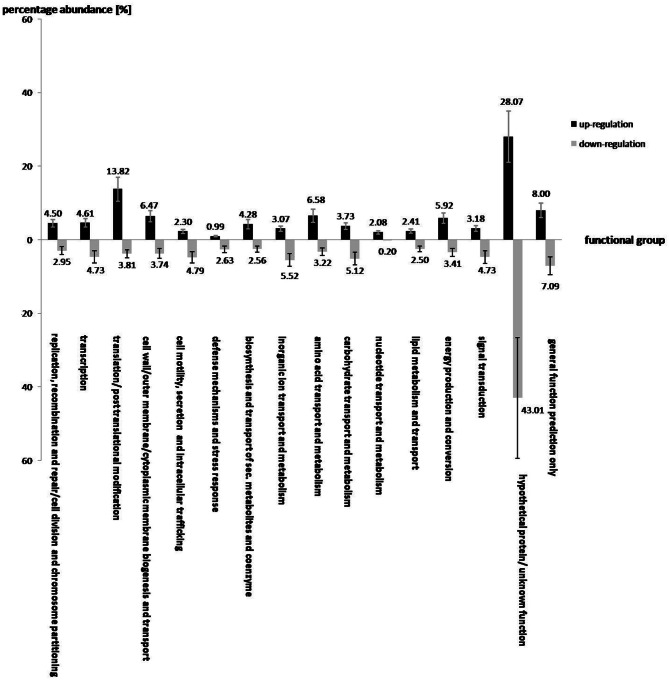
**The effect of salt shock on the gene expression of various functional gene groups in *S. rhizophila* DSM14405^T^.** A total of 912 and 1521 genes were significantly up and down-regulated, respectively. The impact of salt stress on most functional gene groups is clearly pronounced, as a given functional group shows either an increase or decrease in the transcription of genes belonging to that group. Genes involved in translation, synthesis of the cell wall, outer or cytoplasm membrane, nucleotide and amino acid transport and metabolism, energy production and conversion are up-regulated. In contrast, genes involved in cell motility, secretion, and intracellular trafficking, defense mechanisms, and transport and metabolism of carbohydrates and inorganic ions are down-regulated. Genes involved in lipid metabolism, and the hypothetical genes are rather ambiguously affected by salt stress, as some of these are up and others down-regulated. The values above each column correspond to the percentage abundance of the corresponding functional group relative to the total count of the up and down-regulated genes. The transcription fold change for each CDS corresponds to the ratio calculated for *S. rhizophila* under salt shock compared with the control. Data are presented as the mean value of two independent replicates. The error bar shown on each functional group corresponds to the mean value of errors for all genes belonging to that functional group.

Under salt stress of 3% NaCl, a total number of 912 and 1521 genes of *S. rhizophila* DSM14405^T^ were significantly up and down-regulated, respectively. The impact of salt shock on the transcription of *S. rhizophila* DSM14405^T^ with respect to various functional gene groups is shown in Figure 1. The majority of functional groups were strongly affected, such as up-regulated genes involved in translation, synthesis of the cell wall, outer or cytoplasm membrane, nucleotide and amino acid transport and metabolism, and the production and conversion of energy. In contrast, genes involved in cell motility, secretion, intracellular trafficking, defense mechanisms, and the transport and metabolism of carbohydrates and inorganic ions are down-regulated. Moreover, genes responsible for lipid metabolism and hypothetical genes are somewhat ambiguously affected by salt stress as some are up while others down-regulated.

Of the genes that are significantly impacted by salt stress in *S. rhizophila* DSM14405^T^ (Table 1, Tables S1A, S1B), a number of those responsible for general and specific stress responses are up-regulated. While *surA* and *dnaJ* code for general stress chaperones, *ggpS* and *ycaD* build a well-known salt stress response mechanism in *S. rhizophila* DSM14405^T^ through the synthesis of the osmolyte GG and show a fold change of 8.3, and 7.7, respectively (Hagemann et al., 2008). Moreover, two genes responsible for cold shock, *deaD* and *cspA*, are also strongly up-regulated under salt stress. In addition, the transcription of *ousA* that codes for an osmotic stress protein is positively affected as well. Cellular ion exchange mechanisms and some iron uptake genes are also up-regulated in *S. rhizophila* DSM14405^T^ as the result of salt stress.

**Table 1:** **Selected *S. rhizophila* DSM14405^T^ genes with known biological roles impacted by salt shock**.

**Gene**	**(Putative) Product**	**Transcription fold change**	**Biological function**
*ggpS*	Glucosylglycerol-phosphate synthase	8.3	Salt shock response protein
*ycaD*	MFS-type transporter	7.7	Salt shock response protein transporter
*xanA*	Hosphohexane mutases	3.0	Xanthan biosynthesis; biofilm formation
*xanB*	Xanthan biosynthesis protein xanB	2.9	Xanthan biosynthesis; biofilm formation
*rmlC*	dTDP-4-dehydrorhamnose 3,5-epimerase	2.3	Xanthan biosynthesis; biofilm formation
*algJ*	Alginate biosynthesis protein	3.2	Alginate biosynthesis
Sr14405 2749	TVISS effector, Hcp1 family protein	2.6	Type VI secretion system
Sr14405 2755	Rhs element Vgr protein	4.2	Type VI secretion system
Sr14405 2761	Rhs element Vgr protein	3.4	Type VI secretion system
*icmF*	TVISS protein	5.0	Type VI secretion system
Sr14405 2781	TVISS-associated protein, ImpA family	2.4	Type VI secretion system
Sr14405 2791	Rhs element Vgr protein	6.8	Type VI secretion system
*deaD*	Cold-shock DEAD box protein A homolog	8.4	Cell shock response
*cspA*	Major cold shock protein	4.3	Cell shock response
Sr14405 1916	Beta-lactamase L2 protein	6.3	Antibiotic resistance
*tetA*	Tetracycline resistance protein	3.8	Antibiotic resistance
Sr14405 1293	Bacterioferritin-associated ferredoxin	2.1	Iron uptake and transport
bfr	Bacterioferritin	5.9	Iron uptake and transport
hisl	Histidine biosynthesis bifunctional protein	4.6	Histidine biosynthesis
*ousA*	Osmoprotectant uptake system protein	4.2	Osmotic stress response
*surA*	Chaperone protein	3.8	Cellular stress response
*dnaJ*	Chaperone	3.1	Stress response
*ompW*	Outer membrane protein	3.6	Transport
*oprF*	Outer membrane protein	2.6	Transport
*ftsQ*	Cell division protein	3.4	Cell division
*ftsA*	Cell division protein	2.3	Cell division
*ftsY*	Cell division protein	2.6	Cell division
*ftsZ*	Cell division protein	2.0	Cell division
*lptF*	Lipopolysaccharide export system permease protein	4.5	Cell wall transport
*IptG*	Lipopolysaccharide export system permease protein	3.3	Cell wall transport
Sr14405 2454	Peptidoglycan-associated outer membrane lipoprotein	2.5	Cell wall protein
*mltD*	Muramidase	3.2	Bacterial cell wall biodegradation
Sr14405 1936	Peptidoglycan-associated lipoprotein	2.8	Cell wall structure protein
Sr14405 4324	Cell morphology protein	2.7	Unknown
*clcA*	H(+)/Cl(−) exchange transporter	2.7	Ion regulation
*kefA*	Potassium efflux system	2.3	Ion regulation
*flgA*	Flagellar basal body P-ring biosynthesis	0.5	Flagellar-driven motility
*flgC*	Flagellar basal body P-ring biosynthesis	0.3	Flagellar-driven motility
*flgG*	Flagellar basal body P-ring biosynthesis	0.3	Flagellar-driven motility
*flgF*	Flagellar basal body P-ring biosynthesis	0.3	Flagellar-driven motility
*fliF*	Flagellar basal body P-ring biosynthesis	0.3	Flagellar-driven motility
*flhA*	Flagellar biosynthesis	0.3	Flagellar-driven motility
*flhB*	Flagellar biosynthesis	0.3	Flagellar-driven motility
*cfaB*	CFA/I fimbrial subunit B	0.3	Fimbriae synthesis
*cfaC*	CFA/I fimbrial subunit C	0.4	Fimbriae synthesis
*csoB*	Fimbrial subunit B	0.2	Fimbriae synthesis
Sr14405 3215	Capsule polysaccharide biosynthesis protein	0.1	Capsule biosynthesis
Sr14405 3217	Putative UDP-glucose 4-epimerase	0.2	Capsule biosynthesis
*wzc*	Tyrosine-protein kinase	0.2	Capsule biosynthesis

*The values for fold changes correspond to the S. rhizophila DSM14405^T^ subjected to the 3% NaCl shock compared to the control*.

Although unable to synthesize xanthan, *S. rhizophila* DSM14405^T^ possesses some of the up-regulated xanthan-coding genes including *xanA*, *xanB*, and *rmlA*C. These genes are involved in biofilm formation in addition to their role in xanthan biosynthesis (Huang et al., 2006). Likewise, the alginate coding gene *algJ* shows a fold change of 3.2 as a result of salt shock. Alginate is an exopolysaccharide involved in the development and architecture of biofilms that protect bacteria from antibiotics and other harmful environmental factors (Monday and Schiller, 1996; Stapper et al., 2004). Furthermore, specific secretion and transport systems such as those that code for the type VI secretion system (TVISS) are strongly up-regulated in *S. rhizophila* DSM14405^T^ under salt shock, however, it should be noted that closely related plant-associated *Stenotrophomonas* strains such as *S. maltophilia* R551-3 lack the TVISS. Moreover, genes involved in the conversion and transport of substances through the cell wall and those responsible for cell division are also up-regulated.

Genes responsible for flagellar apparatus and fimbriae-biosynthesis genes are comparatively down-regulated in *S. rhizophila* DSM14405^T^ under salt shock. Similarly, salt shock also negatively impacted the predicted capsule biosynthesis genes.

### Transcriptional response of *S. rhizophila* DSM14405^T^ to root exudates

**Figure 2:**
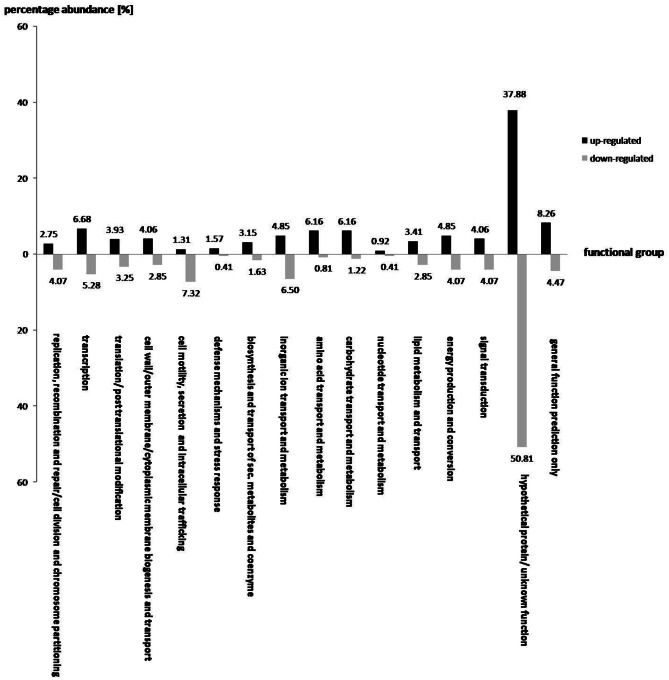
**The effect of oilseed rape seedling exudates on gene expression of various functional gene groups in *S. rhizophila* DSM14405^T^.** A total of 763 and 246 genes were significantly up and down-regulated, respectively. While some functional groups are both positively and negatively regulated by root exudates, others show a clear and pronounced alteration, as the majority of the corresponding genes are either up or down-regulated. For example, genes responsible for amino acid, nucleotide, and carbohydrate transport and metabolism, and those involved in cell wall, outer-membrane or cytoplasmic membrane biogenesis and transport as well as genes responsible for the transport of secondary metabolites and coenzymes are mainly up-regulated. In contrast, genes involved in cell motility and secretion, and those responsible for the transport and metabolism of inorganic ions are mainly down-regulated. The value above each column corresponds to percentage abundance of the corresponding functional group in the total count of the up or down-regulated genes.

A total of 763 and 246 genes were significantly up and down-regulated, respectively, as a result of the addition of oilseed-rape root exudates (Figure 2). In general, the effect of root exudates on the functional groups is indeterminate, as some genes of a particular group are up-regulated while others are transcribed in lesser numbers. However, some functional groups are equally affected by plant root exudates, as the transcription of almost all corresponding gene sequences is either up or down-regulated. For instance, as shown in Figure 2, root exudates have only a positive effect on the transcription of genes responsible for amino acid, nucleotide, and carbohydrate transport and metabolism, as well as biogenesis of cell membranes, transport of substances through the cell, and the genes responsible for the transport of secondary metabolites and coenzymes. Conversely, genes involved in the secretion, transport, and metabolism of inorganic ions as well as in cell motility are mainly down regulated in response to root exudate stress.

Of those genes with a significant transcription fold change discussed above, some code for products with a known physiological function and are presented in Table 2. The complete list of *S. rhizophila* DSM14405^T^ genes with a significant transcription fold change is presented in Tables S2A, S2B. Cell wall breakdown and cell adherence are early and crucial steps in host-plant colonization. As presented in Table 2, the treatment of *S. rhizophila* DSM14405^T^ cells with oilseed rape seedling exudates resulted in enhanced expression of *cbg-1* and *xynB* that code for beta-glucosidase and xylanase B, respectively, and are involved in cell wall breakdown. Furthermore, both these genes are conserved among plant-associated *Stenotrophomonas* strains as they are present in both *S. rhizophila* DSM14405^T^ and the plant-benefiting *S. maltophilia* R551-3, but absent from the human-pathogenic *S. maltophilia* K279a. In addition, Sr14405 2818, which is also up-regulated by 2.4 folds, codes for an adhesin protein and is homologous to the haemagglutinin-like protein coding gene from the human-pathogenic *S. maltophilia* K279a (Table 2). Other up-regulated genes include two adjacent genes, *mdtI* and *mdtJ* that both code for spermidine export proteins. Spermidine is a plant growth regulator and has been recently shown to strongly promote the growth of arugula plants (Al-Whaibi et al., 2012). Moreover, several genes that code for multidrug resistance pumps, efflux transporters, heavy metal transport systems, and resistance against antibiotics are positively affected by seedling exudates.

**Table 2:** **Selected *S. rhizophila* DSM14405^T^ genes with known biological roles impacted by plant root exudates**.

**Gene**	**(Putative) Product**	**Transcription fold change**	**Biological function**
*mdtI*	Spermidine export protein	6.3	Export of the plant growth regulator spermidine
*mdtJ*	Spermidine export proteins	7.6	Export of the plant growth regulator spermidine
Sr14405 2818	Adhesin	2.4	Host cell surface attachment/colonization
*cbg-1*	Beta-glucosidase	1.7	Plant cell wall biodegradation/colonization
*xynB*	Xylanase B	1.6	Plant cell wall biodegradation/colonization
Sr14405 4324	Cell morphology protein	2.2	Unknown
Sr14405 1672	Generally characterized MFS-type transporter	3.0	Antibiotic resistance
Sr14405 1673	Multidrug synthesis protein	8.8	Antibiotic resistance
Sr14405 2718	Multidrug synthesis protein	3.5	Antibiotic resistance
*tetX*	Tetracycline resistance protein	3.5	Antibiotic resistance
Sr14405 4658	Acriflavin resistance protein	1.6	Antibiotic resistance
Sr14405 2827	Heavy metal transport and detoxcification protein	2.0	Heavy metal efflux system
Sr14405 1538	Efflux transporter	1.5	Efflux of unknown target
*flgA*	Flagellar basal body P-ring biosynthesis	0.5	Flagellar-driven motility
*flgC*	Flagellar basal body P-ring biosynthesis	0.5	Flagellar-driven motility
*flgG*	Flagellar basal body P-ring biosynthesis	0.5	Flagellar-driven motility
*flgF*	Flagellar basal body P-ring biosynthesis	0.5	Flagellar-driven motility
*fliF*	Flagellar basal body P-ring biosynthesis	0.5	Flagellar-driven motility
*flhA*	Flagellar biosynthesis	0.6	Flagellar-driven motility
*flhB*	Flagellar biosynthesis	0.5	Flagellar-driven motility
*cfaB*	CFA/I fimbrial subunit B	0.5	Fimbriae synthesis
*csoB*	Fimbrial subunit B	0.4	Fimbriae synthesis
Sr14405 1293	Bacterioferritin-associated ferredoxin	0.6	Iron uptake and transport
Sr14405 1746	Heme oxygenase	0.4	Iron bioavailability
*fpvA*	Ferripyoverdine	0.5	Iron uptake and transport
Sr14405 4245	Outer-membrane hemin receptor	0.4	Iron uptake and transport

*The values for fold changes correspond to the S. rhizophila DSM14405^T^ treated with plant root exudates compared to the control*.

*S. rhizophila* DSM14405^T^ contains two flagella-encoding gene blocks that are almost entirely negatively affected by the addition of oilseed rape seedling exudates. The complete list of the flagellar apparatus-coding genes that are down-regulated is not confined to those noted in Table 2, and is presented in Tables S2A, S2B. Likewise, the expression of the genes responsible for fimbriae-driven cell motility, such as *cfaB* and *csoB* is negatively impacted by seedling exudates. Moreover, genes involved in the uptake, transport, and bioavailability of iron are also down-regulated.

### Similarities in the transcriptional response of *S. rhizophila* DSM14405^T^ to salt and root exudates

**Figure 3:**
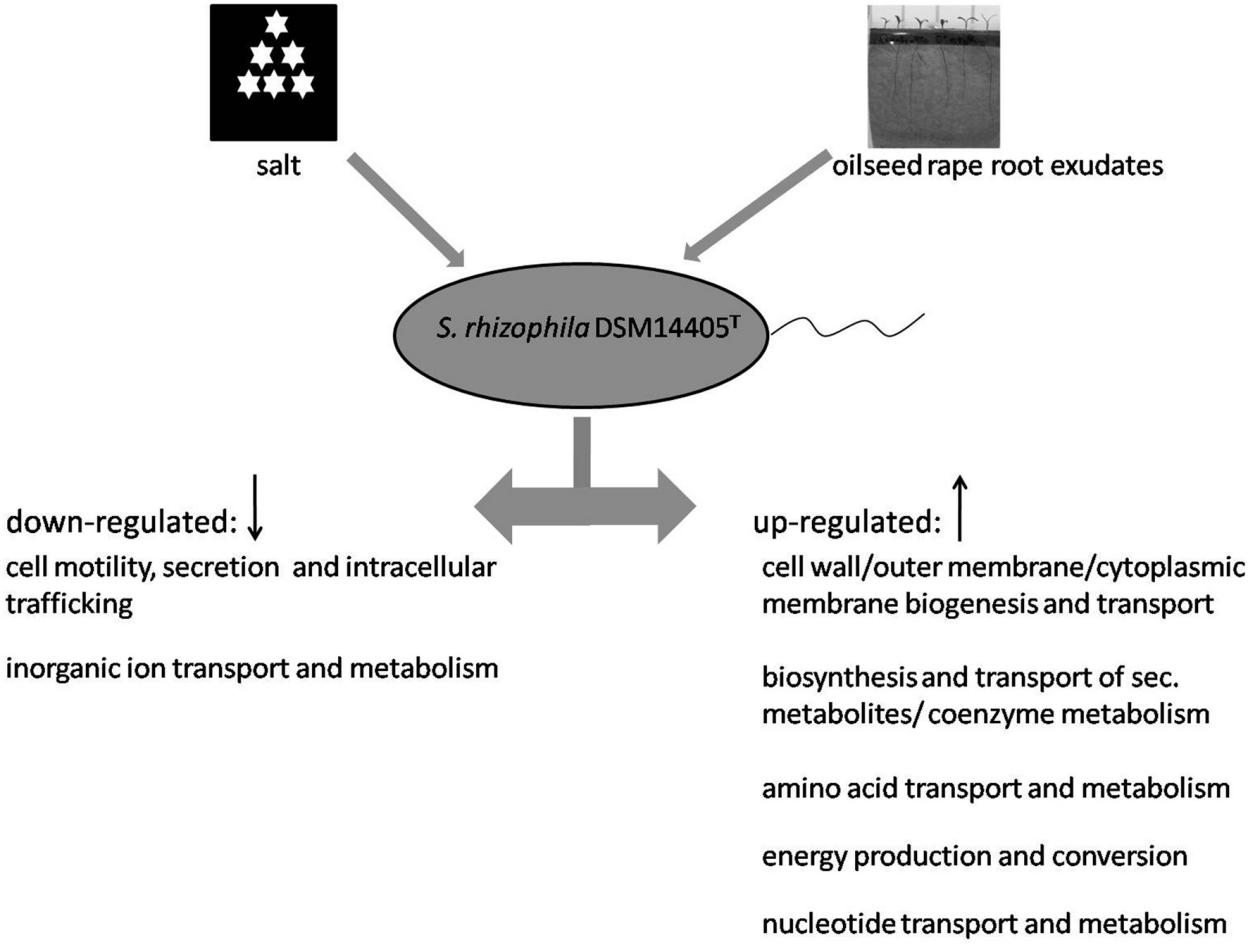
**Model showing the response of *S. rhizophila* DSM14405^T^ to osmotic stress: salt shock and root exudates.** Functional gene groups shared in the response to oilseed rape root exudates and salt shock are presented. Several functional gene groups are up-regulated as a result of both oilseed rape root exudates and salt shock including those responsible for the synthesis and transport of cell wall, outer membrane, and cytoplasmic membrane, the metabolism and transport of amino acids, nucleotide, and secondary metabolites, and energy production. In contrast, genes responsible for cell motility, secretion and intracellular trafficking, and the transport and metabolism of inorganic ions are down-regulated.

In response to both oilseed rape root exudates and salt shock, *S. rhizophila* DSM14405^T^ copes with osmotic stress in a surprisingly similar way through the alteration of gene expression. Numerous functional gene groups are up-regulated in response to osmotic stress factors and include those involved in energy production, as well as those involved in the synthesis and transport of cell wall, outer membrane, and cytoplasmic membrane, and those responsible for the metabolism and transport of amino acids, nucelotides, and secondary metabolites (Figure 3). Conversely, genes responsible for cell motility, secretion, intracellular trafficking, and the transport and metabolism of inorganic ions are down-regulated under both salt stress and treatment with root exudates.

### Colonization patterns of *S. rhizophila* DSM14405^T^ on roots under stress

**Figure 4:**
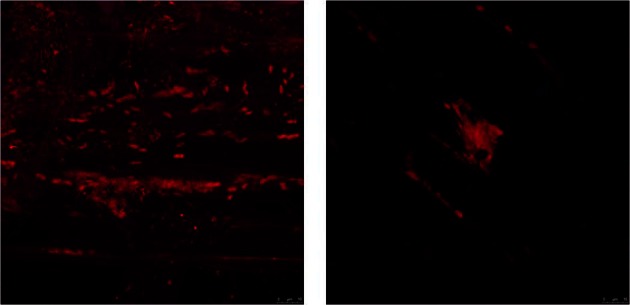
**The impact of salt stress on the capability of *S. rhizophila* DSM14405^T^ to colonize the oilseed rape rhizosphere visualized using FISH-CLSM**. *S. rhizophila* DSM14405^T^ intensely colonizes the oilseed rape rhizosphere (left) while the treatment of seeds with 1.25% NaCl (right) severely decreases the colonization capability. An equimolar ratio of the FISH probes EUB338, EUB338 II, and EUB338 III labeled with the fluorescent dye Cy3 was used in the hybridization step. Microscopic images were captured using a Leica TCS SPE confocal microscope. The Leica ACS APO 63X OIL CS objective (NA: 1.30) was used to acquire confocal stacks by applying a z-step of 0.4–0.8 μm.

*S. rhizophila* DSM14405^T^ intensely colonizes oilseed rape plants, as revealed by the cell count of log_10_ 9.47 CFU g^−1^ root fresh weight (±0.08) for seeds treated with deionized water. The treatment of seeds with 1.25% NaCl, however, decreased the colonization ability by nearly half resulting in a cell count of log_10_ 9.09 CFU g^−1^ root fresh weight (±0.18). Furthermore, microscopic images captured using FISH combined with confocal laser scanning microscopy (CLSM) also revealed a significant decrease in the colonization of oilseed rape roots treated with 1.25% NaCl (Figure 4).

## Discussion

We studied the response of *S. rhizophila* DSM14405^T^ to osmotic changes in the form of plant root exudates and salt shock at both the physiological and molecular level. Even though we found a notable similarity in how the cell copes with these stressors, the individual responses included a great deal of specificity at the gene level thus. The response of *S. rhizophila* DSM14405^T^ to both oilseed rape root exudates and salt corresponds with several functional gene groups including those responsible for the synthesis and transport of cell wall, outer membrane, and cytoplasmic membrane, the metabolism and transport of amino acids, nucleotide, and secondary metabolites, energy production, cell motility, secretion and intracellular trafficking, and the transport and metabolism of inorganic ions. For *S. rhizophila* treated with root exudates, however, the shift from the planktonic lifestyle to a sessile one as expressed in the down-regulation of flagellar-driven motility is targeted to colonize the plant host, and is well in accordance with the observed positive regulation of host colonization genes. In addition to the changes in behavior and lifestyle of the bacterium, several bioactive substances were identified as key factors in stress protection. The first among them is the plant growth regulator, spermidine. Although this substance is known to strongly promote growth, this is the first evidence to show its involvement in stress protection of roots. The second group includes osmoprotective substances which were both produced and excreted in high volumes as described earlier (Roder et al., 2005).

Spermidine is a well-known plant growth regulator and has been revealed to play a critical role in plant embryo development (Imai et al., 2004). Moreover, it has been recently shown to strongly promote the growth of arugula plants (Al-Whaibi et al., 2012). In addition, spermidine affects biofilm formation in various bacterial species via multiple pathways that involve both transport and signaling networks (McGinnis et al., 2009). As a result, enhanced biofilm formation or possible plant growth regulation resulting from the up-regulation of *S. rhizophila* spermidine export genes would well-serve the lifestyle shift that ultimately leads to efficient colonization of the plant host in the presence of oilseed rape exudates. Spermidine was found to prolong the life span of several eukaryotic model organisms including yeasts, nematodes, flies, and plants as well as significantly reduce age-related oxidative protein damage in mice which could indicate a potential universal anti-aging drug for eukaryotes (Imai et al., 2004; Eisenberg et al., 2009).

GG and trehalose are well-studied general osmoprotective substances that protect cells from high salt concentrations (Ferjani et al., 2003; Hincha and Hagemann, 2004). While both species produce trehalose, GG is synthesized exclusively in *S. rhizophila* thus distinguishing itself from the pathogenic *S. maltophilia* (Ribbeck-Busch et al., 2005; Roder et al., 2005). In *S. rhizophila* DSM14405^T^, *ggpS* and *ycaD* are both strongly up-regulated under 3% salt and are essential for the synthesis and transport of GG. This finding corresponds completely with both the general role of GG as a cell protector and previous findings that the amount of GG excreted into the medium increases substantially in comparison with intracellular GG content resulting from a shift of lower (less than 2%) to higher salt concentrations (Roder et al., 2005). Thus, GG production is the specific mechanism of *S. rhizophila* DSM14405^T^ to cope with salt stress.

TVISS genes represent a novel key virulence system used by many important pathogenic bacteria in eukaryotic host infection (Bingle et al., 2008; Pieper et al., 2009) and are intensely up-regulated under salt shock. In addition, plant growth promotion increased up to 180% in the highly salinated soils of Uzbekistan in the presence of *S. rhizophila* DSM14405^T^ (Egamberdieva et al., 2011). Similarly, Schmidt et al. (2012) reported that this plant growth promotion effect was more pronounced in soil than under gnotobiotic conditions suggesting it is due to the control of diseases and deleterious microorganisms. Together with the absence of TVISS genes from the other known plant-beneficial *Stenotrophomonas* strains with no plant growth promoting effect under saline conditions, these findings imply that the salt-stimulated *S. rhizophila* DSM14405^T^ TVISS is indirectly harnessed to promote plant growth by eliminating harmful and deleterious microorganisms in soil.

The treatment of *S. rhizophila* DSM14405^T^ with oilseed rape exudates resulted in the down-regulation of iron uptake and transport genes. This change is possibly due to the fact that once treated with oilseed rape exudates, *S. rhizophila* is provided with biologically available iron bound with plant siderophores resulting in less demand for the synthesis of bacterial siderophores to bind and uptake biologically unavailable iron ions present in the medium. Moreover, treatment with root exudates resulted in the up-regulation of several multidrug resistance pumps thus demonstrating that the role of the multidrug pumps is not confined to the export of antibiotics out of the cell, but also includes a more general function with the transport of other substances once in a hyperosmotic environment.

Several questions still remain, such as the reason for positive regulation of several genes responsible for iron uptake and transport, as well as the reason for cell division under salt shock or the role of other remaining genes that are significantly up or down-regulated by osmotic stress factors. However, this work has shed light on the so far unknown mode of action of *S. rhizophila* DSM14405^T^ to a-biotic changes by unveiling the mechanisms that are harnessed to establish in highly salinated plant root ecosystems.

### Conflict of interest statement

The authors declare that the research was conducted in the absence of any commercial or financial relationships that could be construed as a potential conflict of interest.

## Supplementary material

Table S1A **Significantly up-regulated genes in *S. rhizophila* DSM14405^T^ under salt shock**.

Table S1B **Significantly down-regulated genes in *S. rhizophila* DSM14405^T^ under salt shock**.

Table S2A **Significantly up-regulated genes in *S. rhizophila* DSM14405^T^ under treatment with root exudates**.

Table S2B **Significantly down-regulated genes in *S. rhizophila* DSM14405^T^ under treatment with root exudates**.
