# Supplementary material for: Root-microbe systems: the effect and mode of interaction of Stress Protecting Agent (SPA) Stenotrophomonas rhizophila DSM14405T
Source: Front Plant Sci. 2013 May 14;4:141. doi: 10.3389/fpls.2013.00141 (PMC3653106; doi:10.3389/fpls.2013.00141)
Supplement: Table S1A — Significantly up-regulated genes in S. rhizophila DSM14405T under salt shock. [file DataSheet1.ZIP › 51567__Table_S2B.PDF]

Supplementary Table 2b: significantly down-regulated genes in *S. rhizophila* DSM14405T under treatment with root exudates

locus tag	gene	fold change	product
1054	1054	0.4	Hypothetical Protein 1054
1062	1062	0.6	Hypothetical
1072	acpP2	0.6	Acyl carrier protein 2
1089	ycgL	0.6	UPF0745 protein PXO_02884
1091	1091	0.6	Hypothetical
1123	1123	0.4	Hypothetical Protein 1123
1157	ycgL	0.5	Uncharacterized protein ycgL
1202	1202	0.6	Hypothetical Protein 1202
1207	1207	0.6	Hypothetical Protein 1207
1230	1230	0.6	Hypothetical
1233	1233	0.6	Conserved Hypothetical Protein
1234	1234	0.4	Hypothetical
1244	sigW	0.6	RNA polymerase sigma factor sigW
1260	1260	0.6	Hypothetical
1276	1276	0.5	Hypothetical Protein 1276
1293	1293	0.6	Bacterioferritin-Associated Ferredoxin
1310	1310	0.6	Multidrug resistance protein mdtA
1357	accC	0.6	Biotin carboxylase
1361	arsB	0.6	Arsenite resistance protein ArsB
1375	ptxR	0.4	HTH-type transcriptional regulator ptxR
1454	1454	0.6	Hypothetical
1459	1459	0.5	Hypothetical Protein 1459
1463	ydfG	0.6	Uncharacterized protein ydfG
1477	1477	0.5	Hypothetical Protein 1477
1484	1484	0.6	Hypothetical
1537	yknY	0.5	Uncharacterized ABC transporter ATP-binding protein YknY
1562	ptrB	0.6	Uncharacterized peptidase y4nA
1567	ybaN	0.5	Inner membrane protein ybaN
1595	mrdB	0.6	Rod shape-determining protein rodA
1654	insK	0.3	Putative transposase InsK for insertion sequence element IS150
1661	frmR	0.6	Transcriptional repressor frmR
1671	nahR	0.6	HTH-type transcriptional activator nahR
1691	1691	0.5	Hypothetical
1694	citN	0.6	Citrate transporter
1695	oprO	0.6	Porin O

1699	agmR	0.6	Glycerol metabolism activator
1700	dctB	0.6	C4-dicarboxylate transport sensor protein dctB
1701	oprP	0.6	Porin P
1702	dctA	0.6	C4-dicarboxylate transport protein
1742	fecI	0.6	ECF Subfamily RNA Polymerase Sigma Factor
1746	1746	0.4	Heme Oxygenase
1747	ybaN	0.4	Inner membrane protein ybaN
1752	1752	0.6	Hypothetical
1773	rubA2	0.3	Rubredoxin-2
1784	1784	0.6	Hypothetical
1802	cfaB	0.5	CFA/I fimbrial subunit B
1805	csoB	0.4	CS1 fimbrial subunit B
1807	1807	0.6	Hypothetical Protein 1807
1861	1861	0.6	Rieske Domain-Containing Protein
1907	fecR	0.4	Protein fecR
1917	ggpS	0.6	Glucosylglycerol-phosphate synthase
1982	nudE	0.5	ADP compounds hydrolase nudE
1986	1986	0.4	Hypothetical
2012	insF1	0	Transposase insF for insertion sequence IS3A
2015	2015	0.6	Conserved Hypothetical Protein
2018	2018	0.6	Hypothetical Protein 2018
2028	2028	0.5	Hypothetical
2062	2062	0.5	Hypothetical
2065	2065	0.6	Hypothetical
2068	2068	0.5	Hypothetical
2100	barA	0.6	Signal transduction histidine-protein kinase BarA
2102	2102	0.6	Hypothetical
2127	2127	0.6	Hypothetical
2165	rlmH	0.6	Ribosomal RNA large subunit methyltransferase H
2183	2183	0.6	Hypothetical
2186	2186	0.5	Hypothetical
2192	yxaH	0.6	Uncharacterized protein yxaH
2211	2211	0.6	Hypothetical
2280	2280	0.6	Hypothetical Protein 2280
2284	emrE	0.6	Multidrug transporter emrE
2295	2295	0.4	Hypothetical Protein 2295
2303	2303	0.6	Hypothetical
2327	2327	0.5	Hypothetical Protein 2327

2334	yjeS	0.4	Putative electron transport protein yjeS
2371	2371	0.4	Hypothetical
2387	2387	0.6	Acetyltransferase
2388	2388	0.5	Hypothetical Protein 2388
2465	2465	0.6	Hypothetical Protein 2465
2492	bcd	0.6	Acyl-CoA dehydrogenase, short-chain specific
2515	rplI	0.6	50S ribosomal protein L9
2516	insK	0.5	Putative transposase InsK for insertion sequence element IS150
2530	yafM	0	Hypothetical
2569	azoR	0.6	FMN-dependent NADH-azoreductase
2580	thiD	0.5	Hydroxymethylpyrimidine/phosphomethylpyrimidine kinase
2583	chrR	0.5	Transcriptional activator ChrR
2584	tcp	0.4	Methyl-accepting chemotaxis citrate transducer
2585	yddU	0.6	Uncharacterized protein y4IL
2590	2590	0.6	Hypothetical
2596	nemA	0.6	N-ethylmaleimide reductase
2620	dgkA	0.6	Diacylglycerol kinase
2643	2643	0.6	Hypothetical
2658	mutX	0.6	Mutator mutT protein
2683	yjgH	0.6	Endoribonuclease L-PSP
2684	2684	0.5	Hypothetical Protein 2684
2685	2685	0.5	Hypothetical
2689	2689	0.2	Hypothetical Protein 2689
2690	2690	0.6	Hypothetical Protein 2690
2878	fabG	0.5	3-oxoacyl-[acyl-carrier-protein] reductase FabG
2879	2879	0.6	Putative membrane protein SCO5905
2885	atpC	0.4	ATP synthase epsilon chain
2905	2905	0.5	Conserved Hypothetical Protein
2914	2914	0.4	Hypothetical Protein 2914
2915	2915	0.6	Hypothetical Protein 2915
2928	2928	0.5	Hypothetical Protein 2928
2971	2971	0.5	Hypothetical Protein 2971
2974	2974	0.4	Thioredoxin
2988	2988	0.6	Hypothetical
2990	hmuV	0.6	Hemin import ATP-binding protein HmuV
2993	yncE	0.5	Uncharacterized protein YncE
2994	besA	0.5	Ferri-bacillibactin esterase BesA
2995	2995	0.5	Hypothetical Protein 2995

3003	rcsB	0.5	Capsular synthesis regulator component B
3026	ycaQ	0.6	Uncharacterized protein ycaQ
3027	3027	0.6	Hypothetical Protein 3027
3028	3028	0.3	Hypothetical Protein 3028
3035	3035	0.5	Flagellar Basal Body P-Ring Biosynthesis Protein FlgA
3039	flgC	0.5	Flagellar basal-body rod protein flgC
3041	flgE	0.6	Flagellar hook protein flgE
3042	flgF	0.5	Flagellar basal-body rod protein flgF
3043	flgG	0.5	Flagellar basal-body rod protein flgG
3044	flgH	0.5	Flagellar L-ring protein
3058	nreC	0.6	Oxygen regulatory protein nreC
3059	rpoN	0.5	RNA polymerase sigma-54 factor
3060	cheB	0.6	Chemotaxis response regulator protein-glutamate methylesterase
3063	fliE	0.5	Flagellar hook-basal body complex protein FliE
3064	fliF	0.5	Flagellar M-ring protein
3065	fliG	0.6	Flagellar motor switch protein FliG
3066	3066	0.4	Flagellar Assembly Protein FliH
3067	fliI	0.5	Flagellum-specific ATP synthase
3070	fliL	0.6	Flagellar Basal Body-Associated Protein FliL
3071	fliM	0.5	Flagellar motor switch protein FliM
3072	fliN	0.5	Flagellar motor switch protein FliN
3082	flhB	0.5	Flagellar biosynthetic protein flhB
3083	flhA	0.6	Flagellar biosynthesis protein flhA
3084	flhF	0.5	Flagellar biosynthesis protein flhF
3131	3131	0.6	Hypothetical Protein 3131
3191	3191	0	Hypothetical
3193	3193	0.3	Methyltransferase
3212	kdsC	0.4	3-deoxy-D-manno-octulosonate 8-phosphate phosphatase KdsC
3213	3213	0.6	Hypothetical Protein 3213
3214	3214	0.6	Hypothetical Protein 3214
3216	3216	0.6	Hypothetical
3220	wcaJ	0.6	Putative colanic biosynthesis UDP-glucose lipid carrier transferase
3253	yafC	0.6	Uncharacterized HTH-type transcriptional regulator HI_1364
3259	der	0.6	GTPase Der
3273	3273	0.6	Hypothetical Protein 3273
3291	3291	0.6	Hypothetical Protein 3291
3302	pcaJ	0.5	3-oxoadipate CoA-transferase subunit B
3382	3382	0.6	Hypothetical Protein 3382

3388	3388	0.6	Hypothetical Protein 3388
3437	fdxA	0.5	Ferredoxin 1
3445	thiDE	0.6	Bifunctional protein thiED
3451	fpvA	0.5	Ferripyoverdine receptor
3452	3452	0.6	Hypothetical Protein 3452
3465	folP	0.6	Dihydropteroate synthase
3466	3466	0.6	Hypothetical Protein 3466
3488	p20	0.3	Uncharacterized N-acetyltransferase p20
3510	ctb	0.6	Group 3 truncated hemoglobin ctb
3511	3511	0.4	Hypothetical
3565	3565	0.6	Hypothetical
3567	3567	0.6	Phospholipase D
3586	3586	0.5	Hypothetical
3588	3588	0.6	Hypothetical Protein 3588
3598	3598	0.6	Uncharacterized protein HI_1419
3601	pupA	0.6	Ferric-pseudobactin 358 receptor
3619	3619	0.6	2OG-Fe(II) Oxygenase
3623	pstC	0.5	Phosphate transport system permease protein pstC
3646	yeaM	0.6	Uncharacterized HTH-type transcriptional regulator yeaM
3684	3684	0	Transposase
3685	insF1	0	Insertion element IS600 uncharacterized 31 kDa protein
3726	3726	0.6	Hypothetical
3741	czcD	0.6	Cation efflux system protein CzcD
3753	ycfJ	0.6	Uncharacterized protein ycfJ
3755	3755	0.6	Hypothetical Protein 3755
3763	3763	0.6	Conserved Hypothetical Protein
3783	ddh	0.6	2-hydroxyacid dehydrogenase homolog
3796	mviN	0.6	Virulence factor mviN homolog
3797	rpsT	0.6	30S ribosomal protein S20
3815	3815	0.5	Hypothetical
3821	3821	0.6	Hypothetical
3844	3844	0.6	Hypothetical Protein 3844
3845	fpvA	0.6	Ferripyoverdine receptor
3875	3875	0.6	TetR Family Transcriptional Regulator
3895	lktD	0.5	Leukotoxin secretion protein D
3932	3932	0.6	Hypothetical Protein 3932
3934	ybiX	0.5	PKHD-type hydroxylase Smlt1146
3959	3959	0.5	Hypothetical

3986	3986	0.2	Hypothetical
4001	4001	0.5	Hypothetical Protein 4001
4006	4006	0.5	Hypothetical Protein 4006
4021	4021	0.5	Hypothetical
4041	ycfF	0.5	HIT-like protein HI_0961
4165	4165	0.6	Hypothetical Protein 4165
4183	piv	0.4	Pilin gene-inverting protein
4243	4243	0.4	Hypothetical
4245	hpuB	0.4	Hemoglobin-haptoglobin utilization protein B
4246	4246	0.3	Hypothetical
4265	yfbP	0.6	Uncharacterized protein yfbP
4268	tonB	0.4	Protein tonB
4326	4326	0.6	Hypothetical Protein 4326
4352	dsbC	0.6	Thiol:disulfide interchange protein DsbC
4388	4388	0.6	Hypothetical
4410	4410	0.6	Hypothetical
4418	ytsP	0.6	Protein ytsP
4419	4419	0.6	TfoX Domain-Containing Protein
4468	yjbJ	0.6	Putative murein lytic transglycosylase yjbJ
4486	4486	0.6	CopY Family Transcriptional Regulator
4491	4491	0.5	Hypothetical Protein 4491
4492	4492	0.6	Hypothetical Protein 4492
4497	4497	0.5	Paar Motif Family Protein
4498	4498	0.6	Hypothetical
4503	4503	0.3	Hypothetical Protein 4503
4505	4505	0.6	Hypothetical Protein 4505
4507	4507	0.6	Transcriptional Factor
4511	4511	0.5	Hypothetical Protein 4511
4528	4528	0.4	Hypothetical
4529	4529	0.6	Hypothetical
4537	4537	0.5	Hypothetical Protein 4537
4540	vanX	0.5	D-alanyl-D-alanine dipeptidase
4565	4565	0.6	Hypothetical
4583	4583	0.4	Hypothetical
4597	kdpC	0.6	Potassium-transporting ATPase C chain
4604	radC	0.5	UPF0758 protein XCC3860
4626	4626	0.4	Xylose Isomerase Domain-Containing Protein
4627	ycdJ	0.3	Soluble epoxide hydrolase

4634	4634	0.5	Hypothetical
4653	4653	0.6	Hypothetical
4689	ohr	0.6	Organic hydroperoxide resistance protein
4719	4719	0.6	Hypothetical
4720	pdhA	0.5	Pyruvate dehydrogenase E1 component
4798	4798	0.5	Hypothetical
4809	rcsB	0.6	Capsular synthesis regulator component B
4831	4831	0.6	Glyoxalase/Bleomycin Resistance Protein/Dioxygenase
4861	yeaC	0.6	Uncharacterized protein yeaC
4869	4869	0.5	Hypothetical
4932	4932	0.6	Cysteine Dioxygenase Type I
4941	acoD	0.2	Acetaldehyde dehydrogenase 2
4953	4953	0.6	Hypothetical Protein 4953
4964	4964	0.6	Hypothetical Protein 4964
4978	4978	0.6	Hypothetical Protein 4978
4997	ycaN	0.6	Uncharacterized HTH-type transcriptional regulator ycaN
4998	4998	0.3	Hypothetical Protein 4998
5020	5020	0.3	Hypothetical
5021	slyA	0.4	Transcriptional regulator slyA
5026	yfcA	0.6	UPF0721 transmembrane protein ORF9
5027	ychJ	0.6	UPF0225 protein XCC4159
