# Supplementary material for: Root-microbe systems: the effect and mode of interaction of Stress Protecting Agent (SPA) Stenotrophomonas rhizophila DSM14405T
Source: Front Plant Sci. 2013 May 14;4:141. doi: 10.3389/fpls.2013.00141 (PMC3653106; doi:10.3389/fpls.2013.00141)
Supplement: Table S1A — Significantly up-regulated genes in S. rhizophila DSM14405T under salt shock. [file DataSheet1.ZIP › 51567_Berg_Table_S1A.PDF]

Supplementary Table 1a: significantly up-regulated genes in *S. rhizophila* DSM14405T under salt shock

locus tag	gene	fold change	product
1014	ytfN	2.3	Uncharacterized protein ytfN
1028	sopA	2.0	Uncharacterized protein PP_0002
1044	rpmB	2.0	50S ribosomal protein L28
1045	rpmG	1.8	50S ribosomal protein L33
1057	1057	2.0	Hypothetical Protein 1057
1058	1058	2.0	Hypothetical
1069	1069	1.6	Hypothetical
1072	acpP2	1.9	Acyl carrier protein 2
1098	dkgB	1.7	2,5-diketo-D-gluconic acid reductase B
1109	1109	2.9	Hypothetical
1116	yadG	1.9	Uncharacterized ABC transporter ATP-binding protein YadG
1138	aroE	1.7	Shikimate dehydrogenase
1157	ycgL	2.1	Uncharacterized protein ycgL
1158	mnmG	2.0	tRNA uridine 5-carboxymethylaminomethyl modification enzyme MnmG
1195	1195	1.6	Hypothetical Protein 1195
1228	speE	1.9	Spermidine synthase
1238	1238	1.6	Hypothetical
1239	kefA	2.3	Potassium efflux system KefA
1247	pdhC	6.7	Dihydrolipoyllysine-residue acetyltransferase component of pyruvate dehydrogenase complex
1248	1248	4.3	Hypothetical
1249	pdhB	4.4	Pyruvate dehydrogenase E1 component subunit beta
1250	pdhA	3.4	Pyruvate dehydrogenase E1 component subunit alpha
1257	hmgA	1.7	Homogentisate 1,2-dioxygenase
1262	1262	2.7	Hypothetical
1263	yrbG	1.8	Uncharacterized membrane protein MJ0091
1267	phaD	1.8	Probable K(+)/H(+) antiporter subunit D
1268	phaC	1.7	Probable K(+)/H(+) antiporter subunit C
1278	trpE	1.8	Anthranilate synthase component 1
1281	yggE	1.7	Hypothetical
1283	trpD	1.6	Anthranilate phosphoribosyltransferase
1284	trpC	2.3	Indole-3-glycerol phosphate synthase
1285	1285	2.0	Hypothetical
1288	speD	2.5	S-adenosylmethionine decarboxylase proenzyme
1289	coq7	1.9	2-nonaprenyl-3-methyl-6-methoxy-1,4-benzoquinol hydroxylase
1290	rplM	7.2	50S ribosomal protein L13

1291	rpsI	8.1	30S ribosomal protein S9
1293	1293	2.1	Bacterioferritin-Associated Ferredoxin
1294	bfr	5.9	Bacterioferritin
1295	gacS	1.9	Sensor protein gacS
1296	phoB	1.7	Uncharacterized 76.5 kDa protein in phbC 3'region
1298	1298	2.1	Hypothetical
1299	1299	1.6	Hypothetical
1302	1302	2.1	Signaling Modulator Of AmpD AmpE
1313	ygaU	4.4	Uncharacterized protein ygaU
1314	1314	3.3	Hypothetical
1315	icd	2.0	Isocitrate dehydrogenase [NADP]
1328	rho	1.6	Transcription termination factor Rho
1329	trxA	2.5	Thioredoxin-1
1330	rhlB	6.3	ATP-dependent RNA helicase rhlB
1331	ftsE	1.9	Cell division ATP-binding protein FtsE
1332	ftsX	2.0	Cell division protein ftsX
1335	1335	1.9	Hypothetical
1339	rpoH	3.0	RNA polymerase sigma-32 factor
1340	purD	1.9	Phosphoribosylamine--glycine ligase
1354	1354	4.5	Hypothetical
1355	1355	3.6	Hypothetical
1379	1379	1.6	Hypothetical
1380	1380	2.3	Hypothetical
1387	opdE	2.2	Transcription regulatory protein opdE
1388	ycjY	2.7	Uncharacterized protein PA2218
1389	ycjZ	9.1	Putative transcriptional regulator
1390	1390	13.6	Hypothetical
1391	1391	8.1	Hypothetical Protein 1391
1392	lytH	2.1	L-Ala--D-Glu endopeptidase
1393	1393	1.8	Hypothetical
1409	yetL	3.5	Uncharacterized HTH-type transcriptional regulator yetL
1410	yhaZ	3.8	Uncharacterized protein yhaZ
1411	1411	2.1	Hypothetical
1442	smg	1.8	Protein smg homolog
1445	def	2.1	Peptide deformylase
1446	fmt	2.1	Methionyl-tRNA formyltransferase
1447	rsmB	2.7	Ribosomal RNA small subunit methyltransferase B
1458	rpoD	2.0	RNA polymerase sigma factor rpoD

1483	yoeA	1.7	Probable multidrug resistance protein yoeA
1484	1484	3.0	Hypothetical
1489	1489	1.8	Hypothetical Protein 1489
1499	yigF	2.0	Uncharacterized protein yigF
1511	glpK	2.1	Glycerol kinase
1512	glpD	2.4	Glycerol-3-phosphate dehydrogenase
1513	ompW	3.6	Outer membrane protein W
1515	aceF	3.0	Dihydrolipoyllysine-residue acetyltransferase component of pyruvate dehydrogenase complex
1516	1516	3.2	Hypothetical
1517	lpdA	2.0	Dihydrolipoyl dehydrogenase
1518	1518	4.1	Hypothetical
1519	1519	1.7	Hypothetical Protein 1519
1523	atpH	2.1	ATP synthase subunit delta
1526	atpD	1.9	ATP synthase subunit beta
1527	atpC	3.0	ATP synthase epsilon chain
1531	glmU	2.1	Bifunctional protein glmU
1554	1554	1.6	Fis Family Transcriptional Regulator
1557	metN	3.4	Methionine import ATP-binding protein MetN
1558	metI	2.2	Methionine import system permease protein metI
1579	1579	2.1	Hypothetical
1580	1580	3.5	Hypothetical
1596	mltB	1.9	Membrane-bound lytic murein transglycosylase B
1598	dacC	1.7	D-alanyl-D-alanine carboxypeptidase dacC
1601	lipB	2.3	Octanoyltransferase
1602	lipA	2.1	Lipoyl synthase
1603	prc	2.4	Tail-specific protease
1604	1604	7.8	Hypothetical
1605	1605	3.3	Hypothetical
1606	yqeC	2.9	Putative 6-phosphogluconate dehydrogenase YqeC
1607	ydgJ	2.6	Uncharacterized oxidoreductase ydgJ
1608	osmY	4.3	Secreted Protein
1609	bphB	2.5	Cyanobacterial phytochrome B
1611	zraS	1.8	Sensor protein zraS
1634	1634	1.5	3-Demethylubiquinone-9 3-Methyltransferase
1635	1635	1.5	LuxR Family Transcriptional Regulator
1638	betA	2.3	Choline dehydrogenase
1639	betB	2.2	Betaine aldehyde dehydrogenase
1644	cc4	1.8	Cytochrome c4

1650	adhA	1.6	Probable formaldehyde dehydrogenase AdhA
1663	1663	2.3	Hypothetical
1674	arpC	2.0	Antibiotic efflux pump outer membrane protein ArpC
1675	1675	2.7	Hypothetical
1683	ydhD	2.3	Uncharacterized monothiol glutaredoxin ycf64-like
1701	oprP	1.7	Porin P
1703	maeB	2.4	NADP-dependent malic enzyme
1714	tolC	1.7	Outer membrane protein tolC
1715	pcm3	1.8	Protein-L-isoaspartate O-methyltransferase 3
1720	gdhA	1.9	NADP-specific glutamate dehydrogenase
1731	opuD	6.6	Glycine betaine transporter OpuD
1763	1763	1.6	Hypothetical
1766	yiiG	4.1	Uncharacterized protein yiiG
1767	1767	1.6	Putative potassium channel protein RPA4233
1768	argD	2.0	Acetylornithine aminotransferase
1781	ygfB	1.9	UPF0149 protein XOO1028
1782	pepP	1.7	Xaa-Pro aminopeptidase
1790	yicC	2.2	UPF0701 protein yicC
1792	rpoZ	4.8	DNA-directed RNA polymerase subunit omega
1794	yjgF	1.5	RutC family protein in vnfA 5'region
1795	recG	1.7	ATP-dependent DNA helicase recG
1798	rpmE2	6.4	50S ribosomal protein L31 type B
1799	gltA	3.4	Citrate synthase
1819	1819	1.8	Hypothetical Protein 1819
1820	1820	1.7	Uncharacterized protein BB_0173
1823	gltT	1.7	Proton/sodium-glutamate symport protein
1831	blh	3.1	Beta-lactamase hydrolase-like protein
1836	gapA	1.6	Glyceraldehyde-3-phosphate dehydrogenase
1839	pgk	2.0	Phosphoglycerate kinase
1840	gph	2.2	5'-nucleotidase
1842	1842	1.9	Probable fructose-bisphosphate aldolase class 1
1846	ymdB	4.9	Macro domain-containing protein XCC3184
1860	1860	3.8	Hypothetical
1882	sucC	2.3	Succinyl-CoA ligase [ADP-forming] subunit beta
1883	sucD	2.8	Succinyl-CoA ligase [ADP-forming] subunit alpha
1884	nadE	1.7	Probable glutamine-dependent NAD(+) synthetase
1890	yuxK	1.6	Uncharacterized protein yuxK
1892	otsA	4.8	Alpha,alpha-trehalose-phosphate synthase [UDP-forming]

1893	1893	5.1	Glycoside Hydrolase
1894	otsB	9.4	Trehalose-phosphate phosphatase
1900	poxB	5.0	Pyruvate dehydrogenase [ubiquinone]
1914	yheS	3.1	Uncharacterized ABC transporter ATP-binding protein YheS
1915	ampR	3.8	HTH-type transcriptional activator AmpR
1916	1916	6.3	Beta-lactamase L2
1917	ggpS	8.3	Glucosylglycerol-phosphate synthase
1918	ycaD	7.7	Uncharacterized MFS-type transporter CKO_02171
1919	1919	2.9	Hypothetical
1921	aspS	2.6	Aspartyl-tRNA synthetase
1924	1924	2.8	Hypothetical
1925	yebC	3.0	UPF0082 protein Smal_3128
1926	ruvC	1.7	Crossover junction endodeoxyribonuclease ruvC
1935	tolB	4.8	Protein tolB
1936	pal	2.8	Peptidoglycan-associated lipoprotein
1937	ybgF	1.9	Uncharacterized protein PP_1224
1938	queE	2.1	Probable 7-carboxy-7-deazaguanine synthase
1940	1940	1.7	Hypothetical
1941	1941	2.7	Hypothetical
1942	1942	1.5	Hypothetical Protein 1942
1943	1943	1.5	Hypothetical Protein 1943
1944	1944	1.8	Hypothetical
1945	1945	1.9	Hypothetical Protein 1945
1946	galE	1.6	NAD-Dependent Epimerase/Dehydratase
1961	dps	3.1	Uncharacterized protein slr1894
1962	hrpA	2.4	ATP-dependent RNA helicase hrpA
1964	relA	1.6	GTP pyrophosphokinase
1965	1965	2.1	Hypothetical
1980	rsmE	1.9	Ribosomal RNA small subunit methyltransferase E
1981	bioA	1.7	Adenosylmethionine-8-amino-7-oxononanoate aminotransferase
1992	flp	3.4	Protein flp
1997	fadE	2.9	Acyl-coenzyme A dehydrogenase
2000	btuB	1.6	Vitamin B12 transporter BtuB
2001	2001	2.9	Hypothetical
2006	yhjG	2.3	Uncharacterized protein yhjG
2023	met2	1.7	Homoserine O-acetyltransferase
2039	aroB	2.1	3-dehydroquinate synthase
2040	aroK	1.8	Shikimate kinase

2041	2041	2.7	Hypothetical
2042	2042	3.4	Hypothetical
2043	pdxH	1.8	Pyridoxine/pyridoxamine 5'-phosphate oxidase
2046	prpR	1.5	Propionate catabolism operon regulatory protein
2047	prpB	2.2	Methylisocitrate lyase
2049	acnA	3.2	Aconitate hydratase
2050	2050	3.3	Hypothetical
2051	mii	2.6	3-methylitaconate isomerase
2052	prpD	2.8	2-methylcitrate dehydratase
2054	pbpC	1.6	Penicillin-binding protein 1C
2055	bfr	4.1	Ferritin Dps Family Protein
2056	2056	2.1	Putative peroxiredoxin sll1621
2061	hflC	2.1	Protein HflC
2086	yhhW	1.7	Putative quercetin 2,3-dioxygenase PA3240
2090	fadJ	3.9	Fatty acid oxidation complex subunit alpha
2092	2092	3.1	Negative Regulator Of Sigma E Activity
2093	degP	2.1	Probable serine protease do-like
2094	lepA	2.1	Elongation factor 4
2098	era	2.2	GTPase Era
2099	recO	1.5	DNA repair protein recO
2100	barA	3.1	Signal transduction histidine-protein kinase BarA
2105	nagZ	1.8	Beta-hexosaminidase
2106	hpt	1.5	Hypoxanthine-guanine phosphoribosyltransferase
2110	2110	5.5	Hypothetical
2111	2111	13.5	Metallophosphoesterase
2112	lhr	2.2	Uncharacterized ATP-dependent helicase MJ0294
2116	2116	5.4	Hypothetical Protein 2116
2117	2117	1.6	Hypothetical Protein 2117
2120	ldh	1.6	Leucine dehydrogenase
2125	yaiL	1.7	Uncharacterized protein yaiL
2130	sigW	3.7	RNA polymerase sigma factor sigW
2131	2131	3.5	Hypothetical
2132	2132	4.4	Hypothetical
2133	2133	3.9	Hypothetical Protein 2133
2134	2134	2.0	Hypothetical
2136	phnA	2.1	Protein phnA
2156	cph2	1.7	Phytochrome-like protein cph2
2157	2157	3.4	Hypothetical

2160	leuS	1.9	Leucyl-tRNA synthetase
2161	rlpB	1.7	Rare Lipoprotein B
2162	holA	2.3	DNA polymerase III subunit delta
2164	ybeB	3.0	Uncharacterized protein ybeB
2171	yhdP	2.3	Uncharacterized protein yhdP
2172	tldD	2.4	Protein tldD
2173	yjgA	2.7	UPF0307 protein Smal_2897
2174	pmbA	1.9	Protein pmbA
2175	2175	1.6	Hypothetical
2205	mrp	2.3	Protein mrp homolog
2222	2222	1.5	Hypothetical
2225	trpF	2.1	N-(5'-phosphoribosyl)anthranilate isomerase
2235	accD	2.0	Acetyl-coenzyme A carboxylase carboxyl transferase subunit beta
2236	glmM	2.6	Phosphoglucosamine mutase
2241	tpiA	1.6	Triosephosphate isomerase
2242	secG	4.9	Protein-export membrane protein secG
2243	nuoA	4.4	NADH-quinone oxidoreductase subunit A
2244	nuoB	3.0	NADH-quinone oxidoreductase subunit B
2245	nuoC	3.2	NADH-quinone oxidoreductase subunit C
2246	nuoD2	1.6	NADH-quinone oxidoreductase subunit D 2
2248	nuoF	1.9	NADH-quinone oxidoreductase subunit F
2249	nuoG	3.8	NADH-quinone oxidoreductase subunit G
2256	nuoN	2.1	NADH-quinone oxidoreductase subunit N
2257	rimP	1.8	Ribosome maturation factor rimP
2258	nusA	4.1	Transcription elongation protein nusA
2259	infB	6.3	Translation initiation factor IF-2
2260	rbfA	2.1	Ribosome-binding factor A
2262	rpsO	5.0	30S ribosomal protein S15
2263	pnp	6.1	Polyribonucleotide nucleotidyltransferase
2273	infC	4.3	Translation initiation factor IF-3
2274	rpmI	2.9	50S ribosomal protein L35
2275	rplT	3.2	50S ribosomal protein L20
2281	2281	3.7	Hypothetical
2282	2282	3.6	Hypothetical
2283	betI	2.1	TetR Family Transcriptional Regulator
2284	emrE	1.5	Multidrug transporter emrE
2292	2292	2.8	UPF0391 membrane protein XCC1302
2300	2300	6.4	Hypothetical Protein 2300

2301	slyA	5.8	MarR Family Transcriptional Regulator
2305	dxs	2.9	1-deoxy-D-xylulose-5-phosphate synthase
2322	2322	3.1	Hypothetical Protein 2322
2331	pkn1	2.1	Serine/threonine-protein kinase pkn1
2332	yfgC	1.8	TPR repeat-containing protein yfgC
2335	yjeF	2.2	Uncharacterized protein yjeF
2336	yjeE	1.7	UPF0079 ATP-binding protein yjeE
2337	amiC	2.0	N-acetylmuramoyl-L-alanine amidase AmiC
2347	suhB	3.6	Inositol-1-monophosphatase
2351	yjeK	2.1	Uncharacterized KamA family protein YjeK
2352	efp	2.0	Elongation factor P
2355	gph	2.0	Phosphoglycolate phosphatase
2356	2356	1.6	Hypothetical
2357	yebA	3.2	Uncharacterized metalloprotease BUsg_310
2358	pyrC	4.7	Dihydroorotase
2362	cysS	2.5	Cysteinyl-tRNA synthetase
2364	argF'	1.6	N-acetylornithine carbamoyltransferase
2365	argG	2.2	Argininosuccinate synthase
2366	argE	4.6	Acetylornithine deacetylase
2367	argB	3.9	Acetylglutamate kinase
2368	2368	4.6	GCN5-Related N-Acetyltransferase
2369	argC	4.7	N-acetyl-gamma-glutamyl-phosphate reductase
2370	argH	5.6	Argininosuccinate lyase
2371	2371	6.8	Hypothetical
2372	proB	5.8	Glutamate 5-kinase
2373	proA	4.5	Gamma-glutamyl phosphate reductase
2404	rluB	7.3	Ribosomal large subunit pseudouridine synthase B
2405	scpB	1.7	Segregation and condensation protein B homolog
2406	scpA	3.1	Segregation and condensation protein A
2408	bolA	1.9	Uncharacterized protein in cobS 5'region
2409	ccpA	4.1	Glucose-resistance amylase regulator
2410	2410	2.1	NB-Dependent Receptor Plug
2414	araQ	2.4	L-arabinose transport system permease protein AraQ
2415	2415	3.6	Hypothetical
2416	2416	1.6	Endonuclease/Exonuclease/Phosphatase
2418	rne	5.1	Ribonuclease E
2429	sodA	4.4	Superoxide dismutase [Mn]
2430	2430	7.4	Endonuclease/Exonuclease/Phosphatase Family Protein

2442	fpr	2.0	Ferredoxin--NADP reductase
2449	2449	2.5	Hypothetical Protein 2449
2450	arsC	1.6	Arsenate reductase
2453	cspA	1.8	Major cold shock protein CspA
2454	2454	2.5	Peptidoglycan-Associated Outer Membrane Lipoprotein
2455	2455	2.1	Hypothetical
2457	yjjV	1.6	Uncharacterized deoxyribonuclease yjjV
2464	dnaB	2.1	Replicative DNA helicase
2467	lpdG	4.1	Dihydrolipoamide dehydrogenase
2468	sucB	3.0	Dihydrolipoyllysine-residue succinyltransferase component of 2-oxoglutarate dehydrogenase complex
2469	sucA	2.1	2-oxoglutarate dehydrogenase E1 component
2483	fkpA	2.2	Probable FKBP-type peptidyl-prolyl cis-trans isomerase fkpA
2484	udg	2.1	UDP-glucose 6-dehydrogenase
2486	yaiL	2.2	Hypothetical
2497	gdhB	1.7	NAD-specific glutamate dehydrogenase
2513	rpsF	2.8	30S ribosomal protein S6
2514	rpsR	1.9	30S ribosomal protein S18
2515	rplI	1.6	50S ribosomal protein L9
2519	zipA	1.8	Cell division protein ZipA homolog
2521	ligA	2.0	DNA ligase
2522	phnO	1.6	Acetyltransferase Gnat Family
2525	mtnA	2.2	Methylthioribose-1-phosphate isomerase
2526	gyrA	1.9	DNA gyrase subunit A
2553	hutH	1.7	Histidine ammonia-lyase
2555	mtaD	1.5	5-methylthioadenosine/S-adenosylhomocysteine deaminase
2556	hutC	1.7	Histidine utilization repressor
2557	2557	2.1	Hypothetical Protein 2557
2561	2561	1.7	Hypothetical
2565	tonB	1.8	Protein tonB
2567	serS	1.6	Seryl-tRNA synthetase
2588	foxA	2.1	Ferrioxamine B receptor
2603	2603	2.1	Hypothetical Protein 2603
2628	2628	4.5	Hypothetical
2629	2629	5.3	Hypothetical Protein 2629
2638	2638	2.0	Response Regulator Receiver Protein
2641	lhr	2.0	Probable ATP-dependent helicase lhr
2653	2653	3.0	Hypothetical
2655	ppsA	3.1	Phosphoenolpyruvate synthase

2661	2661	2.0	Uncharacterized protein Alvin_0062
2664	2664	2.8	PspC Domain Protein
2665	2665	2.2	Hypothetical
2666	2666	2.6	Hypothetical
2667	yhgF	1.9	Protein yhgF
2671	2671	1.9	Two-Component System Regulatory Protein
2675	zntR	3.5	HTH-type transcriptional regulator zntR
2678	dsbG	3.0	Thiol:disulfide interchange protein DsbG
2679	2679	2.9	Hypothetical
2680	adiC	1.9	Arginine/agmatine antiporter
2686	map	1.9	Methionine aminopeptidase
2716	bglB	2.8	Thermostable beta-glucosidase B
2725	sapB	1.6	Protein SapB
2731	2731	2.1	Conserved Hypothetical Protein
2733	2733	2.3	Hypothetical Protein 2733
2734	2734	3.3	Hypothetical
2735	mltD	3.2	Muramidase-2
2736	2736	3.6	Hypothetical Protein 2736
2737	2737	3.6	Rhs Element Vgr Protein
2741	2741	1.8	Rhs Element Vgr Protein
2742	2742	7.4	Hypothetical
2743	2743	6.2	Hypothetical
2744	2744	2.7	Hypothetical
2745	2745	2.3	Hypothetical Protein 2745
2746	2746	4.5	Lipoprotein
2747	2747	3.5	Hypothetical
2748	2748	2.6	Hypothetical
2749	2749	2.6	Type VI secretiom system effector, Hcp1 family
2750	2750	2.1	Hypothetical
2751	2751	7.4	Hypothetical
2752	2752	8.4	Hypothetical
2753	clpV1	9.3	Protein ClpV1
2754	2754	7.8	Conserved Hypothetical Protein
2755	2755	7.2	Rhs Element Vgr Protein
2756	2756	4.2	Hypothetical
2761	2761	3.4	Rhs Element Vgr Protein
2774	2774	4.0	Conserved Hypothetical Protein
2776	2776	4.4	Hypothetical

2777	2777	7.3	Hypothetical
2778	icmF	5.0	Type VI secretion system protein IcmF
2779	2779	7.3	Hypothetical
2780	yiaD	5.6	Uncharacterized protein Rv0899/MT0922
2781	2781	2.4	Type VI secretion system-associated protein, ImpA family
2785	2785	8.5	Rhs Element Vgr Protein
2786	2786	10.8	Hypothetical
2787	2787	6.7	Hypothetical
2788	2788	7.0	Hypothetical
2789	2789	4.6	Hypothetical
2790	2790	2.1	Hypothetical
2791	2791	6.8	Rhs Element Vgr Protein
2792	2792	6.8	Hypothetical
2793	2793	2.8	Hypothetical Protein 2793
2794	2794	3.8	Hypothetical
2813	2813	1.9	Flp Pilus Assembly Protein CpaB
2829	2829	2.6	Hypothetical
2830	yliI	3.0	L-sorbosone dehydrogenase
2849	mntH	3.6	Probable manganese transport protein mntH
2864	2864	2.8	Hypothetical
2865	qor	4.6	Quinone oxidoreductase
2866	ycjZ	2.8	Putative transcriptional regulator
2869	yhfK	3.1	Uncharacterized sugar epimerase yhfK
2870	2870	3.4	Hypothetical
2886	tetA	3.8	Tetracycline resistance protein, class A
2887	dhbC	6.3	Isochorismate synthase dhbC
2888	dhbE	4.6	2,3-dihydroxybenzoate-AMP ligase
2889	dhbB	4.9	Isochorismatase
2890	entB	4.6	Phosphopantetheine Attachment Site Protein
2891	entF	3.9	Enterobactin synthase component F
2892	dhbA	2.6	2,3-dihydro-2,3-dihydroxybenzoate dehydrogenase
2894	nodW	1.6	Nodulation protein W
2895	nodV	2.8	Nodulation protein V
2900	yiaD	1.9	Hypothetical
2901	2901	1.9	Hypothetical
2902	2902	1.8	Hypothetical
2919	alsS	2.1	Acetolactate synthase
2920	gabD	1.6	Putative aldehyde-dehydrogenase-like protein y4uC

2956	2956	1.6	Hypothetical
2957	2957	1.6	Hypothetical
2969	2969	3.9	Hypothetical
2970	lcnDR2	15.3	Lacticin 481/lactococcin biosynthesis protein lcnDR2
2971	2971	5.1	Hypothetical Protein 2971
2972	fsr	1.7	Fosmidomycin resistance protein
2973	2973	7.5	Hypothetical
2974	2974	8.0	Thioredoxin
2975	2975	5.6	Host Attachment Protein
2977	2977	1.5	Hypothetical
2980	ousA	4.2	Osmoprotectant uptake system A
2990	hmuV	4.4	Hemin import ATP-binding protein HmuV
2991	hmuU	4.8	Hemin transport system permease protein hmuU
2992	hmuT	3.7	Hemin-binding periplasmic protein hmuT
2993	yncE	3.1	Uncharacterized protein YncE
2994	besA	4.4	Ferri-bacillibactin esterase BesA
2995	2995	1.7	Hypothetical Protein 2995
3006	crcB	1.9	Protein CrcB homolog
3007	rarA	3.1	Replication-associated recombination protein A
3010	ftsK	1.6	DNA translocase ftsK
3011	trxB	2.0	Thioredoxin reductase
3015	infA	2.5	Translation initiation factor IF-1
3017	clpA	2.2	ATP-dependent Clp protease ATP-binding subunit ClpA
3018	str	1.5	Streptomycin 3''-kinase
3023	hflD	1.7	High frequency lysogenization protein hflD homolog
3033	3033	2.1	Hypothetical
3034	3034	2.8	Negative Regulator Of Flagellin Synthesis Protein
3059	rpoN	2.2	RNA polymerase sigma-54 factor
3060	cheB	2.0	Chemotaxis response regulator protein-glutamate methylesterase
3061	hyfR	3.2	Hydrogenase-4 transcriptional activator
3106	acnB	3.4	Aconitate hydratase 2
3107	3107	1.9	Hypothetical
3109	rpfA	7.3	Aconitate hydratase
3110	rpfB	3.8	Long-chain-fatty-acid--CoA ligase
3113	rpfC	2.5	Sensory/regulatory protein RpfC
3114	rpfG	1.9	Cyclic di-GMP phosphodiesterase response regulator RpfG
3116	prfB	2.4	Peptide chain release factor 2
3117	yggG	1.6	Uncharacterized metalloprotease yggG

3124	3124	1.7	Hypothetical
3125	greA	2.5	Transcription elongation factor greA
3126	carB	1.6	Carbamoyl-phosphate synthase large chain
3127	carA	1.5	Carbamoyl-phosphate synthase small chain
3138	fabG2	2.2	Uncharacterized oxidoreductase Rv1350/MT1393
3139	yeiP	2.3	Elongation factor P-like protein
3140	3140	7.4	Hypothetical
3141	3141	6.4	Hypothetical
3145	resD	1.7	Transcriptional regulatory protein resD
3170	hisI	4.6	Histidine biosynthesis bifunctional protein HisIE
3171	hisF	6.0	Imidazole glycerol phosphate synthase subunit hisF
3172	hisA	5.8	1-(5-phosphoribosyl)-5-[(5- phosphoribosylamino)methylideneamino] imidazole-4-carboxamide isomerase
3173	hisH	6.3	Imidazole glycerol phosphate synthase subunit hisH
3174	hisB	4.1	Histidine biosynthesis bifunctional protein hisB
3175	hisC	6.6	Histidinol-phosphate aminotransferase
3176	hisD	4.5	Histidinol dehydrogenase
3177	hisG	2.5	ATP phosphoribosyltransferase
3180	hisS	2.1	Histidyl-tRNA synthetase
3181	fixK	2.2	Nitrogen fixation regulation protein fixK
3188	3188	2.7	GCN5-Like N-Acetyltransferase
3189	3189	2.6	Hypothetical
3190	3190	7.7	Hypothetical
3198	3198	2.6	Hypothetical Protein 3198
3201	3201	2.1	Hypothetical Protein 3201
3206	3206	2.5	Hypothetical Protein 3206
3208	ymcC	1.8	Lipoprotein
3209	yjbG	2.0	Uncharacterized protein yjbG
3221	betB	3.2	Betaine aldehyde dehydrogenase
3222	fabG	2.4	3-oxoacyl-[acyl-carrier-protein] reductase FabG
3224	3224	2.1	Uncharacterized methyltransferase Rv3342/MT3445
3225	pyrD	2.0	Dihydroorotate dehydrogenase (quinone)
3255	guaB	2.7	Inosine-5'-monophosphate dehydrogenase
3260	yfgL	2.2	Lipoprotein yfgL
3261	yfgM	3.1	UPF0070 protein yfgM
3262	3262	1.6	Hypothetical
3263	3263	1.7	Type IV Pilus Biogenesis/Stability Protein PilW
3268	fadA	2.1	3-ketoacyl-CoA thiolase
3269	gtaB	8.4	UTP--glucose-1-phosphate uridylyltransferase

3270	capD	2.9	Capsular polysaccharide biosynthesis protein CapD
3275	rpsA	3.5	30S ribosomal protein S1
3276	cmk	2.5	Cytidylate kinase
3277	rpmJ2	5.3	50S ribosomal protein L36 2
3280	3280	1.8	N-carbamoyl-D-amino acid hydrolase
3281	pat	1.5	Phosphinothricin N-acetyltransferase
3282	3282	1.5	Uncharacterized protein MJ1365
3287	3287	1.7	Cytochrome c-554(548)
3294	quiA	1.5	Quinate/shikimate dehydrogenase (quinone)
3295	pcaR	1.7	Pca regulon regulatory protein
3310	cspA	4.3	Major cold shock protein CspA
3323	secF	1.6	Protein translocase subunit SecF
3324	secD	1.5	Protein translocase subunit SecD
3325	yajC	2.2	UPF0092 membrane protein yajC
3328	argD	2.6	Acetylornithine/succinyldiaminopimelate aminotransferase
3329	3329	1.6	LOG family protein PA4923
3335	3335	2.8	Hypothetical
3336	tyrA	2.9	Probable arogenate/prephenate dehydrogenase
3338	dnaJ	3.1	Chaperone protein DnaJ
3343	recN	1.8	DNA repair protein recN
3349	yfjF	2.0	UPF0125 protein PD_1376
3378	yiaD	1.7	Inner membrane lipoprotein yiaD
3396	ytnP	2.4	Uncharacterized protein ytnP
3404	ftsY	2.2	Cell division protein ftsY
3412	sdhB	2.7	Succinate dehydrogenase iron-sulfur subunit
3413	sdhA	9.6	Succinate dehydrogenase flavoprotein subunit
3414	sdhD	7.0	Succinate dehydrogenase hydrophobic membrane anchor subunit
3415	sdhC	6.3	Succinate dehydrogenase cytochrome b556 subunit
3416	3416	5.1	Hypothetical
3417	ygfZ	1.6	tRNA-modifying protein ygfZ
3418	ugpC	2.8	sn-glycerol-3-phosphate import ATP-binding protein UgpC
3421	pgl	1.9	6-phosphogluconolactonase
3422	edd	2.5	Phosphogluconate dehydratase
3423	eda	2.2	2-dehydro-3-deoxy-phosphogluconate aldolase
3429	pgi	2.5	Glucose-6-phosphate isomerase
3430	panD	2.0	Aspartate 1-decarboxylase
3432	panB	2.0	3-methyl-2-oxobutanoate hydroxymethyltransferase
3433	folK	1.9	2-amino-4-hydroxy-6- hydroxymethyldihydropteridine pyrophosphokinase

3434	pcnB	2.5	Poly(A) polymerase
3437	fdxA	1.7	Ferredoxin 1
3438	3438	2.3	Hypothetical
3439	dapA	2.4	Dihydrodipicolinate synthase
3440	gcvR	1.7	Glycine cleavage system transcriptional repressor
3450	pld1	2.8	Pyridoxal 4-dehydrogenase
3452	3452	4.2	Hypothetical Protein 3452
3455	csrA	2.8	Carbon storage regulator homolog
3456	alaS	4.8	Alanyl-tRNA synthetase
3457	recX	4.1	Regulatory protein recX
3458	recA	3.9	Protein RecA
3459	lexA	6.6	LexA repressor
3460	ubiB	3.3	Probable ubiquinone biosynthesis protein UbiB
3462	hflX	4.0	GTP-binding protein hflX
3463	hfq	2.2	Protein hfq
3465	folP	1.6	Dihydropteroate synthase
3467	ftsH	1.9	ATP-dependent zinc metalloprotease FtsH
3469	yhbY	2.7	RNA-binding protein HI_1333
3470	3470	2.7	Hypothetical
3471	ygeR	2.0	Lipoprotein NlpD/LppB homolog
3473	pcm	2.2	Protein-L-isoaspartate O-methyltransferase
3474	surE	2.0	5'-nucleotidase surE
3481	eno	1.7	Enolase
3485	3485	2.0	Hypothetical Protein 3485
3495	yceA	1.9	UPF0176 protein XCC2086
3521	uvrC	1.7	UvrABC system protein C
3522	3522	2.7	NHL Repeat Containing Protein
3526	exbD	1.8	Biopolymer Transport Protein ExbD/lR
3542	purK	2.0	N5-carboxyaminoimidazole ribonucleotide synthase
3543	purE	1.9	N5-carboxyaminoimidazole ribonucleotide mutase
3544	3544	2.0	Hypothetical
3545	nadC	1.7	Nicotinate-nucleotide pyrophosphorylase [carboxylating]
3546	3546	2.0	Hypothetical
3547	3547	1.9	Hypothetical
3576	sad	2.3	Succinate semialdehyde dehydrogenase [NAD(P)+] Sad
3578	potH	2.0	Putrescine transport system permease protein PotH
3579	potG	3.5	Putrescine transport ATP-binding protein PotG
3582	puuD	2.1	Gamma-glutamyl-gamma-aminobutyrate hydrolase

3595	3595	1.6	Hypothetical
3596	yjiN	2.4	Uncharacterized protein yjiN
3599	3599	1.6	Uncharacterized protein HI_1420
3601	pupA	2.0	Ferric-pseudobactin 358 receptor
3611	mip	3.0	Outer membrane protein MIP
3638	rlmB	2.1	23S rRNA (guanosine-2'-O-)-methyltransferase RlmB
3639	3639	2.1	Hypothetical
3640	rnr	4.3	Ribonuclease R
3641	3641	1.7	Glutathione S-Transferase
3654	dapE	2.5	Succinyl-diaminopimelate desuccinylase
3655	yffB	2.4	Protein YffB
3656	dapD	2.3	2,3,4,5-tetrahydropyridine-2,6-dicarboxylate N-succinyltransferase
3657	glnD	2.6	[Protein-PII] uridylyltransferase
3659	3659	1.7	Protein U
3660	3660	2.3	Pili Assembly Chaperone
3661	yraJ	1.7	Uncharacterized outer membrane usher protein yraJ
3665	rpsB	4.0	30S ribosomal protein S2
3666	tsf	2.9	Elongation factor Ts
3670	frr	1.8	Ribosome-recycling factor
3674	yaeL	1.9	Putative zinc metalloprotease XF_1047
3676	lpxD	1.6	UDP-3-O-[3-hydroxymyristoyl] glucosamine N-acyltransferase
3678	lpxA	2.1	Acyl-[acyl-carrier-protein]--UDP-N- acetylglucosamine O-acyltransferase
3679	lpxB	2.4	Lipid-A-disaccharide synthase
3680	rnhB	1.8	Ribonuclease HII
3683	accA	1.7	Acetyl-coenzyme A carboxylase carboxyl transferase subunit alpha
3694	3694	2.3	Hypothetical
3708	deaD	8.4	Cold-shock DEAD box protein A homolog
3711	3711	1.8	Hypothetical
3717	3717	1.9	Hypothetical Protein 3717
3727	zur	1.8	Zinc uptake regulation protein
3728	gltX	2.0	Glutamyl-tRNA synthetase
3735	yfiQ	2.8	Uncharacterized protein yfiQ
3736	mfd	3.8	Transcription-repair-coupling factor
3737	3737	1.7	Hypothetical
3744	desA3	1.7	Stearoyl-CoA 9-desaturase
3745	paaE	2.7	Stearoyl-CoA 9-desaturase electron transfer partner
3748	3748	2.8	Outer Membrane Efflux Protein
3749	yjcR	2.9	36 kDa antigen

3750	ybhS	3.3	Hypothetical
3751	ybhR	2.6	Hypothetical
3753	ycfJ	5.7	Uncharacterized protein ycfJ
3764	rplS	3.5	50S ribosomal protein L19
3767	rpsP	5.4	30S ribosomal protein S16
3774	3774	1.9	Hypothetical
3776	radA	1.8	DNA repair protein RadA homolog
3777	yciR	1.6	Uncharacterized signaling protein PA1727
3786	3786	1.7	Hypothetical Protein 3786
3789	treA	2.4	Periplasmic trehalase
3794	ileS	1.6	Isoleucyl-tRNA synthetase
3797	rpsT	4.1	30S ribosomal protein S20
3798	obg	1.5	GTPase obg
3799	rpmA	5.3	50S ribosomal protein L27
3800	rplU	4.6	50S ribosomal protein L21
3805	echA17	1.7	Probable enoyl-CoA hydratase echA17
3806	tesB	1.8	Acyl-CoA thioesterase 2
3807	3807	1.7	Hypothetical
3814	3814	3.5	Hypothetical
3815	3815	3.3	Hypothetical
3816	3816	3.3	Hypothetical
3817	3817	5.3	Hypothetical
3818	3818	4.4	Hypothetical
3819	yodJ	2.5	Putative carboxypeptidase yodJ
3825	3825	1.9	GCN5-Related N-Acetyltransferase
3826	minC	1.7	Probable septum site-determining protein minC
3836	yggA	6.8	Putative amino-acid transporter Rv1986/MT2040
3882	cytR	2.2	HTH-type transcriptional repressor CytR
3885	ydgJ	1.6	Uncharacterized oxidoreductase y4hM
3902	kaiC	3.3	Circadian clock protein kinase kaiC
3910	ispB	2.3	Octaprenyl-diphosphate synthase
3913	murD	2.4	UDP-N-acetylmuramoylalanine--D-glutamate ligase
3914	3914	2.6	Hypothetical
3915	lysA	3.6	Diaminopimelate decarboxylase
3916	yddE	3.5	Uncharacterized isomerase mll1393
3917	osmC	3.1	Peroxiredoxin osmC
3920	3920	2.2	Hypothetical
3921	3921	2.6	Hypothetical

3922	yraA	3.8	Putative cysteine protease yraA
3924	aau3	2.9	Protein aau3
3925	sufB	1.6	UPF0051 protein slr0074
3926	sufC	3.6	Probable ATP-dependent transporter SufC
3927	sufD	8.3	FeS cluster assembly protein sufD
3928	csd	3.9	Probable cysteine desulfurase
3946	3946	1.8	Peptidase
3953	purM	2.2	Phosphoribosylformylglycinamidine cyclo-ligase
3957	3957	1.7	Hypothetical
3963	kdsC	2.0	3-deoxy-D-manno-octulosonate 8-phosphate phosphatase KdsC
3964	3964	1.8	Hypothetical
4000	4000	2.2	Glutathione-Dependent Formaldehyde-Activating GFA
4001	4001	1.7	Hypothetical Protein 4001
4027	fabF	1.8	3-oxoacyl-[acyl-carrier-protein] synthase 2
4030	fabD	1.9	Malonyl CoA-acyl carrier protein transacylase
4031	4031	2.3	Hypothetical
4033	rpmF	2.8	50S ribosomal protein L32
4034	yceD	2.5	Uncharacterized protein yceD
4035	yceF	1.6	Maf-like protein XOO0876
4036	4036	2.7	Hypothetical
4039	yebA	1.7	Uncharacterized protein yebA
4040	yeaY	4.9	Uncharacterized lipoprotein yeaY
4052	kdkA	1.8	3-deoxy-D-manno-octulosonic acid kinase
4054	4054	1.8	Hypothetical
4058	4058	2.4	Hypothetical
4059	gloB	2.2	Hydroxyacylglutathione hydrolase
4060	4060	3.1	Membrane-bound lytic murein transglycosylase D
4061	ppiD	2.3	Peptidyl-prolyl cis-trans isomerase D
4062	hup	3.3	DNA-binding protein HU
4064	clpX	1.6	ATP-dependent Clp protease ATP-binding subunit ClpX
4066	tig	3.0	Trigger factor
4067	clcA	2.7	H(+)/Cl(-) exchange transporter ClcA
4073	leuB	1.9	3-isopropylmalate dehydrogenase
4074	4074	1.7	Alkylhydroperoxidase Like Protein AhpD Family
4078	phoR	2.4	Phosphate regulon sensor protein phoR
4079	ppk	1.8	Polyphosphate kinase
4080	ppx	2.4	Exopolyphosphatase
4089	purF	2.1	Amidophosphoribosyltransferase

4090	cvpA	2.1	Colicin V Production Protein
4091	4091	2.6	Hypothetical
4100	4100	2.1	Hypothetical
4101	oprF	2.6	Outer membrane porin F
4115	gatA1	1.8	Glutamyl-tRNA(Gln) amidotransferase subunit A 1
4116	yjeP	3.0	MscS Mechanosensitive Ion Channel
4120	rplQ	3.1	50S ribosomal protein L17
4121	rpoA	3.6	DNA-directed RNA polymerase subunit alpha
4122	rpsD	3.9	30S ribosomal protein S4
4123	rpsK	5.6	30S ribosomal protein S11
4124	rpsM	6.9	30S ribosomal protein S13
4125	secY	2.1	Preprotein translocase subunit secY
4126	rplO	2.2	50S ribosomal protein L15
4127	rpmD	2.6	50S ribosomal protein L30
4128	rpsE	2.7	30S ribosomal protein S5
4129	rplR	2.7	50S ribosomal protein L18
4130	rplF	2.9	50S ribosomal protein L6
4131	rpsH	3.1	30S ribosomal protein S8
4132	rpsN	5.6	30S ribosomal protein S14
4133	rplE	2.5	50S ribosomal protein L5
4134	rplX	2.2	50S ribosomal protein L24
4135	rplN	2.0	50S ribosomal protein L14
4136	rpsQ	2.2	30S ribosomal protein S17
4137	rpmC	1.6	50S ribosomal protein L29
4138	rplP	1.5	50S ribosomal protein L16
4139	rpsC	1.9	30S ribosomal protein S3
4140	rplV	2.3	50S ribosomal protein L22
4141	rpsS	2.2	30S ribosomal protein S19
4142	rplB	2.4	50S ribosomal protein L2
4143	rplW	3.6	50S ribosomal protein L23
4144	rplD	3.7	50S ribosomal protein L4
4145	rplC	2.8	50S ribosomal protein L3
4146	rpsJ	2.5	30S ribosomal protein S10
4148	fusA	2.2	Elongation factor G
4149	rpsG	1.9	30S ribosomal protein S7
4150	rpsL	2.1	30S ribosomal protein S12
4151	rpoC	1.7	DNA-directed RNA polymerase subunit beta'
4153	rplL	3.0	50S ribosomal protein L7/L12

4154	rplJ	3.4	50S ribosomal protein L10
4155	rplA	3.7	50S ribosomal protein L1
4156	rplK	1.6	50S ribosomal protein L11
4157	nusG	1.8	Transcription antitermination protein nusG
4158	secE	1.7	Preprotein translocase subunit secE
4159	tuf1	5.7	Elongation factor Tu 1
4161	cphB	2.0	Cyanophycinase
4168	rplY	1.6	50S ribosomal protein L25
4169	prs	3.7	Ribose-phosphate pyrophosphokinase
4170	ispE	3.5	4-diphosphocytidyl-2-C-methyl-D-erythritol kinase
4173	hemA	1.7	Glutamyl-tRNA reductase
4177	4177	1.5	Hypothetical
4185	ilvE	2.2	Branched-chain-amino-acid aminotransferase
4186	yitW	1.9	MIP18 family protein yitW
4188	pntB	1.5	NAD(P) transhydrogenase subunit beta
4195	ydjA	2.4	Putative NAD(P)H nitroreductase ydjA
4196	polA	2.9	DNA polymerase I
4197	nudF	2.0	NUDIX Hydrolase
4198	4198	2.7	N-Formylglutamate Amidohydrolase
4202	pip	1.8	Proline iminopeptidase
4203	4203	2.0	Hypothetical
4209	tal	2.4	Transaldolase
4213	yfhC	2.2	Hypothetical
4214	rlmM	1.9	Ribosomal RNA large subunit methyltransferase M
4215	4215	2.0	Hypothetical
4216	ubiF	3.5	2-octaprenyl-3-methyl-6-methoxy-1,4-benzoquinol hydroxylase
4217	ubiH	2.5	2-octaprenyl-6-methoxyphenol hydroxylase
4218	4218	1.9	Hypothetical
4219	4219	1.8	Cob(I)yrinic acid a,c-diamide adenosyltransferase
4221	lptD	2.5	LPS-assembly protein lptD
4222	surA	3.8	Chaperone surA
4226	apaH	1.8	Bis(5'-nucleosyl)-tetraphosphatase, symmetrical
4230	thyA	1.9	Thymidylate synthase
4231	lgt	1.8	Prolipoprotein diacylglyceryl transferase
4232	4232	2.4	Hypothetical
4233	ygcG	2.6	UPF0603 protein ygcG
4234	lemA	2.7	Protein LemA
4238	qseC	2.8	Sensor protein qseC

4239	czcR	2.0	Transcriptional activator protein CzcR
4243	4243	3.6	Hypothetical
4244	4244	5.6	Hypothetical
4245	hpuB	4.0	Hemoglobin-haptoglobin utilization protein B
4246	4246	2.4	Hypothetical
4264	ahcY	2.5	Adenosylhomocysteinase
4271	yfcH	2.1	Epimerase family protein yfcH
4275	secA	3.3	Protein translocase subunit secA
4276	yebA	4.0	Uncharacterized metalloprotease yebA
4277	4277	2.1	Hypothetical
4279	ftsZ	2.0	Cell division protein ftsZ
4280	ftsA	2.3	Cell division protein ftsA
4281	ftsQ	3.4	Cell division protein ftsQ
4282	ddlB	2.9	D-alanine--D-alanine ligase B
4283	murC	3.0	UDP-N-acetylmuramate--L-alanine ligase
4284	murG	2.8	UDP-N-acetylglucosamine--N-acetylmuramyl- (pentapeptide) pyrophosphoryl-undecaprenol N-acetylglucosamine transferase
4285	ftsW	1.9	Cell division protein ftsW
4287	murF	2.6	UDP-N-acetylmuramoyl-tripeptide--D-alanyl-D- alanine ligase
4288	murE	2.6	UDP-N-acetylmuramoyl-L-alanyl-D-glutamate--2,6- diaminopimelate ligase
4295	rsmI	1.8	Ribosomal RNA small subunit methyltransferase I
4296	lpoA	1.7	Penicillin-binding protein activator LpoA
4305	thiL	1.8	Thiamine-monophosphate kinase
4306	nusB	2.2	N utilization substance protein B homolog
4307	ribH	2.6	6,7-dimethyl-8-ribityllumazine synthase
4308	ribB	3.1	3,4-dihydroxy-2-butanone 4-phosphate synthase
4309	ribE	4.1	Riboflavin synthase alpha chain
4317	glyA	2.0	Serine hydroxymethyltransferase
4324	4324	2.7	Cell Morphology Protein
4325	dltA	2.3	D-alanine--poly(phosphoribitol) ligase subunit 1
4326	4326	1.6	Hypothetical Protein 4326
4328	algJ	3.2	Alginate biosynthesis protein AlgJ
4329	4329	2.7	twin-arginine translocation pathway signal
4330	4330	4.1	Hypothetical
4331	nfrB	2.2	Bacteriophage N4 adsorption protein B
4332	4332	4.2	Hypothetical
4333	wecB	2.1	UDP-N-acetylglucosamine 2-epimerase
4350	purL	5.2	Phosphoribosylformylglycinamidine synthase
4355	lptG	3.3	Lipopolysaccharide export system permease protein lptG

4356	lptF	4.5	Lipopolysaccharide export system permease protein lptF
4357	pepA	2.3	Probable cytosol aminopeptidase
4358	holC	3.3	DNA polymerase III subunit chi
4360	valS	1.9	Valyl-tRNA synthetase
4366	proS	2.4	Prolyl-tRNA synthetase
4370	yrbE	3.3	Probable ABC transporter permease protein RT0041
4371	mkl	2.3	Probable ribonucleotide transport ATP-binding protein mkl
4373	4373	1.5	Hypothetical
4378	xanA	3.0	Phosphohexose mutases
4379	xanB	2.9	Xanthan biosynthesis protein xanB
4380	rfbD	4.2	dTDP-4-dehydrorhamnose reductase
4381	rmlC	2.3	dTDP-4-dehydrorhamnose 3,5-epimerase
4383	rfbB	1.7	dTDP-glucose 4,6-dehydratase
4384	etfB	1.8	Electron transfer flavoprotein subunit beta
4385	etfA	1.5	Electron transfer flavoprotein subunit alpha
4398	cbs	1.9	Putative cystathionine beta-synthase Rv1077
4404	4404	1.6	Fumarylacetoacetate Hydrolase
4405	rhlE	8.5	ATP-dependent RNA helicase rhlE
4429	yfbT	1.7	Phosphorylated carbohydrates phosphatase TM_1254
4431	rsmC	1.7	Ribosomal RNA small subunit methyltransferase C
4432	4432	3.9	Hypothetical
4433	4433	7.6	Hypothetical
4434	arcB	8.8	Aerobic respiration control sensor protein ArcB
4436	alr	3.2	Alanine racemase
4437	dadA	2.6	D-amino acid dehydrogenase small subunit
4438	lrp	1.7	Leucine-responsive regulatory protein
4439	4439	1.5	Hypothetical Protein 4439
4444	4444	1.6	Hypothetical
4456	4456	1.8	Hypothetical
4538	4538	14.8	Hypothetical
4550	4550	11.7	Hypothetical
4551	yiaA	1.8	Inner membrane protein yiaA
4557	gcp	1.8	Probable tRNA threonylcarbamoyladenosine biosynthesis protein Gcp
4558	rpsU	8.7	30S ribosomal protein S21
4559	yqeY	1.5	Uncharacterized protein yqeY
4560	rbn	2.2	UPF0761 membrane protein Daci_4966
4587	yebA	3.5	Uncharacterized metalloprotease HI_0409
4588	anmK	2.0	Anhydro-N-acetylmuramic acid kinase

4601	dut	1.9	Deoxyuridine 5'-triphosphate nucleotidohydrolase
4605	argS	2.3	Arginyl-tRNA synthetase
4606	4606	2.0	Sporulation Domain-Containing Protein
4611	yehS	1.8	Uncharacterized protein yehS
4615	4615	1.9	Hypothetical
4629	4629	2.3	Hypothetical
4632	4632	1.9	Hypothetical
4640	4640	2.0	Hypothetical
4644	4644	7.0	Hypothetical
4645	4645	6.3	Hypothetical
4646	4646	7.0	Hypothetical
4649	yigB	2.1	Uncharacterized protein yigB
4650	pepA	2.0	Probable cytosol aminopeptidase
4673	yjbJ	6.3	UPF0337 protein XCC3924
4674	4674	4.6	Entericidin EcnAB
4675	arcB	1.6	Arginase
4677	coaX	1.7	Type III pantothenate kinase
4678	birA	1.6	Bifunctional protein BirA
4679	4679	3.2	Hypothetical Protein 4679
4680	mtfA	1.9	Protein mtfA
4682	phoP	2.3	Virulence transcriptional regulatory protein phoP
4683	4683	1.8	Hypothetical
4690	mmsB	2.4	3-hydroxyisobutyrate dehydrogenase
4691	yngF	2.4	Putative enoyl-CoA hydratase/isomerase yngF
4692	crt	2.1	3-hydroxybutyryl-CoA dehydratase
4693	acdA	1.6	Acyl-CoA dehydrogenase
4695	4695	4.3	Uncharacterized HTH-type transcriptional regulator Rv0465c/MT0481
4713	4713	2.6	Plasmid Maintenance System Killer
4714	vapI	1.6	Virulence-associated protein I
4717	pncA	1.8	Pyrazinamidase/nicotinamidase
4728	nrdA	2.7	Ribonucleoside-diphosphate reductase subunit alpha
4736	4736	1.7	Cytochrome c-555
4737	4737	2.6	TetR Family Transcriptional Regulator
4738	bcd	5.9	Acyl-CoA dehydrogenase, short-chain specific
4739	yngE	7.3	Uncharacterized carboxylase YngE
4740	accA1	8.0	Acetyl-/propionyl-coenzyme A carboxylase alpha chain
4749	mls	1.8	Malate synthase
4760	4760	2.3	Hypothetical

4763	4763	1.9	Hypothetical
4765	4765	14.8	UPF0391 membrane protein XCC0220
4812	yadA	5.4	Adhesin yadA
4813	4813	2.6	Hypothetical
4821	hemY	2.8	HemY Domain-Containing Protein
4823	4823	2.9	Hypothetical
4825	sodC2	2.9	Superoxide dismutase [Cu-Zn] 2
4842	4842	1.8	Hypothetical
4848	4848	2.3	Uncharacterized protein Rv1276c/MT1313
4849	4849	2.2	Partition Protein
4870	yprA	2.6	Uncharacterized ATP-dependent helicase yprA
4871	yprB	2.9	Uncharacterized protein yprB
4872	4872	2.4	Lipoprotein
4873	4873	3.2	Peptidase
4874	4874	4.1	Transcriptional Regulator
4875	flp	9.6	Protein flp
4876	4876	14.0	Hypothetical Protein 4876
4877	yhxC	18.3	Uncharacterized oxidoreductase yhxC
4878	4878	3.7	Hypothetical
4879	4879	2.0	DNA poisomerase
4897	tdk	1.5	Thymidine kinase
4917	phhA	1.8	Phenylalanine-4-hydroxylase
4918	4918	2.3	Hypothetical
4932	4932	1.7	Cysteine Dioxygenase Type I
4948	4948	1.5	Hypothetical
4949	yncD	2.3	Probable tonB-dependent receptor yncD
4969	exbD2	2.1	Biopolymer transport protein exbD2
4970	exbD1	1.8	Biopolymer transport protein exbD1
4971	exbB	1.9	Biopolymer transport protein exbB
4972	tonB	3.1	Protein tonB
4973	4973	2.7	Hypothetical
4974	yfgC	2.2	TPR repeat-containing protein YPO3069/y1412/YP_2691
4976	gyrB	1.6	DNA gyrase subunit B
4981	rpmH	3.8	50S ribosomal protein L34
4982	rnpA	2.1	Ribonuclease P protein component
5007	mlaA	1.6	Probable phospholipid-binding lipoprotein mlaA
5008	5008	1.6	Hypothetical
5009	mlaC	2.7	Probable phospholipid-binding protein mlaC

5011	mlaE	2.3	Probable phospholipid ABC transporter permease protein mlaE
5012	yrbF	4.1	Uncharacterized ABC transporter ATP-binding protein HI_1087
5015	recD	1.6	Exodeoxyribonuclease V alpha chain
