# Supplementary material for: Root-microbe systems: the effect and mode of interaction of Stress Protecting Agent (SPA) Stenotrophomonas rhizophila DSM14405T
Source: Front Plant Sci. 2013 May 14;4:141. doi: 10.3389/fpls.2013.00141 (PMC3653106; doi:10.3389/fpls.2013.00141)
Supplement: Table S1A — Significantly up-regulated genes in S. rhizophila DSM14405T under salt shock. [file DataSheet1.ZIP › 51567_Berg_Table_S1B.PDF]

Supplementary Table 1b: significantly down-regulated genes in *S. rhizophila* DSM14405T under salt shock

locus tag	gene	fold change	product
1001	tcp	0.6	Methyl-accepting chemotaxis citrate transducer
1002	1002	0.6	Hydrolase
1007	tatC	0.5	Sec-independent protein translocase protein tatC
1008	1008	0.5	Hypothetical
1010	tapB	0.6	Type IV pilus assembly protein tapB
1011	glyQ	0.5	Glycyl-tRNA synthetase alpha subunit
1017	yebN	0.4	UPF0059 membrane protein Smal_3977
1018	agmR	0.6	Glycerol metabolism activator
1019	acsA	0.5	Acetyl-coenzyme A synthetase
1020	1020	0.4	Hypothetical
1021	shiA	0.4	Shikimate transporter
1023	1023	0.4	Hypothetical
1026	1026	0.5	Putative 4'-phosphopantetheinyl transferase slr0495
1031	1031	0.5	Hypothetical
1034	1034	0.6	Lipid A Biosynthesis Domain-Containing Protein
1035	1035	0.4	Hypothetical
1040	1040	0.4	Hypothetical
1042	1042	0.4	Hypothetical
1043	1043	0.5	Uncharacterized glycosyltransferase Rv1524/MT1575
1048	1048	0.5	Metallo-beta-lactamase L1
1050	1050	0.4	Hypothetical
1054	1054	0.6	Hypothetical Protein 1054
1055	nodL	0.4	Nodulation protein L
1062	1062	0.6	Hypothetical
1063	1063	0.6	Hypothetical Protein 1063
1064	1064	0.5	NUDIX Hydrolase
1065	ptpA	0.5	Prolyl tripeptidyl peptidase
1066	cydA	0.4	Cytochrome d ubiquinol oxidase subunit 1
1067	cydB	0.5	Cytochrome d ubiquinol oxidase subunit 2
1078	1078	0.5	Thioesterase Superfamily Protein
1079	1079	0.4	Hypothetical
1080	1080	0.6	Hypothetical
1081	cmdE	0.5	Tryptophan 2-halogenase
1091	1091	0.3	Hypothetical
1094	mgtE	0.5	Magnesium transporter mgtE

1099	ytbD	0.4	Uncharacterized MFS-type transporter ytbD
1101	tcp	0.5	Methyl-accepting chemotaxis citrate transducer
1104	rtcB	0.5	RNA-splicing ligase RtcB
1107	1107	0.6	Hypothetical
1111	1111	0.5	Hypothetical Protein 1111
1118	yedQ	0.5	Cellulose synthesis regulatory protein
1119	yuaR	0.5	Putative hydrolase Rv2224c/MT2282
1120	natA	0.5	ATP-binding transport protein NatA
1121	natB	0.5	Protein natB
1122	yeaM	0.5	Uncharacterized HTH-type transcriptional regulator yeaM
1123	1123	0.3	Hypothetical Protein 1123
1124	1124	0.3	Hypothetical Protein 1124
1125	1125	0.2	Hypothetical
1127	yqhA	0.4	UPF0114 protein PM1258
1128	yfcG	0.4	Probable disulfide bond reductase yfcG
1132	1132	0.5	Hypothetical
1133	1133	0.4	Hypothetical Protein 1133
1137	1137	0.6	OmpA/MotB Domain Protein
1144	1144	0.6	Hypothetical
1145	ilvD	0.5	Dihydroxy-acid dehydratase
1167	yieJ	0.6	UPF0167 protein Rv2295/MT2352
1168	comF	0.4	Competence protein F
1180	btuB	0.4	Vitamin B12 transporter BtuB
1182	1182	0.6	Hypothetical Protein 1182
1183	1183	0.4	Hypothetical Protein 1183
1184	1184	0.4	Hypothetical
1185	1185	0.4	ECF Subfamily RNA Polymerase Sigma-24 Factor
1186	1186	0.5	Anti-FecI Sigma Factor FecR
1187	1187	0.4	Hypothetical
1188	1188	0.4	Hemolysin Activation/Secretion Protein
1189	tolQ	0.4	Protein tolQ
1190	exbD1	0.3	Biopolymer transport protein exbD1
1191	1191	0.5	Hypothetical
1192	1192	0.4	Hypothetical
1196	ubiA	0.6	4-hydroxybenzoate octaprenyltransferase
1197	atoE	0.4	Short-chain fatty acids transporter
1198	1198	0.6	HsdR Family Type I Site-Specific Deoxyribonuclease
1200	1200	0.5	Hypothetical

1201	1201	0.4	Hypothetical
1202	1202	0.4	Hypothetical Protein 1202
1203	1203	0.3	Hypothetical Protein 1203
1204	1204	0.3	Hypothetical
1207	1207	0.3	Hypothetical Protein 1207
1210	pld1	0.6	Pyridoxal 4-dehydrogenase
1212	1212	0.5	XRE Family Transcriptional Regulator
1213	taqIM	0.3	Modification methylase TaqI
1214	nodV	0.3	Nodulation protein V
1215	1215	0.2	Hypothetical
1216	1216	0.3	Hypothetical Protein 1216
1217	1217	0.4	Hypothetical
1218	1218	0.5	Hypothetical Protein 1218
1219	1219	0.4	Hypothetical Protein 1219
1220	comM	0.3	Competence protein ComM
1221	1221	0.5	Hypothetical
1224	glnB	0.6	Nitrogen regulatory protein P-II 1
1226	1226	0.6	Hypothetical
1230	1230	0.3	Hypothetical
1233	1233	0.6	Conserved Hypothetical Protein
1234	1234	0.4	Hypothetical
1243	1243	0.5	Hypothetical Protein 1243
1244	sigW	0.5	RNA polymerase sigma factor sigW
1259	1259	0.4	Hypothetical
1261	1261	0.4	Hypothetical
1270	1270	0.5	Hypothetical
1274	1274	0.3	GCN5-Related N-Acetyltransferase
1275	1275	0.2	Hypothetical Protein 1275
1276	1276	0.2	Hypothetical Protein 1276
1280	1280	0.4	Hypothetical
1292	rppH	0.6	RNA pyrophosphohydrolase
1305	lplT	0.5	Lysophospholipid transporter lplT
1307	fcuA	0.4	Ferrichrome receptor fcuA
1308	1308	0.5	Multidrug resistance protein MdtC
1309	1309	0.4	Multidrug resistance protein MdtB
1310	1310	0.5	Multidrug resistance protein mdtA
1311	yhaA	0.4	Putative amidohydrolase yhaA
1317	1317	0.6	Hypothetical

1318	1318	0.5	Roadblock/LC7 Family Protein
1319	1319	0.4	Hypothetical
1320	aceK	0.5	Isocitrate dehydrogenase kinase/phosphatase
1322	yhcK	0.5	Uncharacterized protein yhcK
1323	1323	0.3	Transcription Factor Jumonji Domain-Containing Protein
1324	1324	0.3	NB-Dependent Receptor
1325	gluP	0.4	Glucose/galactose transporter
1326	cytR	0.4	HTH-type transcriptional repressor CytR
1327	bglX	0.4	Periplasmic beta-glucosidase
1336	1336	0.4	Hypothetical
1337	1337	0.4	Hypothetical
1338	1338	0.3	Hypothetical
1344	1344	0.5	Hypothetical
1349	1349	0.5	Phospholipid/Glycerol Acyltransferase
1350	ynbB	0.5	Uncharacterized protein YnbB
1357	accC	0.6	Biotin carboxylase
1361	arsB	0.3	Arsenite resistance protein ArsB
1362	ygaV	0.5	ArsR Family Transcriptional Regulator
1363	1363	0.5	Arsenical Resistance Protein ArsH
1364	arsC	0.5	Arsenate reductase
1365	1365	0.5	Hypothetical
1366	dap	0.4	D-aminopeptidase
1367	ppsA	0.4	Probable phosphoenolpyruvate synthase
1368	1368	0.3	Hypothetical
1369	1369	0.2	Hypothetical
1370	rfbD	0.3	Hypothetical
1371	1371	0.4	Hypothetical
1372	1372	0.3	Hypothetical
1373	1373	0.5	ThiJ/PfpI Domain-Containing Protein
1374	melR	0.4	Helix-Turn-Helix Domain-Containing Protein
1375	ptxR	0.5	HTH-type transcriptional regulator ptxR
1376	azoB	0.4	NAD(P)H azoreductase
1377	1377	0.5	Hypothetical Protein 1377
1382	mauR	0.6	Mau operon transcriptional activator
1383	1383	0.5	Apocarotenoid-15,15'-oxygenase
1384	1384	0.4	Hypothetical
1395	yncD	0.3	Probable tonB-dependent receptor yncD
1396	nat	0.5	Arylamine N-acetyltransferase

1397	tse	0.5	Methyl-accepting chemotaxis serine transducer
1400	dsbD	0.6	Thiol:disulfide interchange protein DsbD
1402	bsn	0.3	Extracellular ribonuclease
1403	groS	0.4	10 kDa chaperonin
1404	groL	0.4	60 kDa chaperonin
1405	gcvA	0.4	Glycine cleavage system transcriptional activator
1406	yfcG	0.4	Probable disulfide bond reductase yfcG
1412	aroG	0.6	Phospho-2-dehydro-3-deoxyheptonate aldolase, Phe-sensitive
1413	1413	0.3	Hypothetical Protein 1413
1414	mdtC	0.6	Multidrug resistance protein MdtC
1417	yicG	0.4	UPF0126 membrane protein HI_1240
1418	yhbQ	0.3	UPF0213 protein XC_1086
1419	1419	0.5	Hypothetical
1420	1420	0.4	Hypothetical
1421	1421	0.4	Hypothetical
1422	1422	0.5	Hypothetical
1423	1423	0.4	Hypothetical Protein 1423
1424	1424	0.5	Hypothetical Protein 1424
1426	1426	0.6	Hypothetical
1428	yliJ	0.5	Uncharacterized GST-like protein yliJ
1432	norM	0.4	Probable multidrug resistance protein norM
1435	fabG	0.5	3-oxoacyl-[acyl-carrier-protein] reductase FabG
1443	smf	0.5	Protein smf
1459	1459	0.4	Hypothetical Protein 1459
1461	1461	0.3	RNA Polymerase Sigma-24 Subunit ECF Subfamily
1462	1462	0.3	Hypothetical
1463	ydfG	0.3	Uncharacterized protein ydfG
1464	1464	0.3	Hypothetical Protein 1464
1465	yafC	0.4	Uncharacterized HTH-type transcriptional regulator yafC
1466	1466	0.4	Hypothetical
1467	ohrB	0.2	Organic hydroperoxide resistance protein ohrB
1468	yneE	0.3	UPF0187 protein yneE
1469	ybjT	0.4	Hypothetical
1470	1470	0.3	Beta-Lactamase Domain-Containing Protein
1471	cpo	0.3	Non-heme chloroperoxidase
1472	1472	0.2	Hypothetical
1473	yafC	0.2	Uncharacterized HTH-type transcriptional regulator yafC
1474	1474	0.4	Transcriptional Regulator

1475	1475	0.3	Hypothetical Protein 1475
1476	1476	0.3	TetR-Family Transcriptional Regulator
1477	1477	0.4	Hypothetical Protein 1477
1478	mdtN	0.3	Multidrug resistance protein mdtN
1479	1479	0.3	Hypothetical
1480	ymdC	0.4	Uncharacterized protein YmdC
1481	1481	0.3	Hypothetical Protein 1481
1482	1482	0.5	Hypothetical
1485	1485	0.3	Filamentation Induced By CAMP Protein Fic
1486	1486	0.5	Hypothetical Protein 1486
1487	1487	0.4	Hypothetical Protein 1487
1490	fyuA	0.3	Pesticin receptor
1491	yqhC	0.4	AraC Family Transcriptional Regulator
1494	ydeK	0.4	Uncharacterized transporter ydeK
1495	1495	0.5	Antibiotic Biosynthesis Monooxygenase
1496	ybaR	0.4	Putative sulfate transporter ybaR
1497	tcp	0.3	Methyl-accepting chemotaxis citrate transducer
1498	arcD	0.5	Arginine/ornithine antiporter
1500	1500	0.6	Hypothetical Protein 1500
1502	opgH	0.5	Glucans biosynthesis glucosyltransferase H
1503	estA	0.6	Carboxylesterase 1
1504	ywpD	0.6	Putative uncharacterized protein ywpD
1505	algR	0.5	Positive alginate biosynthesis regulatory protein
1508	1508	0.4	Hypothetical
1529	1529	0.5	Hypothetical Protein 1529
1532	ntrY	0.5	Nitrogen regulation protein ntrY
1533	atoC	0.5	Acetoacetate metabolism regulatory protein AtoC
1534	1534	0.4	Hypothetical
1535	macB	0.3	Macrolide export ATP-binding/permease protein MacB
1536	macB	0.3	Macrolide export ATP-binding/permease protein MacB
1537	yknY	0.4	Uncharacterized ABC transporter ATP-binding protein YknY
1538	macA	0.4	Probable macrolide-specific efflux protein macA
1540	1540	0.3	Hypothetical
1541	1541	0.3	Hypothetical Protein 1541
1542	1542	0.4	Phage SPO1 DNA Polymerase-Related Protein
1543	1543	0.4	Radical SAM Domain-Containing Protein
1544	1544	0.3	Uncharacterized protein HI_1456
1545	1545	0.4	Glyoxalase/Bleomycin Resistance Protein/Dioxygenase

1556	sam	0.6	S-adenosylmethionine uptake transporter
1563	1563	0.3	Hypothetical Protein 1563
1564	1564	0.3	Hypothetical Protein 1564
1565	yfdZ	0.5	Probable aspartate aminotransferase 2
1566	ptrB	0.4	Protease 2
1567	ybaN	0.4	Inner membrane protein ybaN
1571	xerC	0.5	Tyrosine recombinase xerC
1576	acrA	0.5	Acriflavine resistance protein A
1577	acrB	0.5	Acriflavine resistance protein B
1581	1581	0.5	Hypothetical
1582	yfhC	0.6	Cytosine Deaminase
1588	mdoB	0.4	Uncharacterized protein HI_1246
1589	adoK	0.6	Adenosine kinase
1591	mreC	0.6	Rod shape-determining protein mreC
1593	mrdA	0.6	Penicillin-binding protein 2
1594	mrdB	0.4	Rod shape-determining protein rodA
1595	mrdB	0.3	Rod shape-determining protein rodA
1617	nahA	0.4	Beta-hexosaminidase
1618	cirA	0.3	NB-Dependent Receptor
1619	glk	0.3	Glucokinase-like protein XF_1460
1620	gluP	0.3	Glucose/galactose transporter
1621	purR	0.4	HTH-type transcriptional repressor purR
1622	glmS	0.5	Glucosamine--fructose-6-phosphate aminotransferase [isomerizing]
1623	nagA	0.5	N-acetylglucosamine-6-phosphate deacetylase
1624	1624	0.4	Hypothetical
1627	cca	0.5	Multifunctional CCA protein
1628	1628	0.5	Hypothetical Protein 1628
1630	exbD2	0.3	Biopolymer transport protein exbD2
1631	btuB	0.3	Vitamin B12 transporter BtuB
1632	rhtB	0.6	Homoserine/homoserine lactone efflux protein
1642	dsbA	0.5	Thiol:disulfide interchange protein DsbA
1646	1646	0.4	Beta-Lactamase Domain-Containing Protein
1647	ybcM	0.5	Uncharacterized HTH-type transcriptional regulator ybcM
1648	1648	0.5	Hypothetical
1651	ycaN	0.6	Uncharacterized HTH-type transcriptional regulator ycaN
1656	nemA	0.5	N-ethylmaleimide reductase
1668	cph2	0.4	Phytochrome-like protein cph2
1669	1669	0.5	Hypothetical

1670	1670	0.3	UspA Domain-Containing Protein
1671	nahR	0.5	HTH-type transcriptional activator nahR
1672	yhcA	0.4	Uncharacterized MFS-type transporter yhcA
1676	1676	0.6	DegV domain-containing protein XCC3382
1677	nahD	0.5	2-hydroxychromene-2-carboxylate isomerase
1678	1678	0.5	Amidohydrolase
1679	1679	0.4	Amidohydrolase
1680	1680	0.3	Extracellular serine protease
1682	1682	0.5	Hypothetical
1684	nodB	0.5	Chitooligosaccharide deacetylase
1686	1686	0.6	Hypothetical
1692	1692	0.3	NB-Dependent Receptor
1696	tctD	0.5	Transcriptional regulatory protein tctD
1697	qseC	0.5	Sensor protein qseC
1698	1698	0.4	Hypothetical
1702	dctA	0.3	C4-dicarboxylate transport protein
1704	1704	0.4	Secretory Protein
1705	1705	0.5	Alpha-1 2-Mannosidase
1709	yafM	0.3	Hypothetical
1710	htrB	0.4	Lipid A biosynthesis lauroyl acyltransferase
1712	1712	0.4	Magnesium transporter mgtE
1713	1713	0.4	Conserved Hypothetical Protein
1719	1719	0.4	Hypothetical Protein 1719
1722	leuD	0.5	3-isopropylmalate dehydratase small subunit
1723	leuC	0.6	3-isopropylmalate dehydratase large subunit
1724	tehB	0.6	Methyltransferase Type
1725	leuA	0.5	2-isopropylmalate synthase
1726	tdcB	0.5	Threonine dehydratase catabolic
1727	1727	0.5	Acetolactate Synthase Isozyme II Small Subunit
1728	ilvG	0.5	Acetolactate synthase isozyme 2 large subunit
1729	ilvC	0.3	Ketol-acid reductoisomerase
1733	1733	0.4	Ion Transport 2 Domain-Containing Protein
1735	1735	0.5	Transcriptional Regulator AraC Family
1736	btuB	0.3	Vitamin B12 transporter BtuB
1737	1737	0.4	Uncharacterized protein TP_0073
1739	1739	0.3	Hypothetical
1742	fecI	0.4	ECF Subfamily RNA Polymerase Sigma Factor
1743	fecR	0.5	Anti-FecI Sigma Factor FecR

1744	hemR	0.4	Hemin receptor
1746	1746	0.4	Heme Oxygenase
1747	ybaN	0.4	Inner membrane protein ybaN
1748	exbB	0.3	Biopolymer transport protein exbB
1749	exbD	0.4	Biopolymer transport protein exbD
1750	tonB	0.5	Protein tonB
1751	hppA	0.5	K(+)-insensitive pyrophosphate-energized proton pump
1760	1760	0.4	Hypothetical
1761	1761	0.4	Hypothetical Protein 1761
1770	1770	0.5	Azurin
1773	rubA2	0.6	Rubredoxin-2
1774	ychA	0.5	UPF0162 protein XF_1494
1783	pepQ	0.6	Xaa-Pro dipeptidase
1784	1784	0.5	Hypothetical
1796	1796	0.4	Hypothetical Protein 1796
1800	ygiW	0.4	Hypothetical
1801	1801	0.3	Hypothetical Protein 1801
1802	cfaB	0.3	CFA/I fimbrial subunit B
1803	cfaC	0.4	CFA/I fimbrial subunit C
1804	cfaE	0.2	CFA/I fimbrial subunit E
1805	csoB	0.2	CS1 fimbrial subunit B
1806	1806	0.4	Cell Surface Protein
1807	1807	0.3	Hypothetical Protein 1807
1808	1808	0.2	Hypothetical
1809	1809	0.3	Hypothetical
1815	1815	0.6	Pilus Assembly Protein PilP
1816	pilQ	0.5	Fimbrial assembly protein pilQ
1825	ybiA	0.4	Hypothetical
1835	ompW	0.1	Outer membrane protein W
1843	pepN	0.4	Aminopeptidase N
1844	1844	0.4	Hypothetical
1849	1849	0.4	Hypothetical Protein 1849
1850	1850	0.4	NB-Dependent Receptor
1852	acrD	0.5	Probable aminoglycoside efflux pump
1855	1855	0.3	Hypothetical
1856	yrbD	0.4	Putative sodium/proton-dependent alanine carrier protein yrbD
1865	thiS	0.4	Sulfur Carrier Protein ThiS
1867	1867	0.6	Hypothetical Protein 1867

1868	1868	0.6	Hypothetical Protein 1868
1876	tapC	0.2	Type IV pilus assembly protein tapC
1877	fimA	0.3	Fimbrial protein
1878	speE	0.2	Probable spermidine synthase
1879	pilB	0.6	Type 4 fimbrial assembly protein pilB
1895	btuB	0.6	Vitamin B12 transporter BtuB
1896	1896	0.5	Hypothetical
1897	1897	0.5	Hypothetical
1899	1899	0.6	Hypothetical Protein 1899
1903	1903	0.5	Hypothetical
1905	yddB	0.4	NB Dependent Receptor
1906	yddB	0.4	Uncharacterized protein yddB
1910	mdeA	0.3	Methionine gamma-lyase
1911	ygiV	0.5	AraC Family Transcriptional Regulator
1930	ybgC	0.6	Acyl-CoA thioester hydrolase YbgC
1934	1934	0.6	LA Protein
1948	1948	0.4	Hypothetical
1949	1949	0.4	Hypothetical Protein 1949
1956	copB	0.4	Copper resistance protein B
1957	copA	0.4	Copper resistance protein A
1958	1958	0.6	Hypothetical Protein 1958
1960	1960	0.4	Hypothetical Protein 1960
1963	1963	0.5	Hypothetical
1973	pilG	0.4	Protein pilG
1974	pilH	0.3	Protein pilH
1975	pilI	0.4	Protein PilI
1976	pilJ	0.4	Protein pilJ
1977	cheA	0.6	Chemotaxis protein CheA
1982	nudE	0.5	ADP compounds hydrolase nudE
1983	cysQ	0.6	3'(2'),5'-bisphosphate nucleotidase CysQ
1985	ynfA	0.5	UPF0060 membrane protein Mfla_0485
1986	1986	0.4	Hypothetical
1987	qmcA	0.3	Protein QmcA
1988	ybbJ	0.3	Inner membrane protein ybbJ
1993	alkJ	0.5	Alcohol dehydrogenase [acceptor]
1994	1994	0.4	Hypothetical
1995	1995	0.3	Hypothetical
1996	1996	0.4	Hypothetical

2003	mtgA	0.6	Monofunctional biosynthetic peptidoglycan transglycosylase
2007	yqfA	0.6	Hemolysin-3
2008	prfC	0.6	Peptide chain release factor 3
2009	yebA	0.5	Uncharacterized metalloprotease bbp_296
2010	ydeU	0.4	Uncharacterized protein YdeU
2011	yhaH	0.2	Inner membrane protein yhaH
2012	insF1	0.2	Transposase insF for insertion sequence IS3A
2013	2013	0.2	Hypothetical Protein 2013
2014	2014	0.3	Hypothetical Protein 2014
2015	2015	0.5	Conserved Hypothetical Protein
2016	2016	0.2	Hypothetical Protein 2016
2017	2017	0.4	Hypothetical Protein 2017
2018	2018	0.4	Hypothetical Protein 2018
2019	2019	0.4	Hypothetical Protein 2019
2020	2020	0.3	Hypothetical Protein 2020
2021	2021	0.4	Hypothetical
2026	dhmA	0.4	Haloalkane dehalogenase
2028	2028	0.4	Hypothetical
2030	yceJ	0.4	Cytochrome b561 homolog 2
2031	yceI	0.4	UPF0312 protein PSPPH_0448
2033	luxQ	0.5	Autoinducer 2 sensor kinase/phosphatase luxQ
2034	rpfC	0.5	Sensory/regulatory protein RpfC
2058	pilH	0.6	Protein pilH
2062	2062	0.4	Hypothetical
2064	2064	0.5	Hypothetical
2065	2065	0.4	Hypothetical
2066	tse	0.4	Methyl-accepting chemotaxis serine transducer
2067	2067	0.5	Hypothetical
2070	rhaS	0.4	HTH-type transcriptional activator rhaS
2073	bcr	0.3	Bicyclomycin resistance protein
2074	viuB	0.3	Vibriobactin utilization protein viuB
2076	2076	0.4	Peptidyl-Dipeptidase A
2079	arnT1	0.6	Undecaprenyl phosphate-alpha-4-amino-4-deoxy-L-arabinose arabinosyl transferase 1
2080	mprA	0.6	Response regulator mprA
2081	qseC	0.4	Sensor protein qseC
2083	2083	0.3	Hypothetical
2084	ybdD	0.6	Uncharacterized protein ybdD
2085	yjiY	0.5	Inner membrane protein YjiY

2087	2087	0.5	Hypothetical
2088	aqpZ	0.2	Aquaporin Z
2102	2102	0.5	Hypothetical
2103	2103	0.5	Adenylate Cyclase
2104	2104	0.4	DSBA Oxidoreductase
2109	kmo	0.4	Kynurenine 3-monooxygenase
2113	lig	0.5	Probable DNA ligase
2114	lig	0.5	Probable DNA ligase
2122	ymdC	0.5	Uncharacterized protein YmdC
2123	rsuA	0.5	Ribosomal small subunit pseudouridine synthase A
2124	ygjP	0.6	Uncharacterized protein ygjP
2127	2127	0.4	Hypothetical
2135	yegD	0.4	Uncharacterized chaperone protein yegD
2142	mprF	0.6	Phosphatidylglycerol lysyltransferase
2147	2147	0.3	Hypothetical
2148	ywkD	0.5	Uncharacterized protein ywkD
2149	pbpE	0.5	Penicillin-binding protein 4*
2150	2150	0.5	Hypothetical
2151	ypdP	0.4	Uncharacterized protein ypdP
2152	2152	0.5	Hypothetical
2153	2153	0.6	Hypothetical
2155	2155	0.6	Hypothetical
2158	ybbN	0.5	Uncharacterized protein ybbN
2159	2159	0.6	Hypothetical
2166	btuB	0.3	Vitamin B12 transporter BtuB
2167	tonB	0.2	Protein tonB
2168	bp26	0.5	26 kDa periplasmic immunogenic protein
2178	tilS	0.5	tRNA(Ile)-lysidine synthase
2179	2179	0.3	Conserved Hypothetical Protein
2180	phoA	0.5	Alkaline phosphatase
2181	dctA2	0.4	C4-dicarboxylate transport protein 2
2182	2182	0.4	Hypothetical
2183	2183	0.5	Hypothetical
2184	2184	0.4	Hypothetical
2185	2185	0.4	Peptidase
2186	2186	0.4	Hypothetical
2187	2187	0.3	Hypothetical
2188	zraR	0.4	Transcriptional regulatory protein zraR

2190	ydhO	0.6	Uncharacterized protein ydhO
2192	yxaH	0.6	Uncharacterized protein yxaH
2194	ycbK	0.6	Uncharacterized transporter ycbK
2195	ybaZ	0.5	Methylated-DNA-(Protein)-Cysteine S-Methyltransferase
2197	2197	0.4	Saccharopine Dehydrogenase
2199	pepO	0.4	Neutral endopeptidase
2200	p49	0.5	Protein p49
2201	2201	0.5	Hypothetical
2202	yuxL	0.3	Uncharacterized peptidase yuxL
2203	cirA	0.3	Colicin I receptor
2211	2211	0.5	Hypothetical
2212	greB	0.4	Transcription elongation factor greB
2213	yjgR	0.5	Uncharacterized protein yjgR
2216	psd	0.5	Phosphatidylserine decarboxylase proenzyme
2217	scoP	0.4	Putative copper-binding protein
2264	2264	0.5	Hypothetical
2265	chiA1	0.5	Chitinase A1
2268	2268	0.4	Beta lactamase
2285	2285	0.5	Hypothetical Protein 2285
2286	putR	0.5	Proline dehydrogenase transcriptional activator
2288	panB	0.6	3-methyl-2-oxobutanoate hydroxymethyltransferase
2289	2289	0.5	Hypothetical Protein 2289
2290	2290	0.4	Hypothetical Protein 2290
2291	yafP	0.5	Acetyltransferase
2293	2293	0.5	Conserved Hypothetical Protein
2294	2294	0.4	Hypothetical Protein 2294
2295	2295	0.4	Hypothetical Protein 2295
2296	yiaT	0.3	Putative outer membrane protein yiaT
2307	mmgC	0.4	Acyl-CoA dehydrogenase
2308	2308	0.6	Hypothetical
2309	2309	0.3	Hypothetical Protein 2309
2310	fecA	0.2	NB-Dependent Receptor
2311	fecI	0.3	Probable RNA polymerase sigma factor fecI
2312	fecR	0.4	Anti-FecI Sigma Factor FecR
2313	2313	0.5	Amidohydrolase
2315	blc	0.4	Outer membrane lipoprotein blc
2316	2316	0.3	Hypothetical Protein 2316
2317	yjiJ	0.3	Uncharacterized protein yjiJ

2319	2319	0.6	Hypothetical
2321	yncB	0.6	Putative NADP-dependent oxidoreductase yncB
2324	2324	0.3	Hypothetical Protein 2324
2325	mcpA	0.5	Chemoreceptor mcpA
2326	cirA	0.3	NB-Dependent Receptor
2327	2327	0.5	Hypothetical Protein 2327
2328	2328	0.5	Hypothetical
2329	ampH	0.5	D-alanyl-D-alanine carboxypeptidase
2341	2341	0.5	Uncharacterized protein in phbA 5'region
2342	2342	0.3	Hypothetical Protein 2342
2343	phbB	0.6	Acetoacetyl-CoA reductase
2346	2346	0.5	Hypothetical Protein 2346
2349	2349	0.5	Alkylphosphonate ABC Transporter
2350	yciR	0.6	Uncharacterized signaling protein CC_0091
2374	2374	0.5	Hypothetical
2375	pgaC	0.4	Poly-beta-1,6-N-acetyl-D-glucosamine synthase
2376	pgaB	0.4	Poly-beta-1,6-N-acetyl-D-glucosamine N-deacetylase
2377	pgaA	0.6	Poly-beta-1,6-N-acetyl-D-glucosamine export protein
2378	yycB	0.5	Uncharacterized transporter YycB
2380	cydB	0.5	Cytochrome d ubiquinol oxidase subunit 2
2381	cydA	0.3	Cytochrome d ubiquinol oxidase subunit 1
2382	aarD	0.5	Transport ATP-binding protein AarD
2383	cydC	0.5	ATP-binding/permease protein CydC
2384	2384	0.2	Hypothetical Protein 2384
2385	2385	0.2	Hypothetical Protein 2385
2386	2386	0.2	Hypothetical
2387	2387	0.3	Acetyltransferase
2388	2388	0.3	Hypothetical Protein 2388
2389	2389	0.4	Hypothetical Protein 2389
2390	2390	0.5	Hypothetical
2394	dsbE	0.6	Thiol:disulfide interchange protein DsbE
2395	ccmF	0.5	Cytochrome c-type biogenesis protein CcmF
2399	ccmB	0.4	Heme exporter protein B
2400	ccmA	0.5	Cytochrome c biogenesis ATP-binding export protein CcmA
2401	ybdL	0.5	Aminotransferase YbdL
2402	yafV	0.5	UPF0012 hydrolase yafV
2403	2403	0.6	Hypothetical
2417	2417	0.4	Hypothetical

2420	zupT	0.5	Zinc transporter ZupT
2425	insJ	0.6	Transposase
2437	2437	0.5	Hypothetical Protein 2437
2438	2438	0.6	Hypothetical Protein 2438
2440	btuE	0.6	Putative glutathione peroxidase
2444	ydfG	0.5	Uncharacterized protein ydfG
2445	fecI	0.6	RNA Polymerase Sigma-24 Subunit ECF Subfamily
2452	2452	0.5	Hypothetical
2459	ycfJ	0.5	Uncharacterized protein ycfJ
2461	pdhB	0.5	Pyruvate dehydrogenase E1 component subunit beta
2465	2465	0.4	Hypothetical Protein 2465
2466	2466	0.6	Hypothetical
2481	2481	0.5	Hypothetical Protein 2481
2487	2487	0.5	Amidohydrolase
2488	hcaT	0.3	Probable 3-phenylpropionic acid transporter
2493	2493	0.5	Hypothetical
2498	ppnK	0.5	Probable inorganic polyphosphate/ATP-NAD kinase
2527	sldA	0.5	Glycerol dehydrogenase large subunit
2529	2529	0.6	Hypothetical
2530	yafM	0.3	Hypothetical
2531	2531	0.4	Hypothetical Protein 2531
2533	2533	0.5	Hypothetical
2534	2534	0.5	Hypothetical Protein 2534
2535	2535	0.5	Hypothetical
2537	ylbK	0.3	Uncharacterized NTE family protein ylbK
2538	yhcK	0.3	Uncharacterized protein yhcK
2540	2540	0.6	Hypothetical Protein 2540
2541	yfhB	0.5	Uncharacterized protein yfhB
2542	2542	0.6	Lipolytic Protein G-D-S-L Family
2543	2543	0.3	Hypothetical
2549	2549	0.5	Restriction Endonuclease
2550	2550	0.2	Conserved Hypothetical Protein
2560	2560	0.4	Hypothetical
2568	2568	0.3	SH3 Domain Protein
2572	2572	0.4	Hypothetical
2573	2573	0.3	Hypothetical
2574	2574	0.5	Virulence Regulator
2575	alkR	0.3	HTH-type transcriptional regulator AlkR

2576	nepI	0.3	Purine ribonucleoside efflux pump nepI
2577	yehU	0.2	Signal Transduction Histidine Kinase LytS
2578	lytT	0.2	Sensory transduction protein lytT
2579	ycjY	0.2	Acyl Co A Thioester Hydrolase
2580	thiD	0.2	Hydroxymethylpyrimidine/phosphomethylpyrimidine kinase
2581	2581	0.2	Abortive Infection Protein
2582	sigK	0.2	RNA polymerase sigma factor sigK
2583	chrR	0.2	Transcriptional activator ChrR
2584	tcp	0.3	Methyl-accepting chemotaxis citrate transducer
2585	yddU	0.3	Uncharacterized protein y4IL
2586	2586	0.5	Hypothetical Protein 2586
2594	qor	0.4	Zinc-type alcohol dehydrogenase-like protein SAV2186
2595	sdh	0.5	Serine 3-dehydrogenase
2596	nemA	0.5	N-ethylmaleimide reductase
2598	2598	0.6	TetR Family Transcriptional Regulator
2599	yegM	0.4	RND Family Efflux Transporter MFP Subunit
2600	2600	0.3	Probable aminoglycoside efflux pump
2601	fabF	0.4	3-oxoacyl-[acyl-carrier-protein] synthase 2
2602	2602	0.4	Hypothetical Protein 2602
2605	2605	0.4	Hypothetical
2606	2606	0.4	Hypothetical Protein 2606
2608	ygfF	0.5	Uncharacterized oxidoreductase MexAM1_META1p0182
2610	2610	0.5	Saccharopine Dehydrogenase
2611	2611	0.4	Hypothetical
2612	alsR	0.6	HTH-type transcriptional regulator AlsR
2613	ytbD	0.3	Uncharacterized MFS-type transporter ytbD
2615	2615	0.4	Hypothetical Protein 2615
2616	arsB	0.4	Arsenical pump membrane protein
2617	2617	0.3	Uncharacterized MFS-type transporter Rv2456c/MT2531
2618	2618	0.3	Hypothetical
2619	eptA	0.3	Phosphoethanolamine transferase eptA
2620	dgkA	0.2	Diacylglycerol kinase
2621	tcrY	0.3	Probable sensor histidine kinase TcrY
2622	qseB	0.4	Transcriptional regulatory protein qseB
2623	2623	0.2	Hypothetical Protein 2623
2624	2624	0.4	Hypothetical
2625	yncD	0.4	NB-Dependent Receptor
2627	yjbJ	0.4	UPF0337 protein XCC0070

2630	2630	0.6	Glyoxalase/Bleomycin Resistance Protein/Dioxygenase
2631	rsbRA	0.4	RsbT co-antagonist protein rsbRA
2632	rsbS	0.5	RsbT antagonist protein rsbS
2633	rsbT	0.5	Serine/threonine-protein kinase rsbT
2634	2634	0.5	Stage II Sporulation Protein E
2635	gacS	0.5	Sensor protein gacS
2648	2648	0.4	Hypothetical
2649	2649	0.5	Hypothetical
2654	nemA	0.6	N-ethylmaleimide reductase
2656	ydiA	0.4	Putative phosphotransferase Smal_2421
2668	yegE	0.5	Probable diguanylate cyclase YegE
2673	2673	0.6	GCN5-Related N-Acetyltransferase
2676	rhaR	0.4	AraC Family Transcriptional Regulator
2684	2684	0.6	Hypothetical Protein 2684
2688	ypdA	0.5	Inner membrane protein ypdA
2689	2689	0.3	Hypothetical Protein 2689
2690	2690	0.3	Hypothetical Protein 2690
2691	gstB	0.4	Glutathione S-transferase GST-6.0
2695	yjgH	0.6	RutC family protein yjgH
2701	2701	0.3	Hypothetical Protein 2701
2702	gcvA	0.4	Glycine cleavage system transcriptional activator
2703	2703	0.6	Hypothetical Protein 2703
2704	2704	0.6	Hypothetical
2709	dnaE2	0.6	Error-prone DNA polymerase
2712	2712	0.3	Hypothetical
2714	ygaY	0.4	Uncharacterized transporter ygaY
2718	emrB	0.4	Multidrug resistance protein B homolog
2719	2719	0.5	Hypothetical
2720	2720	0.4	Hypothetical Protein 2720
2721	cocE	0.4	Cocaine esterase
2722	2722	0.5	Hypothetical
2738	2738	0.3	Hypothetical Protein 2738
2739	2739	0.3	Conserved Hypothetical Protein
2740	2740	0.3	Hypothetical
2759	2759	0.5	Hypothetical
2760	2760	0.3	Hypothetical
2763	2763	0.3	Hypothetical
2764	2764	0.2	Hypothetical Protein 2764

2765	2765	0.3	Sulfatase Modifying Factor 1 -Like Protein
2766	2766	0.3	Hypothetical
2767	2767	0.4	Hypothetical Protein 2767
2768	2768	0.3	Hypothetical Protein 2768
2769	2769	0.3	Hypothetical
2770	2770	0.3	Hypothetical Protein 2770
2771	2771	0.4	Hypothetical Protein 2771
2772	2772	0.3	Hypothetical Protein 2772
2784	2784	0.6	Hypothetical Protein 2784
2796	2796	0.6	Hypothetical
2798	irgA	0.4	Iron-regulated outer membrane virulence protein
2799	rhtC	0.3	Threonine efflux protein
2800	araC	0.4	AraC Family Transcriptional Regulator
2801	2801	0.5	GCN5-Related N-Acetyltransferase
2802	2802	0.5	Hypothetical Protein 2802
2803	nylB'	0.4	6-aminohexanoate-dimer hydrolase
2805	2805	0.6	Hypothetical
2818	yadA	0.6	Adhesin yadA
2819	ompA	0.5	Outer membrane protein A
2820	2820	0.1	Uncharacterized protein sll1483
2821	2821	0.3	OsmC Family Protein
2822	2822	0.4	Hypothetical
2823	2823	0.4	6-hydroxy-D-nicotine oxidase
2824	2824	0.5	Hypothetical
2825	actP	0.6	Copper-transporting P-type ATPase
2827	2827	0.6	Heavy Metal Transport/Detoxification Protein
2834	2834	0.4	Acetamidase/Formamidase
2835	2835	0.4	Hypothetical Protein 2835
2836	2836	0.3	NAD-Dependent Epimerase/Dehydratase
2837	ytfH	0.4	Uncharacterized HTH-type transcriptional regulator ytfH
2838	2838	0.4	Hypothetical
2839	ydcR	0.5	Uncharacterized HTH-type transcriptional regulator RHOS4_30730
2840	cyoA	0.3	Ubiquinol oxidase subunit 2
2841	cyoB	0.3	Ubiquinol oxidase subunit 1
2842	cyoC	0.4	Cytochrome o ubiquinol oxidase subunit 3
2843	cyoD	0.5	Cytochrome o ubiquinol oxidase protein CyoD
2845	2845	0.4	SCO2-like protein RBE_0699
2846	stp	0.5	Multidrug resistance protein stp

2851	ygaY	0.4	Uncharacterized transporter ygaY
2852	yxjL	0.4	Uncharacterized transcriptional regulatory protein yxjL
2853	ydfH	0.6	Sensor histidine kinase ydfH
2854	2854	0.4	Hypothetical Protein 2854
2855	rhaR	0.4	AraC Family Transcriptional Regulator
2856	ycaC	0.3	Uncharacterized protein ycaC
2857	2857	0.3	DoxD-Like Family Membrane Protein
2858	nfdA	0.3	N-substituted formamide deformylase
2859	2859	0.4	Hypothetical
2860	2860	0.5	Hypothetical
2863	cpo	0.3	Non-heme chloroperoxidase
2876	glpR	0.5	Glycerol-3-phosphate regulon repressor
2882	nrdB	0.6	Ribonucleoside-diphosphate reductase subunit beta
2884	yflS	0.6	Putative malate transporter yflS
2893	2893	0.6	Disulphide-Isomerase
2896	yhiD	0.4	Uncharacterized protein slr0014
2897	mgtA	0.4	Magnesium-transporting ATPase, P-type 1
2898	2898	0.4	Hypothetical
2899	2899	0.6	Hypothetical
2907	2907	0.4	Hypothetical
2908	mepA	0.3	Tyrosinase
2909	2909	0.4	Hypothetical
2910	ydjG	0.5	Uncharacterized protein ydjG
2912	2912	0.4	Hypothetical
2913	yuaQ	0.4	Uncharacterized protein YuaQ
2914	2914	0.3	Hypothetical Protein 2914
2915	2915	0.4	Hypothetical Protein 2915
2916	2916	0.3	Hypothetical Protein 2916
2917	2917	0.4	Hypothetical Protein 2917
2918	2918	0.3	Hypothetical Protein 2918
2921	2921	0.5	Hypothetical
2923	2923	0.5	Hypothetical
2924	2924	0.4	Hypothetical Protein 2924
2925	2925	0.3	Hypothetical Protein 2925
2926	2926	0.3	Hypothetical Protein 2926
2929	2929	0.4	Hypothetical
2930	ycjZ	0.3	Uncharacterized HTH-type transcriptional regulator ycjZ
2931	2931	0.5	Hypothetical

2932	fabG	0.4	3-oxoacyl-[acyl-carrier-protein] reductase FabG
2933	ndvB	0.6	Protein ndvB
2934	2934	0.3	Hypothetical Protein 2934
2935	2935	0.4	Hypothetical
2936	2936	0.6	Hypothetical
2937	2937	0.3	UPF0173 metal-dependent hydrolase Rxyl_1261
2938	2938	0.3	Hypothetical Protein 2938
2939	gcvA	0.6	Glycine cleavage system transcriptional activator
2940	yihR	0.5	Uncharacterized protein yihR
2941	xsa	0.3	Xylosidase/arabinosidase
2942	cbg-1	0.5	Beta-glucosidase
2943	xylB	0.4	Xylosidase/arabinosidase
2944	2944	0.5	Sialate O-Acetylesterase
2945	aguA	0.5	Alpha-glucuronidase
2947	bglX	0.6	Periplasmic beta-glucosidase
2948	gtr	0.5	Glucose transport protein
2949	xylA1	0.4	Xylose isomerase 1
2950	xylB	0.4	Xylulose kinase
2951	2951	0.5	Uncharacterized protein y4xG
2952	2952	0.4	Transcription Factor Jumonji Domain-Containing Protein
2953	2953	0.3	SapC Family Protein
2954	2954	0.3	NB-Dependent Receptor
2959	phaZ1	0.4	Poly(3-hydroxyalkanoate) depolymerase C
2960	yxjC	0.4	Uncharacterized transporter YxjC
2961	fyuA	0.3	Pesticin receptor
2962	ybfF	0.6	Alpha/Beta Hydrolase Fold Protein
2963	maoC	0.4	Probable enoyl-CoA hydratase 1
2964	torS	0.6	Sensor protein torS
2966	2966	0.4	D-(-)-3-hydroxybutyrate oligomer hydrolase
2967	alkK	0.4	Medium-chain-fatty-acid--CoA ligase
2979	yuaQ	0.6	Uncharacterized protein YuaQ
2981	2981	0.3	Hypothetical
2982	aer	0.4	Aerotaxis receptor
2986	2986	0.6	Acetyltransferase
2987	fusA	0.5	Elongation factor G
2988	2988	0.4	Hypothetical
2997	ycaK	0.6	Uncharacterized NAD(P)H oxidoreductase HI_1544
2998	yhaI	0.4	Inner membrane protein yhaI

3000	gstA	0.4	Protein gstA
3001	3001	0.6	Helix-Turn-Helix Type
3002	lrhA	0.5	Probable HTH-type transcriptional regulator lrhA
3003	rcsB	0.4	Capsular synthesis regulator component B
3009	3009	0.4	Hypothetical
3012	3012	0.5	Hypothetical
3013	3013	0.4	Hypothetical
3014	aat	0.5	Leucyl/phenylalanyl-tRNA--protein transferase
3016	3016	0.6	Hypothetical
3025	3025	0.5	Hypothetical Protein 3025
3026	ycaQ	0.4	Uncharacterized protein ycaQ
3027	3027	0.4	Hypothetical Protein 3027
3028	3028	0.3	Hypothetical Protein 3028
3029	yciR	0.5	Uncharacterized signaling protein PA1727
3035	3035	0.5	Flagellar Basal Body P-Ring Biosynthesis Protein FlgA
3037	3037	0.3	Hypothetical Protein 3037
3038	flgB	0.3	Flagellar basal-body rod protein flgB
3039	flgC	0.3	Flagellar basal-body rod protein flgC
3040	flgD	0.3	Basal-body rod modification protein flgD
3041	flgE	0.3	Flagellar hook protein flgE
3042	flgF	0.3	Flagellar basal-body rod protein flgF
3043	flgG	0.3	Flagellar basal-body rod protein flgG
3044	flgH	0.4	Flagellar L-ring protein
3045	flgI	0.4	Flagellar P-ring protein
3046	flgJ	0.6	Peptidoglycan hydrolase flgJ
3047	flgK	0.4	Flagellar hook-associated protein 1
3048	flgL	0.5	Flagellar hook-associated protein 3
3049	3049	0.5	Hypothetical Protein 3049
3062	3062	0.2	Hypothetical
3063	fliE	0.3	Flagellar hook-basal body complex protein FliE
3064	fliF	0.3	Flagellar M-ring protein
3065	fliG	0.4	Flagellar motor switch protein FliG
3066	3066	0.4	Flagellar Assembly Protein FliH
3067	fliI	0.4	Flagellum-specific ATP synthase
3069	fliK	0.4	Flagellar hook-length control protein
3070	fliL	0.4	Flagellar Basal Body-Associated Protein FliL
3071	fliM	0.3	Flagellar motor switch protein FliM
3072	fliN	0.3	Flagellar motor switch protein FliN

3073	fliO	0.3	Flagellar protein fliO
3074	fliP	0.3	Flagellar biosynthetic protein fliP
3075	fliQ	0.4	Export
3076	fliR	0.3	Flagellar biosynthetic protein fliR
3082	flhB	0.3	Flagellar biosynthetic protein flhB
3083	flhA	0.3	Flagellar biosynthesis protein flhA
3084	flhF	0.4	Flagellar biosynthesis protein flhF
3085	minD	0.4	Uncharacterized ATP-binding protein MJ0547
3086	fliA	0.4	RNA polymerase sigma factor for flagellar operon
3087	cheY	0.4	Chemotaxis protein CheY
3088	cheZ	0.4	Protein phosphatase CheZ
3089	cheA	0.4	Chemotaxis protein CheA
3090	motA	0.4	Motility protein A
3091	motB	0.5	Motility protein B
3092	soj	0.5	Sporulation initiation inhibitor protein soj
3093	cheW	0.4	Chemotaxis Signal Transduction Protein
3095	cheY	0.6	Chemotaxis protein CheY
3097	tse	0.4	Methyl-accepting chemotaxis serine transducer
3098	ycgR	0.3	Flagellar brake protein YcgR
3099	cheW	0.4	Chemotaxis protein CheW
3111	3111	0.2	Hypothetical
3112	rpfF	0.2	Fatty acid oxidation complex subunit alpha
3119	yfaP	0.6	Uncharacterized protein yfaP
3120	ypfG	0.4	Hypothetical
3121	ypfG	0.3	Uncharacterized protein ypfG
3122	3122	0.5	Hypothetical Protein 3122
3137	ykoW	0.6	Signaling protein ykoW
3142	3142	0.4	Hypothetical
3143	acrB	0.3	Acriflavine resistance protein B
3144	mexA	0.3	Multidrug resistance protein mexA
3147	3147	0.6	Hypothetical
3151	3151	0.4	NUDIX Hydrolase
3153	yhdG	0.4	Uncharacterized amino acid permease YhdG
3157	3157	0.4	Hypothetical
3158	csxA	0.4	Exo-beta-D-glucosaminidase
3159	yihS	0.4	Uncharacterized sugar isomerase yihS
3160	scrK	0.6	Fructokinase
3161	gluP	0.6	Glucose/galactose transporter

3164	cirA	0.4	NB-Dependent Receptor
3185	ytfF	0.4	Inner membrane protein ytfF
3191	3191	0.5	Hypothetical
3202	3202	0.3	Hypothetical Protein 3202
3203	3203	0.4	Hypothetical
3205	3205	0.5	Uncharacterized 22.5 kDa protein in cps region
3210	yjbH	0.4	Uncharacterized lipoprotein yjbH
3211	kdsD	0.2	Arabinose 5-phosphate isomerase KdsD
3212	kdsC	0.2	3-deoxy-D-manno-octulosonate 8-phosphate phosphatase KdsC
3213	3213	0.2	Hypothetical Protein 3213
3214	3214	0.1	Hypothetical Protein 3214
3215	3215	0.1	Capsule Polysaccharide Biosynthesis Protein
3216	3216	0.2	Hypothetical
3217	rfbB	0.2	Putative UDP-glucose 4-epimerase
3218	wzc	0.2	Tyrosine-protein kinase wzc
3219	wzb	0.3	Low molecular weight protein-tyrosine-phosphatase wzb
3229	ypjA	0.3	Ig Family Protein
3230	3230	0.3	Tail Collar Domain-Containing Protein
3231	3231	0.4	Tail Collar Domain-Containing Protein
3232	3232	0.5	Tail Collar Domain-Containing Protein
3233	3233	0.5	GCN5-Like N-Acetyltransferase
3234	3234	0.4	Hypothetical
3235	neo	0.5	Aminoglycoside 3'-phosphotransferase
3236	3236	0.4	Hypothetical
3238	3238	0.4	Hypothetical
3239	fliY	0.4	Secreted Protein
3240	gluA	0.3	Glutamate transport ATP-binding protein GluA
3241	3241	0.4	Acetyltransferase
3242	hipO	0.3	Hippurate hydrolase
3243	moxC	0.3	Putative monooxygenase moxC
3244	ytmO	0.3	Uncharacterized protein ytmO
3245	3245	0.3	Hypothetical
3246	yhbW	0.4	Monooxygenase
3247	msuE	0.3	FMN reductase
3248	irgA	0.3	Iron-regulated outer membrane virulence protein
3249	glxA	0.5	HTH-type transcriptional regulator glxA
3250	3250	0.4	Hypothetical Protein 3250
3251	hbdH1	0.3	D-beta-hydroxybutyrate dehydrogenase

3252	hchA	0.3	Chaperone protein hchA
3253	yafC	0.5	Uncharacterized HTH-type transcriptional regulator HI_1364
3254	guaA	0.5	GMP synthase [glutamine-hydrolyzing]
3257	kefBC	0.4	Glutathione-regulated potassium-efflux system protein
3258	moeB	0.6	Probable adenylyltransferase HVO_0558
3273	3273	0.5	Hypothetical Protein 3273
3274	ihfB	0.4	Integration host factor subunit beta
3283	nolG	0.3	Nodulation protein nolG
3284	nolG	0.4	Nodulation protein nolG
3285	3285	0.5	Multidrug resistance protein mdtA
3290	yeiH	0.4	UPF0324 membrane protein plu2856
3291	3291	0.3	Hypothetical Protein 3291
3292	dbpA	0.5	ATP-independent RNA helicase dbpA
3296	pcaC	0.5	4-carboxymuconolactone decarboxylase
3299	pcaG	0.5	Protocatechuate 3,4-dioxygenase alpha chain
3300	pcaH	0.4	Protocatechuate 3,4-dioxygenase beta chain
3303	pcaI	0.5	3-oxoadipate CoA-transferase subunit A
3306	oprB	0.6	Porin B
3307	quiC	0.5	3-dehydroshikimate dehydratase
3312	rluA	0.4	Uncharacterized RNA pseudouridine synthase slr1592
3313	3313	0.5	Hypothetical Protein 3313
3314	yaeQ	0.5	Uncharacterized protein yaeQ
3317	srpR	0.6	HTH-type transcriptional regulator srpR
3318	3318	0.4	Facilitator Superfamily Protein
3319	ytbD	0.4	Uncharacterized MFS-type transporter ytbD
3321	insF1	0.3	Insertion element IS600 uncharacterized 31 kDa protein
3333	yigZ	0.6	IMPACT family member in pol 5'region
3340	3340	0.4	Hypothetical Protein 3340
3341	grpE	0.3	Protein grpE
3345	3345	0.4	Secreted Protein
3346	3346	0.2	Hypothetical Protein 3346
3353	3353	0.5	Hypothetical Protein 3353
3354	3354	0.5	Hypothetical
3355	ybeQ	0.6	Uncharacterized protein ybeQ
3357	3357	0.3	Acetyltransferase
3358	3358	0.2	Hypothetical
3359	3359	0.3	Hypothetical
3360	yoaA	0.4	Uncharacterized N-acetyltransferase YoaA

3361	3361	0.5	Hypothetical Protein 3361
3364	3364	0.4	Hypothetical
3367	3367	0.5	Hypothetical
3368	3368	0.5	Hypothetical Protein 3368
3370	ucpA	0.5	Monensin polyketide synthase putative ketoacyl reductase
3371	bepE	0.3	Efflux pump membrane transporter BepE
3372	bepF	0.4	Efflux pump periplasmic linker BepF
3373	3373	0.4	Short-Chain Dehydrogenase/Reductase SDR
3374	3374	0.4	Hypothetical Protein 3374
3376	yxaH	0.2	Uncharacterized protein yxaH
3377	oar	0.3	Protein oar
3382	3382	0.5	Hypothetical Protein 3382
3383	algU	0.4	RNA polymerase sigma-H factor
3384	3384	0.3	Hypothetical Protein 3384
3385	sodA	0.4	Superoxide dismutase [Mn]
3386	ntaB	0.6	Nitrilotriacetate monooxygenase component B
3387	3387	0.6	TPR repeat-containing protein PA0015
3388	3388	0.4	Hypothetical Protein 3388
3389	3389	0.4	Hypothetical Protein 3389
3390	ppa	0.4	Inorganic pyrophosphatase
3391	3391	0.5	Metallophosphoesterase
3392	3392	0.4	Hypothetical
3393	cirA	0.4	NB-Dependent Receptor
3401	rsmD	0.5	Ribosomal RNA small subunit methyltransferase D
3402	htpG	0.3	Chaperone protein htpG
3424	gacA	0.5	Response regulator gacA
3427	regB	0.6	Sensor histidine kinase regB
3435	gtr	0.5	Glucose transport protein
3442	ylaK	0.5	Uncharacterized protein ylaK
3444	3444	0.4	Hypothetical
3445	thiDE	0.5	Bifunctional protein thiED
3447	3447	0.5	Hypothetical
3451	fpvA	0.4	Ferripyoverdine receptor
3466	3466	0.4	Hypothetical Protein 3466
3476	3476	0.5	Hypothetical Protein 3476
3482	3482	0.4	Hypothetical Protein 3482
3486	parE	0.4	DNA topoisomerase 4 subunit B
3497	fbp	0.5	FK506-binding protein

3502	ycgF	0.4	Hypothetical
3503	3503	0.4	GumN Protein
3504	3504	0.5	Hypothetical
3505	3505	0.6	Hypothetical
3507	3507	0.4	Hypothetical Protein 3507
3509	hemN	0.2	Oxygen-independent coproporphyrinogen-III oxidase
3510	ctb	0.4	Group 3 truncated hemoglobin ctb
3511	3511	0.3	Hypothetical
3512	rdxA	0.3	Protein rdxA
3513	3513	0.5	Hypothetical
3515	ycgF	0.5	Hypothetical
3516	3516	0.3	Conserved Hypothetical Protein
3517	3517	0.3	Digeranylgeranylglycerophospholipid reductase
3518	3518	0.3	Hypothetical
3519	3519	0.4	Hypothetical Protein 3519
3528	comA	0.5	Competence protein ComA
3530	3530	0.4	Fimbrial Biogenesis Protein
3531	ppdD	0.3	Fimbrial Protein Pilin
3532	3532	0.3	Type 4 Fimbrial Biogenesis
3533	3533	0.2	PilX Protein
3534	3534	0.3	Hypothetical
3535	3535	0.3	Pre-Pilin Leader Sequence
3536	3536	0.2	Pre-Pilin Like Leader Sequence
3550	petC	0.5	Cytochrome c1
3551	petB	0.3	Cytochrome b
3552	petA	0.4	Ubiquinol-cytochrome c reductase iron-sulfur subunit
3553	yjbJ	0.3	Putative murein lytic transglycosylase yjbJ
3555	ybhS	0.4	Inner membrane transport permease ybhS
3556	yhiH	0.5	Uncharacterized ABC transporter ATP-binding protein YhiH
3558	3558	0.4	Fatty Acid Desaturase
3559	3559	0.5	Phosphoesterase PA-Phosphatase
3564	3564	0.2	Hypothetical Protein 3564
3565	3565	0.2	Hypothetical
3566	3566	0.2	Hypothetical
3567	3567	0.2	Phospholipase D
3568	3568	0.5	Rhs Element Vgr Protein
3572	3572	0.6	Hypothetical
3584	3584	0.3	Hypothetical

3585	3585	0.3	Hypothetical
3586	3586	0.3	Hypothetical
3587	3587	0.2	Hypothetical
3588	3588	0.2	Hypothetical Protein 3588
3589	3589	0.3	Rhs Element Vgr Protein
3590	pleD	0.5	Response regulator PleD
3591	3591	0.2	Bacteriohemerythrin
3592	yhjE	0.5	Inner membrane metabolite transport protein yhjE
3607	3607	0.6	Hypothetical
3614	nth	0.5	Endonuclease III
3615	3615	0.3	Uncharacterized protein CC_0911
3616	3616	0.5	Hypothetical Protein 3616
3617	3617	0.5	Hypothetical
3618	yijE	0.3	Hypothetical Protein yijE
3619	3619	0.2	2OG-Fe(II) Oxygenase
3620	moeB	0.2	Sulfur carrier protein moaD adenylyltransferase
3621	pstS	0.3	Phosphate-binding protein pstS
3622	pstS	0.3	Phosphate-binding protein pstS
3623	pstC	0.4	Phosphate transport system permease protein pstC
3624	pstA	0.4	Phosphate transport system permease protein pstA
3625	pstB	0.4	Phosphate import ATP-binding protein PstB
3626	phoU	0.4	Phosphate transport system protein phoU
3627	3627	0.6	Hypothetical Protein 3627
3628	3628	0.5	UPF0276 protein PSEEN3355
3629	3629	0.5	Hypothetical
3630	3630	0.5	Uncharacterized protein HI_1602
3635	macA	0.6	Macrolide-specific efflux protein macA
3636	macB	0.5	Macrolide export ATP-binding/permease protein MacB
3642	emrB	0.6	Multidrug resistance protein B
3649	acyII	0.5	Penicillin acylase 2
3650	napA	0.4	Na(+)/H(+) antiporter
3651	asnB	0.5	Asparagine synthetase B [glutamine-hydrolyzing]
3652	ybeQ	0.5	Hypothetical
3653	3653	0.4	Hypothetical
3667	3667	0.4	Hypothetical
3681	dnaE	0.6	DNA polymerase III subunit alpha
3682	3682	0.5	Hypothetical
3685	insF1	0.3	Insertion element IS600 uncharacterized 31 kDa protein

3686	3686	0.4	UPF0093 membrane protein HP_1484
3687	3687	0.5	Hypothetical
3690	ywzG	0.5	Putative DNA-binding protein ywzG
3691	3691	0.5	Peptidyl-Prolyl Cis-Trans Isomerase
3692	yijE	0.4	Hypothetical
3693	gcvA	0.4	Glycine cleavage system transcriptional activator
3696	3696	0.5	Hypothetical
3700	3700	0.5	Glyoxalase/Bleomycin Resistance Protein/Dioxygenase
3701	yecE	0.5	Hypothetical
3702	3702	0.6	Hypothetical
3704	ydaM	0.5	Uncharacterized protein ydaM
3705	rluF	0.5	Ribosomal large subunit pseudouridine synthase F
3714	alkA	0.6	Probable bifunctional transcriptional activator/DNA repair enzyme AlkA
3715	ogt	0.6	Methylated-DNA--protein-cysteine methyltransferase
3716	3716	0.5	Phosphodiesterase I
3719	btuB	0.5	Probable tonB-dependent receptor NMB0964
3720	3720	0.3	MarR Family Transcriptional Regulator
3721	ybfB	0.4	Uncharacterized MFS-type transporter ybfB
3722	3722	0.5	Hypothetical Protein 3722
3729	3729	0.5	Aminopeptidase S
3730	3730	0.5	Hypothetical
3731	creD	0.3	Inner membrane protein CreD
3732	creC	0.4	Sensor protein CreC
3733	creB	0.5	Transcriptional regulatory protein CreB
3741	czcD	0.6	Cation efflux system protein CzcD
3742	dosC	0.5	Diguanylate cyclase DosC
3747	3747	0.5	Myosin-Cross-Reactive Antigen
3754	3754	0.4	BaRNAse Inhibitor
3755	3755	0.2	Hypothetical Protein 3755
3757	3757	0.6	Hypothetical
3759	3759	0.5	Glycosyl Hydrolase Family 5 Protein
3778	cyoD	0.5	Cytochrome o ubiquinol oxidase protein CyoD
3779	cyoC	0.4	Cytochrome o ubiquinol oxidase subunit 3
3780	cyoB	0.4	Ubiquinol oxidase subunit 1
3787	3787	0.6	Hypothetical
3788	3788	0.2	Hypothetical
3796	mviN	0.6	Virulence factor mviN homolog
3808	rfaY	0.6	Probable RNA polymerase sigma factor rfaY

3809	3809	0.6	Hypothetical
3811	3811	0.3	Hypothetical Protein 3811
3820	yfjP	0.5	Putative DNA-3-methyladenine glycosylase yfjP
3821	3821	0.4	Hypothetical
3823	yfiK	0.6	Uncharacterized transcriptional regulatory protein yfiK
3824	yfiJ	0.6	Sensor histidine kinase yfiJ
3830	3830	0.4	Hypothetical
3832	3832	0.4	Putative oligopeptide transporter HI_0561
3833	3833	0.4	Hypothetical
3834	yclF	0.4	Uncharacterized transporter yclF
3837	iciA	0.5	Uncharacterized HTH-type transcriptional regulator Rv1985c/MT2039
3838	3838	0.3	Hypothetical
3839	3839	0.3	Hypothetical
3840	3840	0.3	Hypothetical
3841	3841	0.6	Hypothetical
3844	3844	0.4	Hypothetical Protein 3844
3845	fpvA	0.3	Ferripyoverdine receptor
3846	3846	0.4	Malonate Decarboxylase Alpha Subunit
3853	3853	0.4	Dicarboxylate Carrier MatC Domain-Containing Protein
3856	3856	0.4	Hypothetical
3857	mutM	0.6	Formamidopyrimidine-DNA glycosylase
3860	3860	0.3	Hypothetical Protein 3860
3863	3863	0.3	Hypothetical
3864	3864	0.3	NB-Dependent Receptor
3865	fecR	0.4	Anti-FecI Sigma Factor FecR
3866	fecI	0.4	RNA Polymerase Sigma Factor
3867	sdpR	0.4	Transcriptional repressor sdpR
3872	3872	0.6	Hypothetical
3876	3876	0.6	Uncharacterized protein Rv0906/MT0929
3878	3878	0.6	Hypothetical
3880	3880	0.5	Hypothetical
3881	3881	0.3	Facilitator Transporter
3890	3890	0.3	Gluconate 2-dehydrogenase flavoprotein
3891	ygbM	0.3	Uncharacterized 28.3 kDa protein in gbd 5'region
3893	3893	0.5	RTX xin-Activating Protein C
3894	4488	0.3	Beta-lactamase Classs C
3895	lktD	0.3	Leukotoxin secretion protein D
3896	lktB	0.3	Leukotoxin translocation ATP-binding protein LktB

3897	celR	0.5	HTH-type transcriptional regulator CelR
3898	3898	0.5	Hypothetical
3899	yfiL	0.3	Uncharacterized ABC transporter ATP-binding protein YfiL
3900	3900	0.3	Hypothetical
3903	3903	0.4	Hypothetical
3904	malS	0.4	Cyclomaltodextrin glucanotransferase
3905	3905	0.5	Sugar Transporter
3906	3906	0.5	Hypothetical
3907	3907	0.3	Alpha-Glucosidase
3908	3908	0.3	NB-Dependent Receptor
3909	aglA	0.4	Probable alpha-glucosidase
3912	3912	0.5	Hypothetical
3918	3918	0.1	Histone-Like Protein
3931	bedB	0.6	Benzene 1,2-dioxygenase system ferredoxin subunit
3934	ybiX	0.6	PKHD-type hydroxylase Smlt1146
3936	cirA	0.5	Colicin I receptor
3937	piv	0.3	Pilin gene-inverting protein
3938	3938	0.5	Hypothetical
3939	3939	0.4	Hypothetical
3940	3940	0.6	Hypothetical
3951	perM	0.6	UPF0118 membrane protein RP630
3961	bolA	0.6	BolA Family Protein
3974	ptsI	0.5	Phosphoenolpyruvate-protein phosphotransferase
3982	yhgE	0.6	Uncharacterized protein yhgE
3983	tag	0.5	DNA-3-methyladenine glycosylase 1
3984	3984	0.4	Hypothetical
3985	3985	0.5	Hypothetical
3986	3986	0.4	Hypothetical
3987	cpo	0.4	Non-heme chloroperoxidase
3988	yccK	0.4	Uncharacterized oxidoreductase YccK
3989	3989	0.3	Hypothetical
3990	pilT	0.3	Twitching mobility protein
3991	pilT	0.4	Twitching mobility protein
3992	yggS	0.6	UPF0001 protein PM0112
3995	fabG	0.3	Short Chain Dehydrogenase
3996	soxR	0.4	Redox-sensitive transcriptional activator soxR
3998	3998	0.6	Hypothetical
4002	4002	0.3	NB-Dependent Siderophore Receptor

4003	yebQ	0.4	Uncharacterized transporter yebQ
4005	4005	0.5	Hypothetical
4006	4006	0.4	Hypothetical Protein 4006
4008	fabG	0.5	Putative short-chain type dehydrogenase/reductase Rv0148
4011	4011	0.2	Conserved Hypothetical Protein
4012	4012	0.2	Hypothetical Protein 4012
4013	4013	0.3	Hypothetical Protein 4013
4014	ybjQ	0.2	UPF0145 protein CV_4322
4015	4015	0.3	Hypothetical Protein 4015
4016	yehP	0.4	Uncharacterized protein yehP
4018	yehL	0.5	Uncharacterized protein yehL
4019	4019	0.5	Hypothetical
4021	4021	0.5	Hypothetical
4022	4022	0.6	Type IV Pilus Assembly PilZ
4037	yeaC	0.4	Uncharacterized protein yeaC
4038	4038	0.4	Hypothetical
4049	4049	0.5	Hypothetical
4068	4068	0.5	Hypothetical Protein 4068
4070	4070	0.6	Hypothetical
4072	yddQ	0.4	Uncharacterized isochorismatase family protein yddQ
4077	phoB	0.6	Phosphate regulon transcriptional regulatory protein phoB
4081	btuB	0.3	Vitamin B12 transporter BtuB
4082	mgtA	0.4	GDP-mannose-dependent alpha-mannosyltransferase
4083	pgpB	0.4	Phosphoesterase PA-Phosphatase Related Protein
4086	4086	0.4	Peptidyl-Asp metalloendopeptidase
4087	4087	0.5	Hypothetical
4096	4096	0.6	Hypothetical
4098	4098	0.5	Hypothetical
4103	4103	0.5	Uncharacterized protein HI_0522
4104	fadH	0.4	2,4-dienoyl-CoA reductase [NADPH]
4108	4108	0.3	Hypothetical
4109	4109	0.3	Hypothetical Protein 4109
4110	prpE	0.4	Propionate--CoA ligase
4113	typA	0.4	GTP-binding protein TypA/BipA
4114	4114	0.4	Hypothetical
4118	cirA	0.4	NB-Dependent Receptor
4119	dsbB	0.6	Disulfide bond formation protein B
4162	btuB	0.5	Vitamin B12 transporter BtuB

4163	yurR	0.3	Uncharacterized oxidoreductase YurR
4164	4164	0.3	Hypothetical Protein 4164
4165	4165	0.3	Hypothetical Protein 4165
4181	4181	0.6	Hypothetical
4183	piv	0.3	Pilin gene-inverting protein
4184	4184	0.5	Extracellular protease
4206	ahpF	0.5	Alkyl hydroperoxide reductase subunit F
4211	4211	0.5	Hypothetical
4228	4228	0.5	Hypothetical Protein 4228
4240	tesA	0.5	Esterase TesA
4250	ynbD	0.4	Uncharacterized protein ynbD
4251	4251	0.4	Hypothetical Protein 4251
4254	4254	0.5	Hypothetical Protein 4254
4256	4256	0.5	Methyltransferase
4257	4257	0.5	Hypothetical
4258	4258	0.4	Uncharacterized protein MJ1222
4259	4259	0.5	Membrane-Bound Metal-Dependent Hydrolase
4260	metK	0.3	S-adenosylmethionine synthase
4263	yuxL	0.5	Uncharacterized peptidase yuxL
4265	yfbP	0.5	Uncharacterized protein yfbP
4266	ybaK	0.5	Cys-tRNA(Pro)/Cys-tRNA(Cys) deacylase ybaK
4267	4267	0.4	Hypothetical
4268	tonB	0.4	Protein tonB
4269	4269	0.6	Hypothetical
4294	4294	0.5	Hypothetical
4300	4300	0.4	Fimbrial Protein
4301	4301	0.5	Fimbrial Subunit
4302	mrkC	0.4	Outer membrane usher protein mrkC
4303	fimI	0.4	Fimbrial Protein FimI
4304	ecpD	0.5	Chaperone protein ecpD
4311	4311	0.5	Hypothetical
4314	4314	0.5	Hypothetical Protein 4314
4318	4318	0.6	Hypothetical
4319	yjjK	0.6	Uncharacterized ABC transporter ATP-binding protein YjjK
4320	omp31	0.3	31 kDa outer-membrane immunogenic protein
4321	purP	0.4	Probable adenine permease PurP
4334	pncB	0.5	Nicotinate phosphoribosyltransferase
4336	4336	0.6	Hypothetical

4345	xpsG	0.6	General secretion pathway protein G
4351	chiC	0.1	Chitinase C
4352	dsbC	0.6	Thiol:disulfide interchange protein DsbC
4365	4365	0.4	Hypothetical
4369	4369	0.6	Hypothetical
4386	ybfF	0.4	Uncharacterized glycosyltransferase slr1943
4387	4387	0.3	GCN5-Like N-Acetyltransferase
4388	4388	0.3	Hypothetical
4389	rffA	0.3	Lipopolysaccharide biosynthesis protein rffA
4390	4390	0.3	Hypothetical Protein 4390
4391	4391	0.6	Glycosyl Transferase Family Protein
4392	4392	0.3	Glycosyl Transferase Family Protein
4393	4393	0.3	Uncharacterized protein y4gI
4394	catB2	0.2	Chloramphenicol acetyltransferase
4395	abcA	0.3	ABC transporter protein AbcA
4396	rfbA	0.3	O-antigen export system permease protein rfbA
4402	pilT	0.6	Twitching mobility protein
4406	4406	0.3	Peptidase
4407	btuB	0.4	Vitamin B12 transporter BtuB
4408	queD	0.5	6-carboxy-5,6,7,8-tetrahydropterin synthase
4410	4410	0.3	Hypothetical
4412	yclJ	0.5	Uncharacterized transcriptional regulatory protein yclJ
4413	msrB	0.4	Peptide methionine sulfoxide reductase MsrB
4414	dipZ	0.6	Protein dipZ
4415	msrA2	0.4	Peptide methionine sulfoxide reductase MsrA 2
4417	bioD	0.6	ATP-dependent dethiobiotin synthetase BioD
4421	btuB	0.5	Vitamin B12 transporter BtuB
4423	ygiP	0.4	Uncharacterized HTH-type transcriptional regulator HI_1364
4424	ywnB	0.4	Uncharacterized protein ywnB
4425	4425	0.3	Beta-Lactamase Domain-Containing Protein
4426	dinB	0.4	DNA polymerase IV
4440	4440	0.5	Hypothetical
4441	msrB	0.3	Peptide methionine sulfoxide reductase MsrB
4442	motA	0.4	Motility protein A
4443	motB	0.4	Motility protein B
4450	ygiD	0.5	Uncharacterized protein ygiD
4451	4451	0.6	DoxX Family Protein
4453	ykoW	0.5	Signaling protein ykoW

4455	4455	0.6	Hypothetical
4457	4457	0.4	Hypothetical
4458	yehT	0.4	Uncharacterized response regulatory protein VPA0021
4459	yehU	0.4	Inner membrane protein yehU
4461	4461	0.6	Uncharacterized protein R00370
4462	4462	0.4	Glyoxalase/Bleomycin Resistance Protein/Dioxygenase
4463	phnB	0.3	Protein phnB
4464	4464	0.3	Hypothetical
4465	4465	0.5	Uncharacterized protein R00369
4466	pknD	0.5	Serine/threonine-protein kinase pknD
4467	4467	0.5	Hypothetical
4468	yjbJ	0.4	Putative murein lytic transglycosylase yjbJ
4469	cycA	0.3	D-serine/D-alanine/glycine transporter
4470	ylaB	0.4	Uncharacterized protein YlaB
4471	4471	0.5	Polysaccharide Deacetylase
4472	sotB	0.3	Probable sugar efflux transporter
4473	yafC	0.5	Uncharacterized HTH-type transcriptional regulator yafC
4475	yciC	0.4	Putative metal chaperone YciC
4476	4476	0.5	Hypothetical
4478	4478	0.6	Hypothetical
4479	yafM	0.6	Uncharacterized protein yafM
4480	4480	0.3	Hypothetical Protein 4480
4481	4481	0.5	Hypothetical Protein 4481
4482	4482	0.5	Uncharacterized protein MJ1233
4486	4486	0.6	CopY Family Transcriptional Regulator
4488	dap	0.4	D-aminopeptidase
4490	4490	0.4	Hypothetical Protein 4490
4491	4491	0.3	Hypothetical Protein 4491
4492	4492	0.3	Hypothetical Protein 4492
4493	4493	0.3	Hypothetical Protein 4493
4494	yncE	0.5	Uncharacterized protein YncE
4495	4495	0.3	Hypothetical Protein 4495
4496	4496	0.3	Hypothetical
4497	4497	0.4	Paar Motif Family Protein
4498	4498	0.3	Hypothetical
4499	4499	0.2	Hypothetical
4500	4500	0.3	Hypothetical
4501	4501	0.3	Hypothetical

4502	4502	0.2	Hypothetical
4503	4503	0.2	Hypothetical Protein 4503
4504	4504	0.5	Hypothetical
4505	4505	0.4	Hypothetical Protein 4505
4506	4506	0.6	Hypothetical Protein 4506
4507	4507	0.5	Transcriptional Factor
4508	4508	0.4	Hypothetical Protein 4508
4509	4509	0.4	Hypothetical
4511	4511	0.6	Hypothetical Protein 4511
4512	4512	0.2	Hypothetical Protein 4512
4513	4513	0.2	Hypothetical
4514	4514	0.2	Hypothetical Protein 4514
4515	mcrB	0.2	5-methylcytosine-specific restriction enzyme B
4516	4516	0.2	Hypothetical
4517	4517	0.3	Hypothetical
4521	4521	0.5	Hypothetical Protein 4521
4524	yceI	0.4	UPF0312 protein PFLU_5725
4525	ycdI	0.5	Putative NADH dehydrogenase/NAD(P)H nitroreductase Smal_0358
4529	4529	0.5	Hypothetical
4532	glnE	0.5	Glutamate-ammonia-ligase adenylyltransferase
4535	4535	0.5	Hypothetical
4539	4539	0.4	Hypothetical
4540	vanX	0.4	D-alanyl-D-alanine dipeptidase
4541	pbp	0.5	Penicillin-binding protein 4
4542	oar	0.4	Protein oar
4549	yhdG	0.6	Uncharacterized amino acid permease YhdG
4563	yfeH	0.5	Uncharacterized protein PA2026
4564	4564	0.2	Hypothetical
4565	4565	0.2	Hypothetical
4566	4566	0.4	Hypothetical
4567	cyoE	0.5	Protoheme IX farnesyltransferase
4570	4570	0.4	Hypothetical
4571	4571	0.2	Hypothetical
4572	ctaE	0.4	Cytochrome c oxidase subunit 3
4573	ctaG	0.5	Cytochrome c oxidase assembly protein CtaG
4575	ctaD	0.5	Probable cytochrome c oxidase subunit 1
4577	putA	0.3	Bifunctional protein putA
4578	4578	0.3	Hypothetical

4580	4580	0.4	Rhomboid Family Protein
4582	envC	0.5	Murein hydrolase activator EnvC
4585	yafM	0.2	Hypothetical
4586	tyrS	0.5	Tyrosyl-tRNA synthetase
4591	exoA	0.6	Exodeoxyribonuclease
4594	4594	0.3	Hypothetical Protein 4594
4595	kdpA	0.3	Potassium-transporting ATPase A chain
4596	kdpB	0.3	Potassium-transporting ATPase B chain
4597	kdpC	0.2	Potassium-transporting ATPase C chain
4599	kdpE	0.5	Transcriptional regulatory protein KdpE
4603	4603	0.3	Uncharacterized protein NMB0459
4604	radC	0.2	UPF0758 protein XCC3860
4607	4607	0.3	Uncharacterized HTH-type transcriptional regulator in mcrB 3'region
4608	4608	0.3	Glyoxalase/Bleomycin Resistance Protein/Dioxygenase
4609	sdh	0.6	Serine 3-dehydrogenase
4610	4610	0.2	Hypothetical
4612	gltC	0.6	HTH-type transcriptional regulator gltC
4614	4614	0.5	Hypothetical
4617	4617	0.3	NB-Dependent Receptor
4618	aly	0.4	Alginate lyase
4619	4619	0.5	Heparinase II/III Family Protein
4620	exuT	0.5	Hexuronate transporter
4621	fabG	0.5	3-oxoacyl-[acyl-carrier-protein] reductase FabG
4622	4622	0.5	Secreted Protein
4626	4626	0.3	Xylose Isomerase Domain-Containing Protein
4627	ycdJ	0.4	Soluble epoxide hydrolase
4628	lip2	0.4	Lipase 2
4630	ybaA	0.3	Uncharacterized protein ybaA
4633	4633	0.2	Hypothetical Protein 4633
4634	4634	0.5	Hypothetical
4635	4635	0.3	Hypothetical Protein 4635
4636	4636	0.3	Hypothetical
4642	4642	0.4	Hypothetical Protein 4642
4654	desR	0.4	Transcriptional regulatory protein desR
4655	yvfT	0.4	Sensor histidine kinase yvfT
4656	yvfS	0.5	Putative transport permease yvfS
4657	yvfR	0.4	Uncharacterized ABC transporter ATP-binding protein YvfR
4658	macA	0.5	Probable macrolide-specific efflux protein macA

4659	yknY	0.4	Uncharacterized ABC transporter ATP-binding protein YknY
4660	4660	0.3	Uncharacterized ABC transporter permease MJ1507
4661	macB	0.5	Macrolide export ATP-binding/permease protein MacB
4662	btuB	0.3	Vitamin B12 transporter BtuB
4663	piv	0.2	Pilin gene-inverting protein
4664	yojE	0.3	Uncharacterized transporter yojE
4670	4670	0.6	Hypothetical
4672	4672	0.5	Hypothetical
4687	4687	0.5	Hypothetical Protein 4687
4689	ohr	0.5	Organic hydroperoxide resistance protein
4698	blc	0.4	Outer membrane lipoprotein blc
4699	cfa	0.4	Cyclopropane-fatty-acyl-phospholipid synthase
4700	4700	0.3	Hypothetical
4701	4701	0.6	Hypothetical
4702	cfa	0.4	Cyclopropane-fatty-acyl-phospholipid synthase
4703	4703	0.4	Hypothetical
4704	4704	0.5	Amine Oxidase
4705	4705	0.3	Stearoyl-CoA 9-Desaturase
4708	4708	0.4	Hypothetical
4710	nadR	0.6	Trifunctional NAD biosynthesis/regulator protein NadR
4719	4719	0.3	Hypothetical
4720	pdhA	0.4	Pyruvate dehydrogenase E1 component
4723	cobB	0.5	NAD-dependent deacetylase
4724	yciA	0.4	Uncharacterized acyl-CoA thioester hydrolase ZMO0511
4725	4725	0.3	Hypothetical
4726	4726	0.4	Hypothetical
4743	4743	0.5	Hypothetical
4746	4746	0.4	Hypothetical
4747	4747	0.3	Hypothetical
4754	lepB	0.6	Signal peptidase I
4755	4755	0.4	Hypothetical
4756	4756	0.3	Hypothetical
4757	dcp	0.4	Peptidyl-dipeptidase dcp
4768	ykgJ	0.4	Uncharacterized protein ykgJ
4769	dhaAF	0.3	Haloalkane dehalogenase
4773	4773	0.5	Hypothetical
4781	rcsB	0.5	Capsular synthesis regulator component B
4783	4783	0.6	Aminoglycoside Phosphotransferase

4788	plcN	0.6	Non-hemolytic phospholipase C
4789	fhuA	0.4	NB-Dependent Receptor
4790	4790	0.4	Phospholipase/Carboxylesterase
4791	nolG	0.6	Nodulation protein nolG
4792	4792	0.5	Multidrug resistance protein
4793	tetR	0.5	Tetracycline repressor protein class A from transposon 1721
4794	4794	0.4	Hypothetical
4795	ycgF	0.3	Blue light- and temperature-regulated antirepressor YcgF
4796	4796	0.2	Hypothetical Protein 4796
4797	4797	0.2	Beta-Lactamase Domain-Containing Protein
4798	4798	0.2	Hypothetical
4799	4799	0.2	Hypothetical
4800	gcvA	0.3	Glycine cleavage system transcriptional activator
4801	4801	0.4	Hypothetical
4802	4802	0.4	Hypothetical
4803	4803	0.3	Hypothetical
4804	4804	0.3	Hypothetical
4805	4805	0.5	Hypothetical Protein 4805
4806	bioC	0.4	Uncharacterized protein HI_0912
4807	rcsC	0.4	Sensor kinase protein RcsC
4808	yddU	0.3	Uncharacterized signaling protein CC_0091
4809	rcsB	0.3	Capsular synthesis regulator component B
4810	rcsC	0.4	Sensor kinase protein RcsC
4811	rcsB	0.3	Capsular synthesis regulator component B
4817	4817	0.6	Hypothetical
4818	yiiD	0.4	Hypothetical
4819	hemD	0.5	Uroporphyrinogen-III Synthase
4828	4828	0.3	Hypothetical Protein 4828
4829	4829	0.2	Hypothetical
4830	4830	0.2	Hypothetical Protein 4830
4831	4831	0.3	Glyoxalase/Bleomycin Resistance Protein/Dioxygenase
4832	trxB	0.6	Thioredoxin reductase
4833	mltA	0.4	Membrane-bound lytic murein transglycosylase A
4834	amiD	0.5	N-acetylmuramoyl-L-alanine amidase AmiD
4835	amtB	0.4	Ammonia channel
4836	glnB	0.3	Nitrogen regulatory protein P-II
4837	4837	0.4	Hypothetical Protein 4837
4838	glnA	0.4	Glutamine synthetase

4839	uppP	0.5	Undecaprenyl-diphosphatase
4845	ydiI	0.5	Putative esterase PA1618
4854	4854	0.4	Transcriptional Regulator
4855	hipA	0.4	Putative kinase Y4mE
4856	4856	0.5	Hypothetical
4857	tldD	0.6	Uncharacterized protein MJ0996
4858	tldD	0.4	Uncharacterized protein MTH_856
4859	pmbA	0.5	Peptidase
4860	4860	0.4	Hypothetical
4861	yeaC	0.4	Uncharacterized protein yeaC
4866	4866	0.5	Hypothetical Protein 4866
4869	4869	0.5	Hypothetical
4890	4890	0.5	Hypothetical
4891	4891	0.4	Trypsin
4895	4895	0.6	Acetyltransferase
4902	4902	0.5	Hypothetical
4903	4903	0.4	Hypothetical
4907	4907	0.5	Hypothetical
4908	phoN	0.5	Non-specific acid phosphatase
4912	4912	0.3	Hypothetical
4913	4913	0.2	Hypothetical
4914	4914	0.2	Hypothetical Protein 4914
4920	kduD	0.5	2-dehydro-3-deoxy-D-gluconate 5-dehydrogenase
4921	kduI	0.4	4-deoxy-L-threo-5-hexosulose-uronate ketol-isomerase
4922	exuR	0.5	Probable HTH-type transcriptional repressor exuR
4923	yagH	0.3	Putative beta-xylosidase
4924	4924	0.4	Pectate Lyase
4925	yiaN	0.4	Uncharacterized protein y4mL
4926	4926	0.3	C4-Dicarboxylate Transport Small Permease Component
4927	yiiZ	0.3	Uncharacterized protein yiiZ
4928	4928	0.3	2-Keto-4-Pentenoate Hydratase
4929	xynB	0.4	Endo-1,4-beta-xylanase B
4930	pnbA	0.4	Para-nitrobenzyl esterase
4931	yesY	0.4	Probable rhamnogalacturonan acetylesterase yesY
4934	4934	0.5	Hypothetical
4935	cirA	0.3	NB-Dependent Receptor
4936	cirA	0.3	NB-Dependent Receptor
4937	kdgK	0.4	2-dehydro-3-deoxygluconokinase

4938	eda	0.4	KHG/KDPG aldolase
4939	chbR	0.4	Cupin 2 Domain-Containing Protein
4942	adhA	0.4	Alcohol dehydrogenase
4943	4943	0.6	Hypothetical
4946	4946	0.6	Probable DNA repair protein COXBURSA331_A2134
4951	fbp	0.4	Fructose-1,6-bisphosphatase class 1
4952	4952	0.3	Hypothetical
4953	4953	0.4	Hypothetical Protein 4953
4954	ybhF	0.4	Putative ABC transporter ATP-binding protein MK0182
4955	4955	0.3	Hypothetical
4956	4956	0.3	Hypothetical Protein 4956
4957	4957	0.3	Hypothetical Protein 4957
4958	macB	0.3	Macrolide export ATP-binding/permease protein MacB
4959	yknY	0.4	Uncharacterized ABC transporter ATP-binding protein YknY
4960	macA	0.3	Macrolide-specific efflux protein macA
4961	4961	0.3	Hypothetical
4962	4962	0.3	Transposase
4963	4963	0.3	Hypothetical Protein 4963
4964	4964	0.4	Hypothetical Protein 4964
4968	cls2	0.4	Cardiolipin synthase 2
4978	4978	0.3	Hypothetical Protein 4978
4986	4986	0.3	Hypothetical
4987	glpQ	0.5	Glycerophosphoryl diester phosphodiesterase
4990	alx	0.5	Inner membrane protein alx
4992	4992	0.6	Uncharacterized protein in pqqA 5'region
4993	yhdG	0.5	Uncharacterized amino acid permease YhdG
4994	4994	0.4	Hypothetical
4995	4995	0.5	Hypothetical
4996	yccK	0.4	Uncharacterized oxidoreductase YccK
4998	4998	0.5	Hypothetical Protein 4998
4999	yeaS	0.4	Uncharacterized membrane protein PA4757
5000	yafC	0.5	Uncharacterized HTH-type transcriptional regulator yafC
5001	qor	0.4	Zinc-type alcohol dehydrogenase-like protein SERP1785
5002	nfnB	0.5	Oxygen-insensitive NAD(P)H nitroreductase
5003	yliJ	0.5	Uncharacterized GST-like protein yliJ
5020	5020	0.4	Hypothetical
5024	5024	0.5	Hypothetical
5025	5025	0.5	Hypothetical

5026	yfcA	0.4	UPF0721 transmembrane protein ORF9
5027	ychJ	0.4	UPF0225 protein XCC4159
5028	pleD	0.5	Response regulator PleD
5033	ydcO	0.6	Inner membrane protein ydcO
