# Supplementary material for: Root-microbe systems: the effect and mode of interaction of Stress Protecting Agent (SPA) Stenotrophomonas rhizophila DSM14405T
Source: Front Plant Sci. 2013 May 14;4:141. doi: 10.3389/fpls.2013.00141 (PMC3653106; doi:10.3389/fpls.2013.00141)
Supplement: Table S1A — Significantly up-regulated genes in S. rhizophila DSM14405T under salt shock. [file DataSheet1.ZIP › 51567_Berg_Table_S2A.PDF]

Supplementary Table 2a: significantly up-regulated genes in *S. rhizophila* DSM14405T under treatment with root exudates

locus tag	gene	fold change	product
1002	1002	2.3	Hydrolase
1023	1023	1.6	Hypothetical
1029	parB	1.6	Probable chromosome-partitioning protein parB
1037	czcC	3	Cobalt-zinc-cadmium resistance protein CzcC
1039	helA	1.5	Protein HelA
1043	1043	1.5	Uncharacterized glycosyltransferase Rv1524/MT1575
1046	cls	1.8	Cardiolipin synthase
1047	1047	1.5	Hypothetical
1051	1051	1.5	Hypothetical
1053	1053	2	Hypothetical
1074	pcl	1.5	4-coumarate--CoA ligase
1095	yfiQ	1.5	Uncharacterized protein yfiQ
1096	ycdJ	1.6	Alpha/Beta Hydrolase Fold Protein
1104	rtcB	2.6	RNA-splicing ligase RtcB
1105	1105	1.6	Hypothetical Protein 1105
1106	yrpG	1.8	Uncharacterized oxidoreductase YrpG
1110	hrpB	2.2	ATP-dependent RNA helicase hrpB
1112	tatD	1.5	Deoxyribonuclease tatD
1113	1113	1.5	NAD-Dependent Epimerase/Dehydratase
1114	ykpB	1.8	Uncharacterized oxidoreductase ykpB
1118	yedQ	1.5	Cellulose synthesis regulatory protein
1120	natA	1.9	ATP-binding transport protein NatA
1130	1130	1.8	Hypothetical
1132	1132	1.6	Hypothetical
1142	dinG	1.5	Probable ATP-dependent helicase dinG homolog
1144	1144	1.7	Hypothetical
1145	ilvD	1.8	Dihydroxy-acid dehydratase
1146	ygiB	1.5	UPF0441 protein ygiB
1155	yrdA	1.5	Protein YrdA
1156	ycgL	2.2	Uncharacterized protein ycgL
1159	1159	1.5	Hypothetical Protein 1159
1160	psdht	1.8	Phenylserine dehydratase
1161	ygjQ	1.9	Hypothetical
1168	comF	1.9	Competence protein F
1169	1169	1.5	Hypothetical

1170	1170	1.5	Hypothetical
1171	1171	1.7	Hypothetical
1174	nagA	2.4	N-acetylglucosamine-6-phosphate deacetylase
1177	nagK	1.8	N-acetyl-D-glucosamine kinase
1183	1183	22.6	Hypothetical Protein 1183
1184	1184	2.6	Hypothetical
1186	1186	13.1	Anti-FecI Sigma Factor FecR
1187	1187	2.1	Hypothetical
1188	1188	1.7	Hemolysin Activation/Secretion Protein
1189	tolQ	2	Protein tolQ
1190	exbD1	27	Biopolymer transport protein exbD1
1191	1191	2.9	Hypothetical
1198	1198	2	HsdR Family Type I Site-Specific Deoxyribonuclease
1204	1204	1.5	Hypothetical
1210	pld1	1.5	Pyridoxal 4-dehydrogenase
1212	1212	2.3	XRE Family Transcriptional Regulator
1225	1225	1.6	Kinase
1226	1226	1.8	Hypothetical
1227	1227	1.5	Aspartyl/Asparaginyl Beta-Hydroxylase
1237	blc	1.5	Outer membrane lipoprotein blc
1243	1243	1.7	Hypothetical Protein 1243
1246	yraR	1.7	Uncharacterized protein yraR
1261	1261	3.9	Hypothetical
1263	yrbG	1.8	Uncharacterized membrane protein MJ0091
1267	phaD	1.8	Probable K(+)/H(+) antiporter subunit D
1268	phaC	1.5	Probable K(+)/H(+) antiporter subunit C
1278	trpE	1.9	Anthranilate synthase component 1
1281	yggE	1.7	Hypothetical
1283	trpD	1.5	Anthranilate phosphoribosyltransferase
1299	1299	1.8	Hypothetical
1301	nudC	1.5	NADH pyrophosphatase
1316	1316	1.5	Hypothetical
1317	1317	1.7	Hypothetical
1320	aceK	1.9	Isocitrate dehydrogenase kinase/phosphatase
1325	gluP	2	Glucose/galactose transporter
1327	bglX	1.5	Periplasmic beta-glucosidase
1336	1336	2.1	Hypothetical
1337	1337	1.6	Hypothetical

1341	1341	1.5	Hypothetical
1344	1344	1.5	Hypothetical
1346	ynbC	1.5	Uncharacterized protein ynbC
1356	1356	1.5	Hypothetical
1360	aroQ	1.7	3-dehydroquinate dehydratase
1377	1377	1.7	Hypothetical Protein 1377
1378	1378	2.6	Hypothetical
1380	1380	1.7	Hypothetical
1381	mmr	1.8	Methylenomycin A resistance protein
1382	mauR	1.5	Mau operon transcriptional activator
1384	1384	1.7	Hypothetical
1385	yxjM	2.1	Sensor histidine kinase yxjM
1386	yxjL	1.5	Uncharacterized transcriptional regulatory protein yxjL
1387	opdE	1.7	Transcription regulatory protein opdE
1392	lytH	1.5	L-Ala--D-Glu endopeptidase
1393	1393	1.8	Hypothetical
1394	rob	1.9	Right origin-binding protein
1396	nat	2.2	Arylamine N-acetyltransferase
1397	tse	1.5	Methyl-accepting chemotaxis serine transducer
1398	uvrA	1.5	UvrABC system protein A
1404	groL	1.5	60 kDa chaperonin
1405	gcvA	2.2	Glycine cleavage system transcriptional activator
1410	yhaZ	2.3	Uncharacterized protein yhaZ
1411	1411	1.9	Hypothetical
1412	aroG	2.8	Phospho-2-dehydro-3-deoxyheptonate aldolase, Phe-sensitive
1417	yicG	1.9	UPF0126 membrane protein HI_1240
1418	yhbQ	1.5	UPF0213 protein XC_1086
1422	1422	1.9	Hypothetical
1428	yliJ	2.4	Uncharacterized GST-like protein yliJ
1429	priA	1.6	Primosomal protein N'
1431	1431	3.3	Hypothetical
1437	1437	2.1	Hypothetical
1450	wcaL	2.4	Glycosyl Transferase
1460	yjjJ	2.7	Putative kinase YjjJ
1462	1462	3.1	Hypothetical
1473	yafC	1.5	Uncharacterized HTH-type transcriptional regulator yafC
1480	ymdC	2.2	Uncharacterized protein YmdC
1483	yoeA	1.9	Probable multidrug resistance protein yoeA

1486	1486	1.5	Hypothetical Protein 1486
1491	yqhC	2.2	AraC Family Transcriptional Regulator
1493	ydfD	1.6	Uncharacterized HTH-type transcriptional regulator ydfD
1504	ywpD	1.6	Putative uncharacterized protein ywpD
1508	1508	1.7	Hypothetical
1512	glpD	2.2	Glycerol-3-phosphate dehydrogenase
1514	1514	1.6	Hypothetical
1533	atoC	1.8	Acetoacetate metabolism regulatory protein AtoC
1534	1534	2	Hypothetical
1538	macA	1.5	Probable macrolide-specific efflux protein macA
1543	1543	1.8	Radical SAM Domain-Containing Protein
1549	prlC	1.7	Oligopeptidase A
1550	hdhA	2.1	Uncharacterized oxidoreductase PA3106
1558	metI	1.5	Methionine import system permease protein metI
1559	vapI	1.8	Virulence-associated protein I
1561	1561	2	Hypothetical
1570	1570	1.5	Hypothetical
1572	hslV	1.7	ATP-dependent protease subunit HslV
1576	acrA	1.6	Acriflavine resistance protein A
1578	oprM	1.7	Outer membrane protein oprM
1579	1579	1.7	Hypothetical
1587	yjjG	1.7	HAD Family Hydrolase
1593	mrdA	1.5	Penicillin-binding protein 2
1612	1612	1.5	Hypothetical
1614	fabG	1.5	3-oxoacyl-[acyl-carrier-protein] reductase FabG
1619	glk	3.7	Glucokinase-like protein XF_1460
1620	gluP	2.1	Glucose/galactose transporter
1621	purR	1.5	HTH-type transcriptional repressor purR
1622	glmS	1.7	Glucosamine--fructose-6-phosphate aminotransferase [isomerizing]
1623	nagA	1.5	N-acetylglucosamine-6-phosphate deacetylase
1624	1624	1.6	Hypothetical
1628	1628	1.5	Hypothetical Protein 1628
1632	rhtB	2.4	Homoserine/homoserine lactone efflux protein
1635	1635	1.5	LuxR Family Transcriptional Regulator
1653	1653	1.7	Hypothetical Protein 1653
1659	1659	1.8	Hypothetical
1660	1660	1.9	Hypothetical
1665	rio2	1.8	RIO-type serine/threonine-protein kinase Rio2

1666	lytH	1.9	L-Ala--D-Glu endopeptidase
1667	ynhG	1.5	Putative L,D-transpeptidase YkuD
1668	cph2	1.8	Phytochrome-like protein cph2
1669	1669	4.4	Hypothetical
1672	yhcA	3	Uncharacterized MFS-type transporter yhcA
1673	emrA	8.8	Multidrug resistance protein A homolog
1685	1685	2.8	Hypothetical Protein 1685
1686	1686	2	Hypothetical
1689	rsgA	1.7	Putative ribosome biogenesis GTPase RsgA
1713	1713	30.6	Conserved Hypothetical Protein
1719	1719	2.5	Hypothetical Protein 1719
1726	tdcB	1.7	Threonine dehydratase catabolic
1727	1727	2.6	Acetolactate Synthase Isozyme II Small Subunit
1728	ilvG	1.9	Acetolactate synthase isozyme 2 large subunit
1729	ilvC	1.5	Ketol-acid reductoisomerase
1740	1740	5.2	Hypothetical
1743	fecR	1.5	Anti-FecI Sigma Factor FecR
1750	tonB	2.3	Protein tonB
1759	1759	2.6	Serine/Threonine Protein Kinase
1763	1763	1.7	Hypothetical
1764	gdh	1.6	Glucose 1-dehydrogenase
1767	1767	1.8	Putative potassium channel protein RPA4233
1783	pepQ	1.8	Xaa-Pro dipeptidase
1789	rph	1.6	Ribonuclease PH
1795	recG	1.6	ATP-dependent DNA helicase recG
1818	1818	1.5	Uncharacterized protein Rv1480/MT1527
1819	1819	1.5	Hypothetical Protein 1819
1820	1820	2	Uncharacterized protein BB_0173
1821	1821	1.7	Hypothetical
1831	blh	1.7	Beta-lactamase hydrolase-like protein
1844	1844	1.5	Hypothetical
1847	cysK	2.6	Cysteine synthase
1848	cysB	1.6	HTH-type transcriptional regulator CysB
1855	1855	2.6	Hypothetical
1867	1867	2.3	Hypothetical Protein 1867
1868	1868	1.6	Hypothetical Protein 1868
1869	1869	2.8	Hypothetical
1886	1886	4.6	Transposase

1891	yiaD	2.8	Uncharacterized protein Rv0899/MT0922
1895	btuB	2.4	Vitamin B12 transporter BtuB
1896	1896	1.6	Hypothetical
1899	1899	1.6	Hypothetical Protein 1899
1900	poxB	1.6	Pyruvate dehydrogenase [ubiquinone]
1904	mauG	2.6	Methylamine utilization protein MauG
1905	yddB	1.6	NB Dependent Receptor
1906	yddB	1.5	Uncharacterized protein yddB
1909	clpB	1.5	Chaperone protein ClpB
1910	mdeA	3	Methionine gamma-lyase
1913	fepA	1.9	Ferrienterobactin receptor
1916	1916	2.1	Beta-lactamase L2
1942	1942	1.6	Hypothetical Protein 1942
1944	1944	1.7	Hypothetical
1945	1945	1.8	Hypothetical Protein 1945
1948	1948	2	Hypothetical
1953	1953	70.9	Hypothetical Protein 1953
1957	copA	1.5	Copper resistance protein A
1964	relA	1.6	GTP pyrophosphokinase
1969	yoaA	1.5	Probable ATP-dependent helicase yoaA
1981	bioA	1.5	Adenosylmethionine-8-amino-7-oxononanoate aminotransferase
1993	alkJ	1.8	Alcohol dehydrogenase [acceptor]
1996	1996	1.5	Hypothetical
1997	fadE	1.5	Acyl-coenzyme A dehydrogenase
2009	yebA	1.6	Uncharacterized metalloprotease bbp_296
2013	2013	2.2	Hypothetical Protein 2013
2016	2016	1.7	Hypothetical Protein 2016
2022	2022	1.7	Hypothetical Protein 2022
2025	metL	1.9	Bifunctional aspartokinase/homoserine dehydrogenase 2
2030	yceJ	1.5	Cytochrome b561 homolog 2
2033	luxQ	1.6	Autoinducer 2 sensor kinase/phosphatase luxQ
2034	rpfC	1.8	Sensory/regulatory protein RpfC
2039	aroB	1.7	3-dehydroquinate synthase
2041	2041	1.6	Hypothetical
2046	prpR	1.7	Propionate catabolism operon regulatory protein
2052	prpD	1.5	2-methylcitrate dehydratase
2054	pbpC	1.8	Penicillin-binding protein 1C
2056	2056	1.5	Putative peroxiredoxin sll1621

2064	2064	1.5	Hypothetical
2073	bcr	1.5	Bicyclomycin resistance protein
2089	yhhW	1.6	Pirin-like protein CC_3178
2092	2092	1.6	Negative Regulator Of Sigma E Activity
2111	2111	2.1	Metallophosphoesterase
2112	lhr	5.2	Uncharacterized ATP-dependent helicase MJ0294
2113	lig	1.7	Probable DNA ligase
2114	lig	2	Probable DNA ligase
2126	ybiI	2.5	Uncharacterized protein YbiI
2131	2131	1.5	Hypothetical
2132	2132	1.5	Hypothetical
2136	phnA	1.5	Protein phnA
2137	ccmH	1.9	TPR Repeat-Containing Protein
2140	2140	2.1	Hypothetical
2142	mprF	1.8	Phosphatidylglycerol lysyltransferase
2144	rnfB	2.7	Electron transport complex protein rnfB
2156	cph2	1.5	Phytochrome-like protein cph2
2167	tonB	1.6	Protein tonB
2171	yhdP	1.5	Uncharacterized protein yhdP
2175	2175	1.5	Hypothetical
2176	ispA	1.7	Farnesyl diphosphate synthase
2178	tilS	1.5	tRNA(Ile)-lysidine synthase
2182	2182	1.7	Hypothetical
2188	zraR	1.6	Transcriptional regulatory protein zraR
2195	ybaZ	1.6	Methylated-DNA-(Protein)-Cysteine S-Methyltransferase
2207	2207	1.9	Hypothetical Protein 2207
2219	aroC	1.5	Chorismate synthase
2223	2223	1.6	Glyoxalase/Bleomycin Resistance Protein/Dioxygenase
2229	2229	1.6	Signal Recognition Particle-Docking Protein FtsY
2264	2264	2.4	Hypothetical
2267	cadC	2.1	Transcriptional Regulatory Protein-Like Protein
2283	betI	1.5	TetR Family Transcriptional Regulator
2286	putR	2.8	Proline dehydrogenase transcriptional activator
2287	rhtB	1.6	Homoserine/homoserine lactone efflux protein
2291	yafP	1.6	Acetyltransferase
2296	yiaT	1.7	Putative outer membrane protein yiaT
2301	slyA	2.6	MarR Family Transcriptional Regulator
2302	tetX	3.5	Tetracycline resistance protein from transposon Tn4351/Tn4400

2319	2319	2.2	Hypothetical
2335	yjeF	1.5	Uncharacterized protein yjeF
2347	suhB	1.5	Inositol-1-monophosphatase
2363	2363	1.6	Hypothetical
2366	argE	1.9	Acetylornithine deacetylase
2368	2368	1.6	GCN5-Related N-Acetyltransferase
2376	pgaB	1.5	Poly-beta-1,6-N-acetyl-D-glucosamine N-deacetylase
2383	cydC	1.5	ATP-binding/permease protein CydC
2389	2389	1.6	Hypothetical Protein 2389
2392	cycH	1.6	Cytochrome c-type biogenesis protein CycH
2413	lacF	1.5	Lactose transport system permease protein lacF
2430	2430	2.4	Endonuclease/Exonuclease/Phosphatase Family Protein
2444	ydfG	7.9	Uncharacterized protein ydfG
2459	ycfJ	1.6	Uncharacterized protein ycfJ
2475	2475	2.4	Hypothetical
2490	metH	2.1	Methionine synthase
2491	ubiE	1.7	Uncharacterized 37.1 kDa protein in transposon TN4556
2498	ppnK	2.5	Probable inorganic polyphosphate/ATP-NAD kinase
2500	2500	1.5	Hypothetical
2522	phnO	1.5	Acetyltransferase Gnat Family
2525	mtnA	1.5	Methylthioribose-1-phosphate isomerase
2527	sldA	1.5	Glycerol dehydrogenase large subunit
2529	2529	1.5	Hypothetical
2531	2531	2	Hypothetical Protein 2531
2534	2534	1.5	Hypothetical Protein 2534
2540	2540	2.2	Hypothetical Protein 2540
2547	2547	1.6	Hypothetical Protein 2547
2551	hutU	1.8	Urocanate hydratase
2553	hutH	1.9	Histidine ammonia-lyase
2557	2557	1.9	Hypothetical Protein 2557
2586	2586	1.7	Hypothetical Protein 2586
2597	acrR	1.5	TetR Family Transcriptional Regulator
2608	ygfF	1.6	Uncharacterized oxidoreductase MexAM1_META1p0182
2609	2609	2.2	Hypothetical
2611	2611	1.8	Hypothetical
2626	2626	1.5	Hypothetical
2628	2628	1.6	Hypothetical
2630	2630	1.8	Glyoxalase/Bleomycin Resistance Protein/Dioxygenase

2637	yiiP	1.7	Cation Diffusion Facilitator Family Transporter
2638	2638	1.6	Response Regulator Receiver Protein
2639	yoaM	1.7	UPF0361 protein yoaM
2641	lhr	1.5	Probable ATP-dependent helicase lhr
2645	2645	2	Hypothetical Protein 2645
2646	araJ	1.9	Protein AraJ
2648	2648	1.6	Hypothetical
2651	tadA	1.7	tRNA-specific adenosine deaminase
2653	2653	1.5	Hypothetical
2654	nemA	1.6	N-ethylmaleimide reductase
2659	2659	1.7	Hypothetical
2668	yegE	1.6	Probable diguanylate cyclase YegE
2676	rhaR	2.6	AraC Family Transcriptional Regulator
2680	adiC	1.5	Arginine/agmatine antiporter
2686	map	1.5	Methionine aminopeptidase
2688	ypdA	1.8	Inner membrane protein ypdA
2693	2693	2.3	Hypothetical Protein 2693
2694	yafC	1.7	Uncharacterized HTH-type transcriptional regulator yafC
2696	2696	1.7	Hypothetical Protein 2696
2698	2698	1.5	Hypothetical
2704	2704	3.1	Hypothetical
2706	yggS	1.6	UPF0001 protein aq_274
2713	yqhC	1.9	AraC Family Transcriptional Regulator
2714	ygaY	2.1	Uncharacterized transporter ygaY
2715	ytfH	1.6	Uncharacterized HTH-type transcriptional regulator Rv3095/MT3179
2718	emrB	3.5	Multidrug resistance protein B homolog
2719	2719	3.5	Hypothetical
2725	sapB	1.5	Protein SapB
2727	lldP	2.6	L-lactate permease
2728	lldR	2	Putative L-lactate dehydrogenase operon regulatory protein
2729	lldD	1.7	L-lactate dehydrogenase [cytochrome]
2730	dld	1.7	D-lactate dehydrogenase
2790	2790	1.8	Hypothetical
2795	cdh	1.5	CDP-diacylglycerol pyrophosphatase
2796	2796	2.7	Hypothetical
2799	rhtC	3.5	Threonine efflux protein
2802	2802	2.2	Hypothetical Protein 2802
2803	nylB'	1.6	6-aminohexanoate-dimer hydrolase

2818	yadA	2.4	Adhesin yadA
2823	2823	1.5	6-hydroxy-D-nicotine oxidase
2827	2827	2	Heavy Metal Transport/Detoxification Protein
2832	yhbO	1.5	ThiJ/PfpI Domain-Containing Protein
2835	2835	1.9	Hypothetical Protein 2835
2840	cyoA	1.5	Ubiquinol oxidase subunit 2
2843	cyoD	2.3	Cytochrome o ubiquinol oxidase protein CyoD
2844	2844	2.2	Putative lipoprotein Lxx21020
2847	2847	1.7	Methyltransferase
2849	mntH	1.6	Probable manganese transport protein mntH
2850	ptxR	2.4	HTH-type transcriptional regulator ptxR
2851	ygaY	2.4	Uncharacterized transporter ygaY
2852	yxjL	1.7	Uncharacterized transcriptional regulatory protein yxjL
2853	ydfH	2.5	Sensor histidine kinase ydfH
2854	2854	2.6	Hypothetical Protein 2854
2855	rhaR	1.7	AraC Family Transcriptional Regulator
2856	ycaC	1.5	Uncharacterized protein ycaC
2857	2857	2	DoxD-Like Family Membrane Protein
2859	2859	1.7	Hypothetical
2860	2860	1.5	Hypothetical
2868	2868	4.8	Hypothetical
2869	yhfK	1.5	Uncharacterized sugar epimerase yhfK
2872	mdtJ	6.3	Spermidine export protein mdtJ
2873	mdtI	7.6	Spermidine export protein mdtI
2874	prpB	2.4	Oxaloacetate decarboxylase
2875	ybjJ	2.6	Uncharacterized MFS-type transporter YPO1221/y2967/YP_0917
2877	yhjC	2.8	Uncharacterized HTH-type transcriptional regulator yhjC
2881	2881	1.5	Flavodoxin
2883	nrdA	1.9	Ribonucleoside-diphosphate reductase subunit alpha
2884	yflS	2	Putative malate transporter yflS
2893	2893	1.9	Disulphide-Isomerase
2897	mgtA	1.8	Magnesium-transporting ATPase, P-type 1
2898	2898	2.2	Hypothetical
2899	2899	1.9	Hypothetical
2901	2901	1.7	Hypothetical
2902	2902	1.9	Hypothetical
2913	yuaQ	2.1	Uncharacterized protein YuaQ
2917	2917	2	Hypothetical Protein 2917

2918	2918	1.5	Hypothetical Protein 2918
2931	2931	1.5	Hypothetical
2936	2936	1.8	Hypothetical
2938	2938	1.5	Hypothetical Protein 2938
2939	gcvA	1.5	Glycine cleavage system transcriptional activator
2942	cbg-1	1.7	Beta-glucosidase
2946	ccpA	1.6	Glucose-resistance amylase regulator
2950	xylB	1.5	Xylulose kinase
2955	ccpA	1.5	Catabolite control protein A
2957	2957	1.5	Hypothetical
2959	phaZ1	1.7	Poly(3-hydroxyalkanoate) depolymerase C
2961	fyuA	1.5	Pesticin receptor
2962	ybfF	1.5	Alpha/Beta Hydrolase Fold Protein
2963	maoC	3.1	Probable enoyl-CoA hydratase 1
2964	torS	1.7	Sensor protein torS
2966	2966	2	D-(-)-3-hydroxybutyrate oligomer hydrolase
2973	2973	2.3	Hypothetical
2977	2977	1.6	Hypothetical
2982	aer	2.3	Aerotaxis receptor
2983	cysJ	3	Sulfite reductase [NADPH] flavoprotein alpha-component
2984	fnr	2	Fumarate and nitrate reduction regulatory protein
2985	2985	1.6	Hypothetical
2997	ycaK	2.6	Uncharacterized NAD(P)H oxidoreductase HI_1544
3001	3001	2.8	Helix-Turn-Helix Type
3006	crcB	2.1	Protein CrcB homolog
3012	3012	2.3	Hypothetical
3081	3081	1.6	Hypothetical
3130	ycaK	5.2	Uncharacterized NAD(P)H oxidoreductase HI_1544
3141	3141	1.6	Hypothetical
3160	scrK	1.7	Fructokinase
3162	purR	2.2	HTH-type transcriptional repressor purR
3166	ybiK	1.7	N(4)-(Beta-N-acetylglucosaminyl)-L-asparaginase
3168	3168	1.7	Hypothetical Protein 3168
3171	hisF	1.7	Imidazole glycerol phosphate synthase subunit hisF
3172	hisA	1.9	1-(5-phosphoribosyl)-5-[(5- phosphoribosylamino)methylideneamino] imidazole-4-carboxamide isomerase
3184	thrA	2.1	Bifunctional aspartokinase/homoserine dehydrogenase 1
3186	ykgD	1.5	Uncharacterized HTH-type transcriptional regulator ykgD
3190	3190	1.8	Hypothetical

3192	3192	3.5	Glycosyl Transferase Family Protein
3194	3194	3.1	LmbE Family Protein
3202	3202	1.7	Hypothetical Protein 3202
3225	pyrD	1.6	Dihydroorotate dehydrogenase (quinone)
3227	sam	1.5	S-adenosylmethionine uptake transporter
3235	neo	3.1	Aminoglycoside 3'-phosphotransferase
3236	3236	2.8	Hypothetical
3238	3238	2.4	Hypothetical
3239	fliY	2.2	Secreted Protein
3240	gluA	2.8	Glutamate transport ATP-binding protein GluA
3241	3241	7.9	Acetyltransferase
3242	hipO	1.7	Hippurate hydrolase
3243	moxC	1.7	Putative monooxygenase moxC
3244	ytmO	1.9	Uncharacterized protein ytmO
3245	3245	1.7	Hypothetical
3246	yhbW	2	Monooxygenase
3247	msuE	3.2	FMN reductase
3249	glxA	3.1	HTH-type transcriptional regulator glxA
3250	3250	2.2	Hypothetical Protein 3250
3277	rpmJ2	1.5	50S ribosomal protein L36 2
3281	pat	1.5	Phosphinothricin N-acetyltransferase
3289	yeiE	2.8	Uncharacterized HTH-type transcriptional regulator yeiE
3290	yeiH	1.5	UPF0324 membrane protein plu2856
3299	pcaG	2	Protocatechuate 3,4-dioxygenase alpha chain
3301	pcaF	1.7	Beta-ketoadipyl-CoA thiolase
3304	benK	1.6	Benzoate transport protein
3305	quiB	1.6	Catabolic 3-dehydroquinate dehydratase
3309	yfcG	1.5	Glutathione S-Transferase
3313	3313	2.6	Hypothetical Protein 3313
3318	3318	3.1	Facilitator Superfamily Protein
3322	3322	1.7	Transposase
3333	yigZ	1.5	IMPACT family member in pol 5'region
3334	msbA	1.6	Lipid A export ATP-binding/permease protein MsbA
3338	dnaJ	1.5	Chaperone protein DnaJ
3339	dnaK	1.8	Chaperone protein DnaK
3340	3340	1.5	Hypothetical Protein 3340
3342	hrcA	2	Heat-inducible transcription repressor hrcA
3344	yafC	2	Uncharacterized HTH-type transcriptional regulator yafC

3345	3345	2.1	Secreted Protein
3352	3352	1.5	Acetyltransferase
3354	3354	2.6	Hypothetical
3357	3357	1.7	Acetyltransferase
3358	3358	1.5	Hypothetical
3367	3367	1.5	Hypothetical
3369	arpC	1.7	Antibiotic efflux pump outer membrane protein ArpC
3370	ucpA	1.7	Monensin polyketide synthase putative ketoacyl reductase
3374	3374	2.6	Hypothetical Protein 3374
3377	oar	2.2	Protein oar
3379	phrB	1.6	Deoxyribodipyrimidine photo-lyase
3391	3391	5.7	Metallophosphoesterase
3393	cirA	2.1	NB-Dependent Receptor
3395	psiF	1.8	PsiF Repeat-Containing Protein
3403	3403	2.4	Hypothetical
3408	lolD	1.5	Lipoprotein-releasing system ATP-binding protein LolD
3420	glk	1.5	Glucokinase
3423	eda	1.5	2-dehydro-3-deoxy-phosphogluconate aldolase
3443	3443	2.1	Hypothetical
3446	3446	2.9	Hypothetical
3450	pld1	2.3	Pyridoxal 4-dehydrogenase
3457	recX	1.5	Regulatory protein recX
3460	ubiB	1.9	Probable ubiquinone biosynthesis protein UbiB
3464	miaA	1.5	tRNA dimethylallyltransferase
3482	3482	1.5	Hypothetical Protein 3482
3494	yhbW	1.9	Uncharacterized protein yhbW
3501	vipA	1.6	Vi polysaccharide biosynthesis protein vipA/tviB
3508	3508	1.5	Hypothetical
3512	rdxA	1.7	Protein rdxA
3516	3516	1.5	Conserved Hypothetical Protein
3519	3519	2.6	Hypothetical Protein 3519
3530	3530	1.5	Fimbrial Biogenesis Protein
3546	3546	1.5	Hypothetical
3557	ybhG	2	UPF0194 membrane protein YPA_1093
3559	3559	1.7	Phosphoesterase PA-Phosphatase
3568	3568	1.7	Rhs Element Vgr Protein
3576	sad	1.6	Succinate semialdehyde dehydrogenase [NAD(P)+] Sad
3578	potH	1.5	Putrescine transport system permease protein PotH

3582	puuD	2.1	Gamma-glutamyl-gamma-aminobutyrate hydrolase
3583	ordL	2.1	Probable oxidoreductase ordL
3593	puuC	3	Aldehyde dehydrogenase PuuC
3599	3599	1.8	Uncharacterized protein HI_1420
3600	cefD	1.5	Isopenicillin N epimerase
3607	3607	2.4	Hypothetical
3609	sseA	2	Probable 3-mercaptopyruvate sulfurtransferase
3612	paaF	1.5	3-hydroxypropionyl-coenzyme A dehydratase
3617	3617	1.6	Hypothetical
3622	pstS	1.7	Phosphate-binding protein pstS
3624	pstA	1.6	Phosphate transport system permease protein pstA
3625	pstB	1.9	Phosphate import ATP-binding protein PstB
3626	phoU	2.8	Phosphate transport system protein phoU
3628	3628	1.9	UPF0276 protein PSEEN3355
3648	bfr	1.5	Bacterioferritin
3652	ybeQ	2.2	Hypothetical
3660	3660	1.5	Pili Assembly Chaperone
3661	yraJ	1.5	Uncharacterized outer membrane usher protein yraJ
3662	3662	1.7	Spore Coat Protein U
3663	3663	1.7	Hypothetical Protein 3663
3693	gcvA	1.7	Glycine cleavage system transcriptional activator
3695	3695	1.5	Hypothetical
3696	3696	1.5	Hypothetical
3700	3700	1.9	Glyoxalase/Bleomycin Resistance Protein/Dioxygenase
3702	3702	2.6	Hypothetical
3709	ybcJ	1.8	Hypothetical
3710	visC	1.6	Protein visC
3722	3722	1.7	Hypothetical Protein 3722
3728	gltX	1.5	Glutamyl-tRNA synthetase
3743	apaH	1.8	Metallophosphoesterase
3744	desA3	2.7	Stearoyl-CoA 9-desaturase
3745	paaE	2.3	Stearoyl-CoA 9-desaturase electron transfer partner
3747	3747	2	Myosin-Cross-Reactive Antigen
3751	ybhR	1.8	Hypothetical
3754	3754	3.9	BaRNAse Inhibitor
3768	3768	1.5	Hypothetical
3769	3769	1.5	Probable nitronate monooxygenase
3789	treA	1.5	Periplasmic trehalase

3790	3790	1.6	YhhN Family Protein
3793	lspA	1.8	Lipoprotein signal peptidase
3805	echA17	1.9	Probable enoyl-CoA hydratase echA17
3811	3811	1.6	Hypothetical Protein 3811
3836	yggA	2.4	Putative amino-acid transporter Rv1986/MT2040
3837	iciA	3.1	Uncharacterized HTH-type transcriptional regulator Rv1985c/MT2039
3838	3838	1.5	Hypothetical
3846	3846	2.1	Malonate Decarboxylase Alpha Subunit
3850	mdcG	1.9	Phosphoribosyl-dephospho-CoA transferase
3851	mdcB	1.7	Probable 2-(5''-triphosphoribosyl)-3'-dephosphocoenzyme-A synthase
3856	3856	1.5	Hypothetical
3858	yecE	1.6	Hypothetical
3860	3860	1.7	Hypothetical Protein 3860
3861	insK	5.2	Putative transposase InsK for insertion sequence element IS150
3863	3863	1.5	Hypothetical
3864	3864	1.6	NB-Dependent Receptor
3865	fecR	2.9	Anti-FecI Sigma Factor FecR
3866	fecI	1.5	RNA Polymerase Sigma Factor
3869	3869	2.9	Hypothetical Protein 3869
3870	besA	1.9	Ferri-bacillibactin esterase BesA
3873	3873	2	Hypothetical
3874	3874	2.6	Acetyltransferase
3876	3876	1.5	Uncharacterized protein Rv0906/MT0929
3883	3883	1.7	Xylose Isomerase Domain-Containing Protein
3884	yegT	2	Putative nucleoside transporter yegT
3885	ydgJ	2.8	Uncharacterized oxidoreductase y4hM
3886	3886	1.5	Xylose Isomerase Domain-Containing Protein
3887	3887	1.8	Cytochrome C Subfamily Protein
3888	3888	1.7	Hypothetical
3889	3889	1.9	Hypothetical
3890	3890	2.1	Gluconate 2-dehydrogenase flavoprotein
3891	ygbM	2	Uncharacterized 28.3 kDa protein in gbd 5'region
3893	3893	1.9	RTX xin-Activating Protein C
3897	celR	1.5	HTH-type transcriptional regulator CelR
3898	3898	2.2	Hypothetical
3906	3906	2	Hypothetical
3909	aglA	2.5	Probable alpha-glucosidase
3912	3912	1.6	Hypothetical

3919	3919	3.9	Hypothetical
3921	3921	1.6	Hypothetical
3925	sufB	1.5	UPF0051 protein slr0074
3928	csd	1.8	Probable cysteine desulfurase
3937	piv	4.1	Pilin gene-inverting protein
3943	ves	1.9	Protein ves
3944	3944	1.8	Hypothetical
3947	ymgE	1.9	Hypothetical
3956	purN	2.3	Phosphoribosylglycinamide formyltransferase
3957	3957	1.6	Hypothetical
3969	ptsN	1.5	Nitrogen regulatory protein
3970	hprK	1.6	HPr kinase/phosphorylase
3977	3977	1.7	Phospholipase/Carboxylesterase
3978	yafM	2.4	Hypothetical
3988	yccK	1.6	Uncharacterized oxidoreductase YccK
4000	4000	1.7	Glutathione-Dependent Formaldehyde-Activating GFA
4004	yafC	2.2	Uncharacterized HTH-type transcriptional regulator yafC
4008	fabG	3.8	Putative short-chain type dehydrogenase/reductase Rv0148
4009	4009	2	Hypothetical Protein 4009
4011	4011	2.1	Conserved Hypothetical Protein
4016	yehP	1.9	Uncharacterized protein yehP
4049	4049	2.3	Hypothetical
4051	4051	2	Hypothetical
4068	4068	14.1	Hypothetical Protein 4068
4072	yddQ	2.3	Uncharacterized isochorismatase family protein yddQ
4083	pgpB	1.7	Phosphoesterase PA-Phosphatase Related Protein
4085	4085	1.5	Hypothetical Protein 4085
4093	cobC	1.5	Alpha-ribazole phosphatase
4102	qor	2.4	Zinc-type alcohol dehydrogenase-like protein SAV2186
4103	4103	2.4	Uncharacterized protein HI_0522
4177	4177	1.5	Hypothetical
4188	pntB	1.6	NAD(P) transhydrogenase subunit beta
4190	4190	2.2	RNA Polymerase Sigma-24 Subunit ECF Subfamily
4191	4191	2	Hypothetical Protein 4191
4193	pntAA	1.5	NAD(P) transhydrogenase subunit alpha part 1
4207	oxyR	1.7	Hydrogen peroxide-inducible genes activator
4209	tal	1.5	Transaldolase
4210	msrA	1.5	Peptide methionine sulfoxide reductase MsrA

4211	4211	3.1	Hypothetical
4215	4215	1.5	Hypothetical
4216	ubiF	1.6	2-octaprenyl-3-methyl-6-methoxy-1,4-benzoquinol hydroxylase
4217	ubiH	1.8	2-octaprenyl-6-methoxyphenol hydroxylase
4219	4219	1.7	Cob(I)yrinic acid a,c-diamide adenosyltransferase
4241	ybbA	1.7	Uncharacterized ABC transporter ATP-binding protein YbbA
4251	4251	1.6	Hypothetical Protein 4251
4253	4253	1.7	Hypothetical Protein 4253
4254	4254	2.8	Hypothetical Protein 4254
4259	4259	2.8	Membrane-Bound Metal-Dependent Hydrolase
4266	ybaK	1.5	Cys-tRNA(Pro)/Cys-tRNA(Cys) deacylase ybaK
4269	4269	1.9	Hypothetical
4274	nudG	1.5	CTP pyrophosphohydrolase
4318	4318	1.9	Hypothetical
4321	purP	1.5	Probable adenine permease PurP
4324	4324	2.2	Cell Morphology Protein
4336	4336	1.9	Hypothetical
4344	xpsH	1.6	General secretion pathway protein H
4347	xpsE	1.6	General secretion pathway protein E
4349	yadA	1.9	Adhesin yadA
4351	chiC	1.5	Chitinase C
4354	4354	1.7	Hypothetical
4363	4363	1.7	Hypothetical
4370	yrbE	1.8	Probable ABC transporter permease protein RT0041
4400	4400	1.5	Outer Membrane Protein
4401	4401	1.5	Outer Membrane Protein
4406	4406	2.1	Peptidase
4407	btuB	3.1	Vitamin B12 transporter BtuB
4411	sasA	2.8	Adaptive-response sensory-kinase sasA
4412	yclJ	1.6	Uncharacterized transcriptional regulatory protein yclJ
4414	dipZ	2.4	Protein dipZ
4416	yjiR	2.2	Uncharacterized HTH-type transcriptional regulator yjiR
4423	ygiP	1.8	Uncharacterized HTH-type transcriptional regulator HI_1364
4424	ywnB	1.6	Uncharacterized protein ywnB
4450	ygiD	1.5	Uncharacterized protein ygiD
4451	4451	13.1	DoxX Family Protein
4454	pepO	1.7	Neutral endopeptidase
4459	yehU	1.5	Inner membrane protein yehU

4464	4464	1.6	Hypothetical
4465	4465	1.7	Uncharacterized protein R00369
4469	cycA	1.8	D-serine/D-alanine/glycine transporter
4470	ylaB	1.5	Uncharacterized protein YlaB
4472	sotB	3.3	Probable sugar efflux transporter
4473	yafC	2.1	Uncharacterized HTH-type transcriptional regulator yafC
4474	ydiF	2.2	Uncharacterized ABC transporter ATP-binding protein YdiF
4475	yciC	1.6	Putative metal chaperone YciC
4476	4476	3.1	Hypothetical
4477	4477	4.4	Hypothetical Protein 4477
4478	4478	1.6	Hypothetical
4479	yafM	1.5	Uncharacterized protein yafM
4483	4483	1.7	Conserved Hypothetical Protein
4485	4485	2.1	Hypothetical Protein 4485
4487	4487	1.8	Peptidase
4496	4496	1.6	Hypothetical
4519	yqjI	1.5	Uncharacterized protein yqjI
4526	4526	2.7	Hypothetical
4531	4531	2.2	Hypothetical Protein 4531
4532	glnE	1.6	Glutamate-ammonia-ligase adenylyltransferase
4535	4535	2.3	Hypothetical
4547	yfhH	1.7	Uncharacterized HTH-type transcriptional regulator yfhH
4550	4550	1.7	Hypothetical
4566	4566	1.7	Hypothetical
4594	4594	80.8	Hypothetical Protein 4594
4595	kdpA	1.9	Potassium-transporting ATPase A chain
4596	kdpB	2	Potassium-transporting ATPase B chain
4598	kdpD	2.9	Sensor protein KdpD
4599	kdpE	1.7	Transcriptional regulatory protein KdpE
4601	dut	1.6	Deoxyuridine 5'-triphosphate nucleotidohydrolase
4608	4608	1.8	Glyoxalase/Bleomycin Resistance Protein/Dioxygenase
4609	sdh	1.5	Serine 3-dehydrogenase
4610	4610	5.7	Hypothetical
4618	aly	2.1	Alginate lyase
4622	4622	1.7	Secreted Protein
4624	yefC	2	NUDIX Hydrolase
4625	ycfQ	2.5	Uncharacterized HTH-type transcriptional regulator ycfQ
4628	lip2	2.4	Lipase 2

4630	ybaA	1.5	Uncharacterized protein ybaA
4631	ptpA	1.9	Prolyl tripeptidyl peptidase
4643	degQ	1.7	Protease degQ
4644	4644	1.5	Hypothetical
4646	4646	1.5	Hypothetical
4651	srpA	1.9	Protein SrpA
4652	yodB	1.5	Cytochrome b561 homolog 1
4657	yvfR	1.9	Uncharacterized ABC transporter ATP-binding protein YvfR
4658	macA	1.6	Probable macrolide-specific efflux protein macA
4659	yknY	1.7	Uncharacterized ABC transporter ATP-binding protein YknY
4672	4672	3.2	Hypothetical
4687	4687	1.6	Hypothetical Protein 4687
4690	mmsB	1.7	3-hydroxyisobutyrate dehydrogenase
4691	yngF	1.7	Putative enoyl-CoA hydratase/isomerase yngF
4692	crt	2	3-hydroxybutyryl-CoA dehydratase
4693	acdA	1.6	Acyl-CoA dehydrogenase
4694	mmsA	1.5	Methylmalonate-semialdehyde dehydrogenase [acylating]
4695	4695	1.6	Uncharacterized HTH-type transcriptional regulator Rv0465c/MT0481
4696	yfbO	1.6	Hypothetical
4706	4706	2.2	Hypothetical
4715	metE	1.5	5-methyltetrahydropteroyltriglutamate-- homocysteine methyltransferase
4718	purU	2.2	Formyltetrahydrofolate deformylase
4725	4725	2.2	Hypothetical
4729	4729	2	Hypothetical
4749	mls	2.2	Malate synthase
4751	ynfL	1.7	Uncharacterized HTH-type transcriptional regulator ynfL
4752	4752	2.1	Hypothetical
4754	lepB	1.5	Signal peptidase I
4755	4755	22.8	Hypothetical
4756	4756	12.4	Hypothetical
4777	yhbS	1.9	Uncharacterized N-acetyltransferase YhbS
4779	4779	1.5	Cytochrome c2
4783	4783	2.1	Aminoglycoside Phosphotransferase
4788	plcN	1.7	Non-hemolytic phospholipase C
4791	nolG	1.6	Nodulation protein nolG
4819	hemD	2.5	Uroporphyrinogen-III Synthase
4820	4820	1.5	Hypothetical
4827	ntrB	1.9	Nitrogen regulation protein ntrB

4832	trxB	2	Thioredoxin reductase
4835	amtB	2.3	Ammonia channel
4836	glnB	1.7	Nitrogen regulatory protein P-II
4845	ydiI	2.4	Putative esterase PA1618
4855	hipA	1.5	Putative kinase Y4mE
4863	4863	3.2	Hypothetical
4865	4865	2.1	Hypothetical
4867	yrfF	1.8	Putative membrane protein igaA homolog
4868	4868	2.4	Hypothetical
4871	yprB	1.7	Uncharacterized protein yprB
4873	4873	1.5	Peptidase
4881	gltB	1.8	Glutamate synthase [NADPH] large chain
4883	4883	2.2	Hypothetical Protein 4883
4884	mgsA	2.2	Methylglyoxal synthase
4891	4891	3.1	Trypsin
4899	sps1	2	Probable serine/threonine-protein kinase Sps1
4903	4903	1.9	Hypothetical
4904	4904	1.5	Fatty Acid Desaturase
4906	4906	2.3	Hypothetical
4910	4910	1.5	Uncharacterized protein M6_Spy0233
4911	ylbK	1.6	Uncharacterized NTE family protein ylbK
4915	4915	2.3	Rhs Element Vgr Protein
4918	4918	2	Hypothetical
4925	yiaN	2.2	Uncharacterized protein y4mL
4926	4926	9.6	C4-Dicarboxylate Transport Small Permease Component
4929	xynB	1.6	Endo-1,4-beta-xylanase B
4931	yesY	1.6	Probable rhamnogalacturonan acetylesterase yesY
4934	4934	1.9	Hypothetical
4937	kdgK	2	2-dehydro-3-deoxygluconokinase
4947	4947	2.5	Hypothetical
4949	yncD	1.5	Probable tonB-dependent receptor yncD
4959	yknY	1.7	Uncharacterized ABC transporter ATP-binding protein YknY
4977	recF	1.9	DNA replication and repair protein recF
4988	4988	1.5	Hypothetical
4989	4989	1.6	Uncharacterized protein Rv1337/MT1378
4995	4995	2	Hypothetical
5001	qor	1.6	Zinc-type alcohol dehydrogenase-like protein SERP1785
5005	5005	1.6	Hypothetical

5012	yrbF	1.9	Uncharacterized ABC transporter ATP-binding protein HI_1087
5013	recC	1.7	Exodeoxyribonuclease V gamma chain
5014	recB	1.7	Exodeoxyribonuclease V beta chain
5017	ydhK	2.1	Uncharacterized transporter YdhK
5018	nodT	3.5	Nodulation protein T
5019	ydhJ	1.8	Uncharacterized protein ydhJ
